# Biogenic Selenium Nanoparticle-Based Nanocomposite Hydrogel for Dual Delivery of Curcumin and Simvastatin for Topical Wound Healing Applications

**DOI:** 10.3390/pharmaceutics18101257

**Published:** 2026-10-05

**Authors:** Nahla A. Kasem, Hatem A. Sarhan, Mahmoud Mostafa, Heba A. Hofny, Deiaa E. Elsayed Abouzed, Mahmoud M. A. Elsayed, Mahmoud Elkot Mostafa

**Affiliations:** 1 Department of Pharmaceutics and Clinical Pharmacy, Faculty of Pharmacy, Sohag University, Sohag 82524, Egypt; nahla.ahmed@pharm.sohag.edu.eg (N.A.K.); mahmoudalmenshawy@pharm.sohag.edu.eg (M.M.A.E.); 2 Department of Pharmaceutics and Clinical Pharmacy, Faculty of Pharmacy, Sohag National University, Sohag 82524, Egypt; 3 Department of Pharmaceutics, Faculty of Pharmacy, Minia University, Minia 61519, Egypt; hatem.sarhan@mu.edu.eg (H.A.S.); mahmoud_mohamed@mu.edu.eg (M.M.); 4 Department of Pharmaceutical Chemistry, Faculty of Pharmacy, Sohag University, Sohag 82524, Egypt; heba.atef@pharm.sohag.edu.eg; 5 Department of Pharmaceutical Chemistry, Faculty of Pharmacy, Sohag National University, Sohag 82524, Egypt; 6 Department of Pharmacology and Toxicology, Faculty of Pharmacology, Sohag University, Sohag 82524, Egypt; deiaaabozed@pharm.sohag.edu.eg; 7 Department of Pharmaceutics and Industrial Pharmacy, Faculty of Pharmacy, New Valley University, Al-Kharga 72511, Egypt; 8 Department of Pharmaceutics and Industrial Pharmacy, Faculty of Pharmacy, New Valley National University, Al-Kharga 72511, Egypt

**Keywords:** wound healing, selenium nanoparticles, Box–Behnken optimization, dual-drug delivery system, molecular docking simulation

## Abstract

**Background:** Chronic and ischemic wounds are dysregulated, causing inflammation, oxidative stress, and bacterial colonization. This work developed a bionanocomposite hydrogel using biogenic selenium nanoparticles (SeNPs) to encapsulate and topically distribute two weakly water-soluble medicines, curcumin (Curc) and simvastatin (SMV), to improve wound healing. **Methods:** Green production of biogenic SeNPs utilizing Psidium guajava (guava) leaf extract. Based on entrapment efficiency (%EE; E1) and cumulative drug release after 2 and 8 h (E2) and (E3), a two-factor, three-level Box–Behnken design selected the SeNP-to-drug weight ratio (C_1_) and ethanol concentration (C_2_). The selected nanoconstruct was integrated into a 2% *w*/*v* Carbopol 934 hydrogel and characterized by UV-vis, FTIR, SEM, and DLS. In a 14-day full-thickness excisional wound model in Wistar rats, wound contraction, tissue remodeling, inflammatory responses, and healing kinetics were examined to determine its therapeutic efficiency. **Results:** Formulation F9 had the highest overall attractiveness (0.998) and %EE values of 94.71% for SMV and 93.92% for Curc. DLS showed a hydrodynamic particle size of 177.7 nm and PDI of 0.41. Both medications were successfully incorporated without chemical incompatibility based on FTIR analysis. The selected nanogel maintained a skin-friendly pH of 6.2 ± 0.1 and sustained biphasic drug release. In vivo, the half-dose nanogel showed an enhanced wound-healing response, accelerating wound contraction and achieving complete epithelialization in 14 ± 1.06 days (*p* < 0.05) compared to 22 ± 2.5 days for the sham control, corresponding to a 36% reduction in healing time. Histopathology showed epidermal thickness restoration and well-organized collagen deposition. In addition, therapy dramatically decreased the proinflammatory biomarkers IL-1β, MMP-9, and COX-2 and increased IL-10 levels without causing tissue discomfort. **Conclusions:** The biogenic SeNP-based nanogel delivers Curc and SMV locally with prolonged drug release and anti-inflammatory and tissue-remodeling effects. The half-dose combination accelerated wound healing while retaining tissue compatibility, suggesting this green nanocomposite hydrogel could be a promising topical method for advanced cutaneous wound therapy.

## 1. Introduction

**Figure 1:**
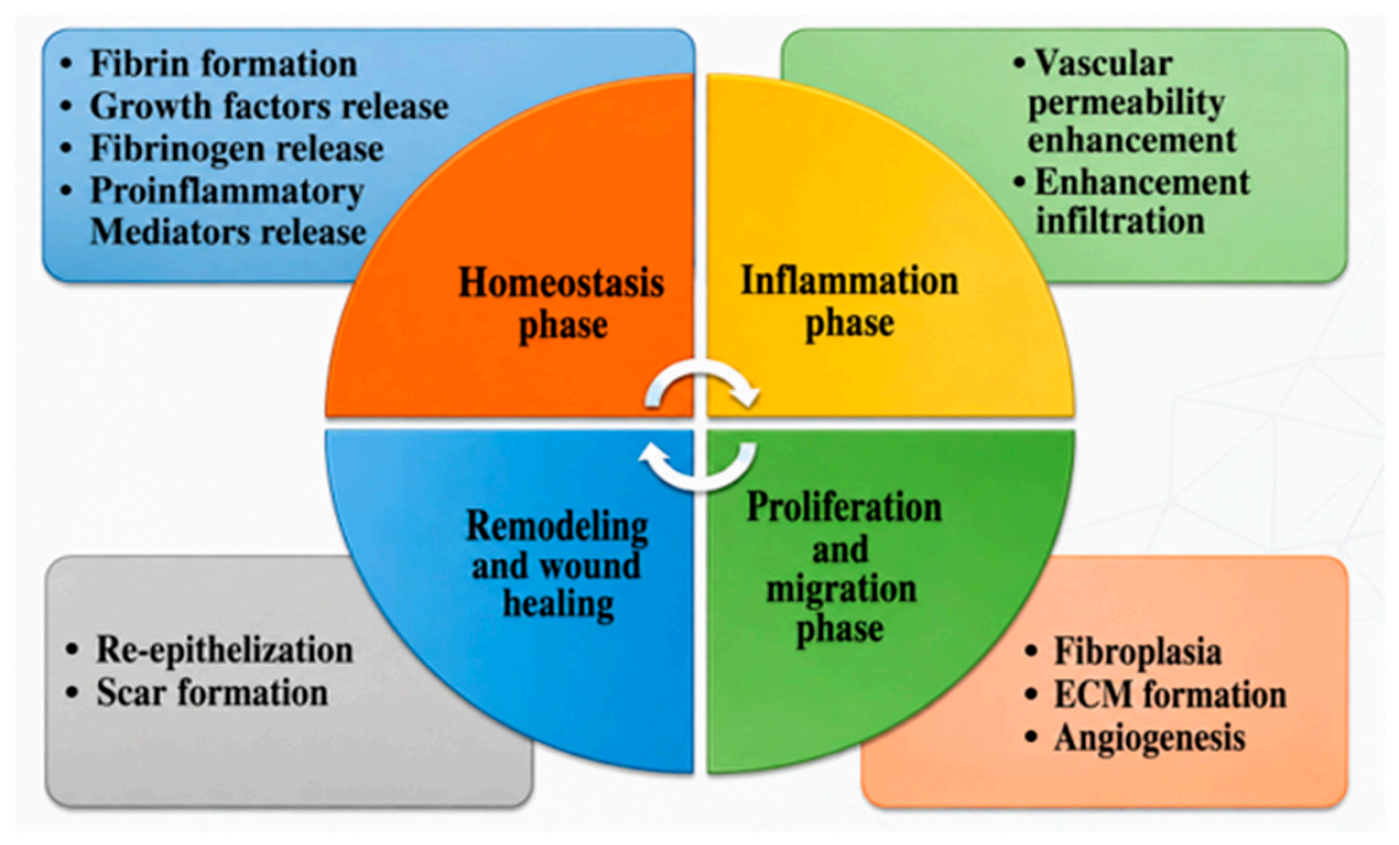
The four stages of normal wound healing [6].

The process of cutaneous wound healing is a highly coordinated, multi-stage biological cascade that is primarily driven by four pathological stages that overlap with one another. These phases are homeostasis, acute inflammation, cellular proliferation, and extracellular matrix (ECM) tissue remodeling, as illustrated in Figure 1 [1]. This specific timeline is responsible for coordinating the numerous cellular populations, soluble signaling mediators, and growth factors that are necessary for the effective execution of re-epithelialization and dermal fibroblast migration [2]. Such coordination takes place under normal physiological settings. However, significant physical trauma, burns that are full thickness, and persistent metabolic illnesses, most notably diabetes complications, usually disturb this delicate continuum, thereby locking the wound microenvironment in an inflammatory state that is prolonged and does not heal [3]. These chronic lesions provide an ever-increasing clinical conundrum, since they are characterized by localized tissue hypoxia, severe oxidative stress, and a high propensity to aggressive bacterial colonization [4]. To overcome these physiological obstacles, improved multi-functional therapeutic structures are required. These architectures should be able to simultaneously reduce hyper-inflammation, eliminate microbial pathogens, and speed up the regeneration of tissue [5].

Nanotechnology emerged as a new frontier in regenerative medicine and topical medication administration throughout the course of the previous decade. It has successfully circumvented the therapeutic ceilings that are faced by standard dressing arrangements [7]. Nanoparticles, which are defined as having dimensions that are less than 100 nanometers, have a wonderful surface-to-volume ratio. This ratio inherently increases the physicochemical interactions that nanoparticles have with biological membranes, which in turn improve intracellular translocation and retention within localized damage sites [8]. Among the many different types of inorganic nanovector, elemental selenium nanoparticles (SeNPs) have attracted a lot of attention from the academic community because of their excellent biocompatibility, decreased systemic toxicity in comparison to selenium ions, and distinct intrinsic biological activities [9]. Examples of inorganic nanovectors include gold nanoparticles (AuNPs), silver nanoparticles (AgNPs), silica nanoparticles, and metal oxide nanoparticles, which have been investigated for drug delivery, antimicrobial applications, and tissue engineering [10]. Selenium is a key trace element that supports functional selenoproteins like glutathione peroxidase and thioredoxin reductase, which scavenge free radicals and reduce cellular oxidative damage. SeNPs also disrupt bacterial cell membranes and prevent biofilm formation on exposed skin wounds by targeting major wound pathogens [11,12].

**Figure 2:**
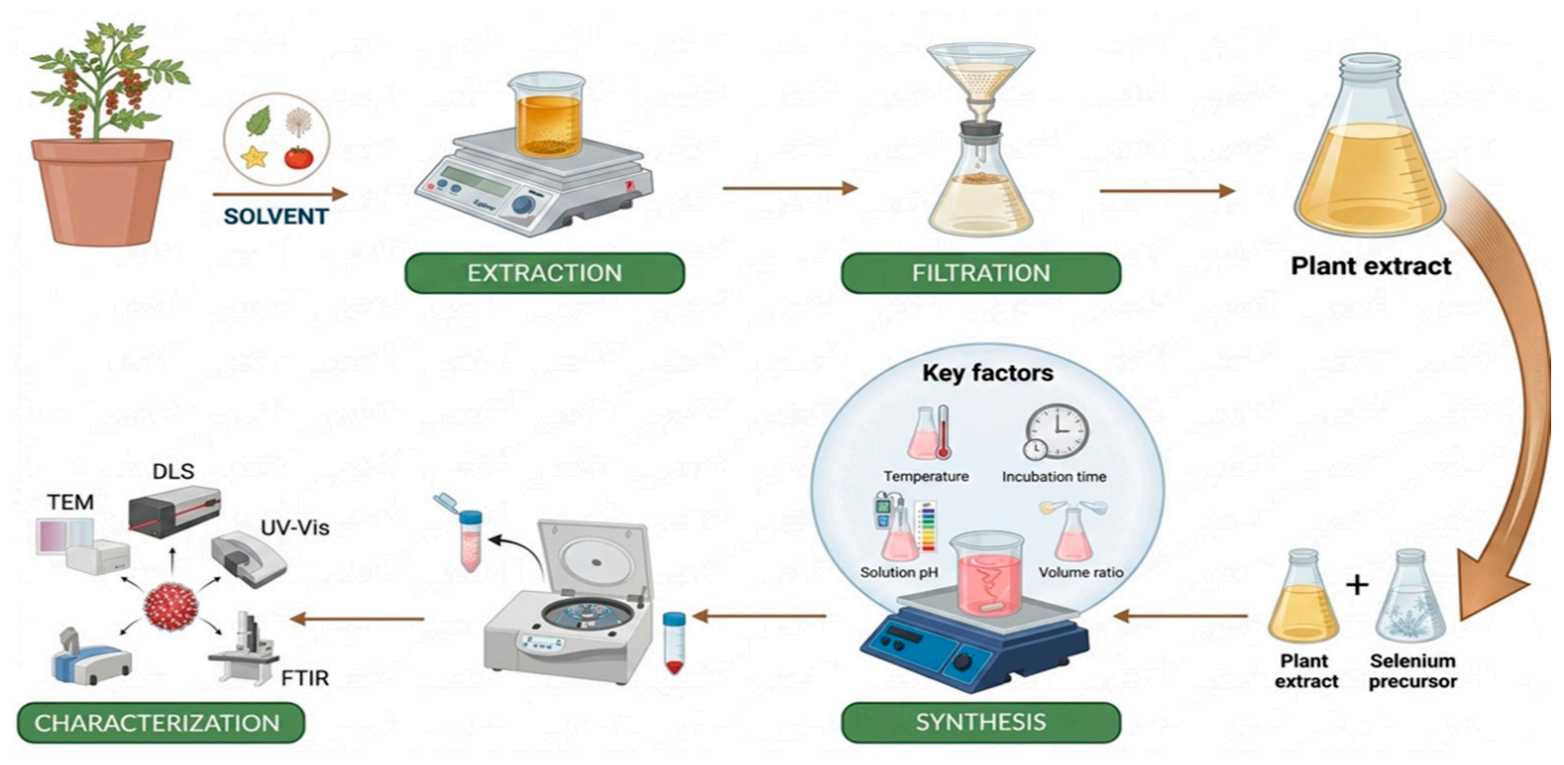
Synthesis of selenium nanoparticles from guava leaf extract [19].

Furthermore, in order to optimize the structural stability and safety profiles of SeNPs, which are otherwise prone to spontaneous agglomeration into inactive allotropic forms, novel green chemistry approaches that make use of natural plant extracts have been introduced [13]. Biosynthetic reduction, which makes use of natural precursors such as extracts from the leaves of the guava plant (Psidium guajava), provides a capping matrix that is both sustainable and beneficial to the environment [14]. The extract of guava leaf includes significant amounts of bioactive polyphenols, flavonoids, and terpenoids, all of which function as powerful natural reducing agents throughout the synthesis process, as illustrated in Figure 2 [15]. This environmentally friendly method avoids the need for potentially harmful chemical reducers while simultaneously endowing the surface of the nanoparticles that are produced with inherent antioxidant and secondary antibacterial capabilities—a synergistic combination [16,17]. It is possible to further improve the therapeutic activity of these biogenic nanocarriers by loading specific pharmacological substances that are complementary onto their surfaces [18].

**Figure 3:**
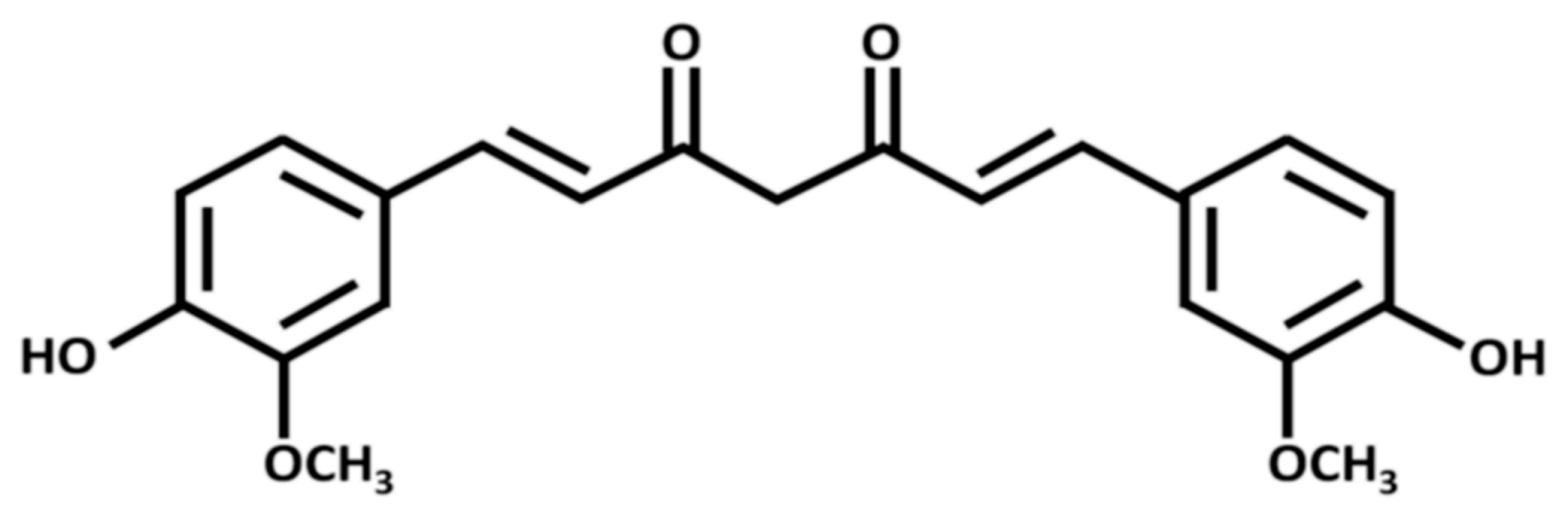
Chemical structure of curcumin [23].

Curcumin, a naturally occurring hydrophobic polyphenolic molecule that was discovered from Curcuma longa, is highly lauded for its remarkable ability to scavenge free radicals, as well as its anti-infective and tissue-regenerative characteristics [20]. By increasing the expression of transforming growth factor beta (TGF-β) and subsequently promoting a rapid shift from the acute inflammatory stage to the active fibroplasia and neovascularization stages, Curc has the ability to speed the process of wound contraction [21]. However, due to its low water solubility, rapid photodegradation, and restricted skin permeability, its therapeutic application through traditional topical methods continues to be in a state of severe limitation. The chemical structure of curcumin is shown in Figure 3 [22].

**Figure 4:**
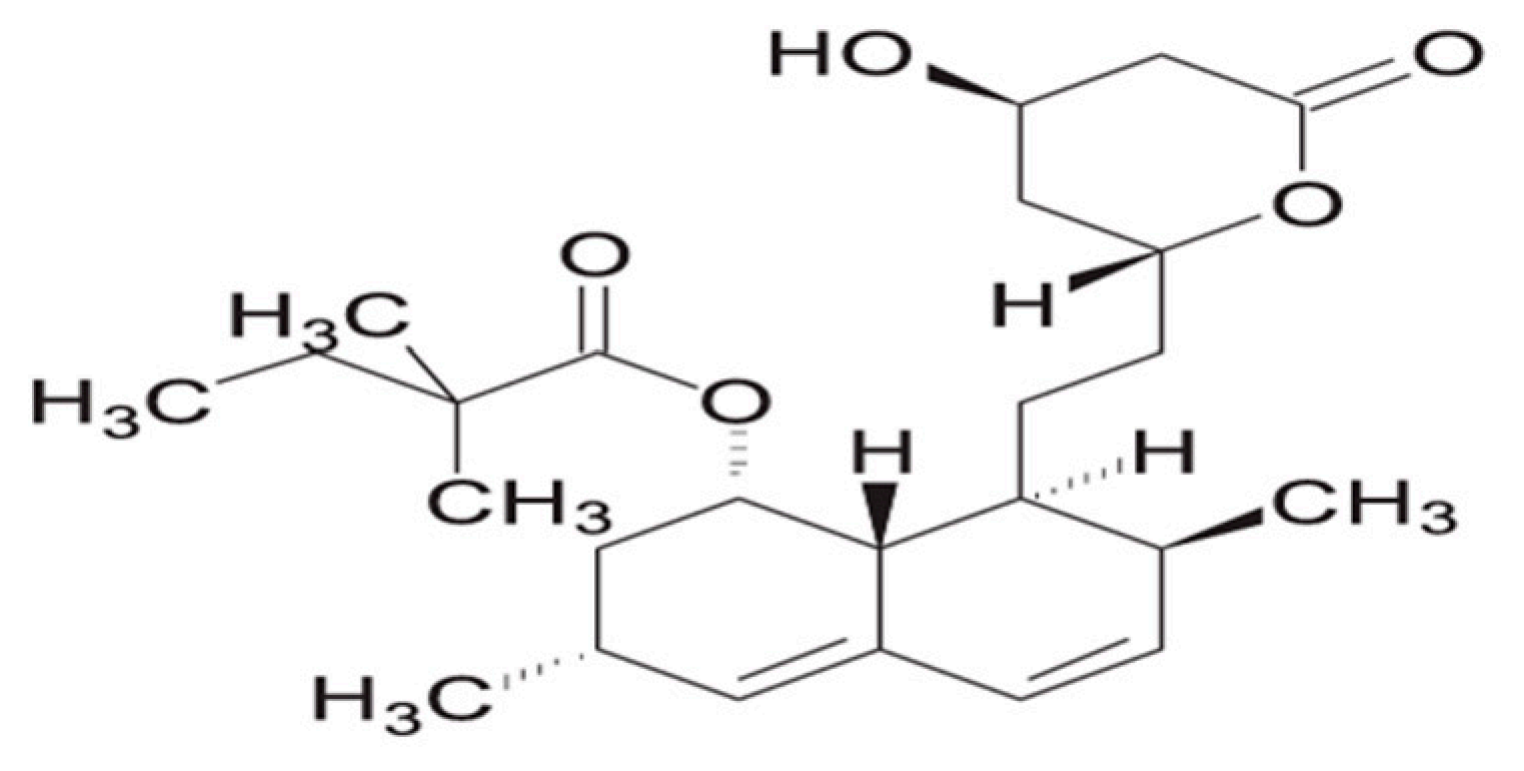
Chemical structure of simvastatin [28].

In a similar manner, SMV, which has traditionally been used as an anti-hyperlipidemic drug that is taken orally, has lately exhibited extraordinary pleiotropic potential when it is given topically [24]. To actively increase human keratinocyte motility and lymph angiogenesis, SMV selectively suppresses mevalonate pathways that are further downstream [25]. Additionally, SMV is a powerful antibacterial agent that inhibits the progression of local microorganisms, which makes it an advantageous addition to compound wound dressings [26]. However, similar to Curc, SMV has low water solubility, rapid local clearance, and variable bioavailability, which necessitates the utilization of a specialized carrier system to realize its full clinical potential. The chemical structure of simvastatin is shown in Figure 4 [27].

Through the inclusion of compound nanoparticles into responsive hydrogel matrices, an appropriate technical strategy can be achieved in order to create a prolonged and localized therapeutic effect on the skin [29,30]. Recent studies have further highlighted the potential of engineered nanostructured materials as localized therapeutic platforms, demonstrating that functionalized nanofibrous systems can provide favorable biocompatibility and therapeutic performance in preclinical models [30]. A moist wound-healing environment that resembles the natural extracellular matrix (ECM) is provided by topical hydrogel formulations. These formulations work by absorbing excess wound exudates while simultaneously facilitating the continuous and regulated release of encapsulated payloads over the dermal barrier [31]. Recent nanocarrier-based delivery strategies have similarly demonstrated the potential of nanoscale systems to improve drug delivery, sustain drug release, and enhance antibacterial activity against clinically relevant pathogens [32]. There is a paucity of literature that investigates the structural consolidation of both a natural phytochemical and a synthetic pleiotropic drug onto an intrinsically active, green-synthesized inorganic nanovector [33]. This is despite the fact that independent studies have provided specific details regarding the single application of either statins or herbal polyphenols in separate skin formulations [34]. The simultaneous and co-localized distribution of Curc and SMV through the utilization of an environmentally friendly SeNP matrix embedded inside a topical gel framework represents a novel paradigm in the field of cutaneous wound treatments that has not been addressed.

Although selenium nanoparticles, curcumin, simvastatin, and hydrogel-based topical systems have been investigated individually or in different combinations for therapeutic applications, the present study was designed to integrate these components into a single biogenic selenium nanoparticle-based nanocomposite hydrogel for the simultaneous topical delivery of curcumin and simvastatin. In particular, the study combined *Psidium guajava*-mediated synthesis of SeNPs, co-loading of the two poorly water-soluble drugs, incorporation of the optimized nanoassembly into 2% *w*/*v* Carbopol 934 hydrogel, and formulation optimization using a Box–Behnken response surface methodology. The resulting formulation was further evaluated using physicochemical characterization, in vitro drug-release studies, and an in vivo full-thickness excisional wound model. Thus, the novelty of the present work lies primarily in this integrated formulation and evaluation strategy rather than in the individual use of SeNPs, curcumin, simvastatin, or hydrogel as separate components.

This gap is addressed by the current study, which systematically constructed a biogenic, green-synthesized selenium nanoparticle platform utilizing Psidium guajava leaf extract. This platform is engineered was a dual-loaded cargo network for Curc and SMV [35]. Specifically, we discussed the environmentally biogenic synthesis and formulation optimization of the dual-drug-loaded SeNPs, which was then followed by the structural integration of these SeNPs into a specialized hydrogel matrix for topical application. Based on the findings of this inquiry, the major purpose was to conduct a comprehensive evaluation of the sustained in vitro drug release, multi-stage antioxidant capabilities, and broad-spectrum antibacterial performance of this unique nanogel [36]. In addition, we made use of an in vivo rat excision wound model to evaluate the effects of the combined formulation on wound closure, histological tissue reorganization, collagen deposition, and selected inflammatory and wound-healing biomarkers [37].

## 2. Materials and Methods

### 2.1. Materials

Sodium selenite was purchased from Adwia, El-Nasr, Pharm. Chem. Co., (Cairo, Egypt). Guava leaves were purchased from Egyptian farm, (Sohag, Egypt). Curcumin, simvastatin, Carbopol 934 and chitosan were purchased from Sigma Chemical Co., (St. Louis, MO, USA). Triethanolamine, ketamine hydrochloride, xylazine, buffered formalin, and hematoxylin and eosin (H&E) were purchased from Elnasr Pharmaceutical chemical Co., Cairo (Egypt). High-quality water employed to prepare solutions was obtained using a Milli-Q reagent water system, Continental Water Systems, (El Paso, TX, USA). All additional chemicals were of analytical quality, purchased from reputable vendors, and used without further purification.

### 2.2. Preparation and Optimization of Curcumin and Simvastatin Co-Loaded Selenium Nanoparticles Using Box–Behnken Experimental Design

#### 2.2.1. Preparation of Guava Leaf Extract

Guava leaves were collected from guava plant cultivated in Sohag, Egypt as a domestic fruit crop and washed several times with ultrapure water to remove any dust or foreign bodies then air-dried in an open atmosphere till complete dryness. Dried guava leaves (10 g) were boiled in (100 mL) of 60% ethanol for 2 min. Ultrapure water was added to make a dilution for the ethanolic extract with a ratio of (1:1), then filtrated through a filter paper until a pure extract was obtained, cooled, and stored at 4 °C [38].

#### 2.2.2. Synthesis of Selenium Nanoparticles

Sodium selenite salt (Na_2_SeO_3_) (25 mM) as a precursor was dissolved in 900 mL ultrapure water, then 100 mL of guava extract was added dropwise to the selenite solution. Incubate the mixture with stirring using a magnetic stirrer (Dubuque, IA, USA) at 800 rpm and a temperature of 60 °C for 24 h, then centrifugation at 13,280 rpm for 20 min, washing several times with ultrapure water to remove unreacted selenite, and air-dried [38,39].

#### 2.2.3. Co-Loading of Curcumin and Simvastatin onto Selenium Nanoparticles

Curcumin and simvastatin were dispersed in ethanolic solution 30% (*v*/*v*) under ultrasonic conditions to ensure complete dispersion. Separately, SeNPs dispersed in distilled water were mixed with a chitosan–acetic acid solution under vigorous stirring to obtain a stabilized SeNP suspension. Subsequently, the ethanolic solution containing Curc and SMV were added dropwise to the stabilized SeNP–chitosan dispersion and continuously stirred for 24 h at room temperature to facilitate drug adsorption onto the SeNP surface. The resulting suspension was then subjected to repeated centrifugation at 9000 rpm for 5 min until a colorless supernatant was obtained. Finally, an SeNP pellet was collected and dried overnight in a drying furnace (Lilienthal, Germany) at 40 °C to obtain the selected Curc–SeNP–SMV formulation [40,41].

#### 2.2.4. Preparation of Selected Curc–SeNP–SMV Carbopol 934 Gel

A prepared 2% (*w*/*v*) Carbopol 934 gel was used to load the selected Curc–SeNP–SMV into gel. The blank Carbopol 934 gel was prepared by dissolving the high-molecular-weight Carbopol 934 in water while continuously stirring. The resulting semi-solid dispersions were carefully handled and triturated to avoid air bubble entrapment. Triethanolamine (2%, *w*/*v*) was added to neutralize the dispersion and produce the thick, translucent gel for the gel base. A homogeneous gel was produced after 1 h of continuous stirring [42].

### 2.3. Characterization Studies

#### 2.3.1. Solubility Study of Curcumin and Simvastatin in Ethanol

A solubility study was conducted to evaluate the solubility of Curc and SMV in various ethanol concentrations. Ethanol (2 mL; 10%, 30%, and 50% *v*/*v*) was added to 20 mg—excess amount—of each drug individually. For 48 h, the samples were shaken in a water bath at 37 °C to achieve equilibrium. After equilibration, a 0.45 µm membrane filter was used to filter the samples after they had been centrifuged at 4000 rpm for 15 min. Using UV-visible spectrophotometry (UK), the amount of Curc and SMV in the filtrate were measured at λ_max_ 425 nm and 242 nm, respectively [43,44,45].

#### 2.3.2. Docking Study of Curcumin and Simvastatin

Molecular docking studies of Curc and SMV were performed to predict and evaluate the binding abilities of the targeted ligands with COX-1 and COX-2 enzymes. All the molecular modeling studies and docking simulation studies were carried out using Molecular Operating Environment (MOE^®^) 2014.01, Chemical Computing Group (CCG) Inc., Montreal, Canada. All the interaction energies and different calculations were automatically calculated. X-ray crystal structures of COX-1 and COX-2 enzymes were obtained from the Protein Data Bank (PDB) (www.rscb.org). COX-1 (PDB entry: 3N8Y) and COX-2 (PDB ID: 1CX2) below 2.00 Å resolution. The enzyme structures were prepared for docking studies by: (a) removal cofactors from the protein that are not relevant to the enzyme binding, (b) adding hydrogen atoms to the enzyme, and (c) fixing the potential of the enzyme atoms. The obtained poses were studied, and those that showed the best ligand–enzyme interactions were selected and stored for energy calculations [46].

#### 2.3.3. X-Ray Diffraction of Synthesized SeNPs

The XRD pattern of the synthesized SeNPs was examined using a generator (PW1930), a goniometer (PW1820), and a Diano X-ray diffractometer (Philips, The Netherlands), with a CuK radiation source (λ = 0.15418 nm) triggered at 45 kV [47].

#### 2.3.4. Box Behnken Design

A Box–Behnken design (BBD) was employed to evaluate the effects of the two independent formulation variables, namely the selenium nanoparticle-to-drug ratio (SeNPs, C_1_) and ethanol concentration (Eth Conc., C_2_), on the selected dependent responses: entrapment efficiency (E_1_), cumulative percentage drug release after 2 h (E_2_; %Rel_2_ h), and cumulative percentage drug release after 8 h (E_3_; %Rel_8_ h). Each independent variable was investigated at three levels, resulting in nine experimental formulations. Curcumin and simvastatin were incorporated at a fixed 1:1 (*w*/*w*) Curc ratio, while the total drug amount was varied relative to the amount of SeNPs according to SeNP ratios of 0.5:1, 0.75:1, and 1:1. Ethanol concentration was investigated at 30%, 40%, and 50% (*v*/*v*), as shown in Table 1.

The experimental data were analyzed using response surface methodology (RSM), and multiple regression analysis was performed to establish the mathematical relationships between the independent variables and the measured responses. Initially, a first-order model was considered (Equation (1)):

(1) **E** = β_0_+ β_1_C_1_+ β_2_C_2_

where *E* represents the predicted response, β_0_ is the intercept, and β_1_ and β_2_ are the linear regression coefficients corresponding to the effects of C_1_ and C_2_, respectively.

To account for potential interaction and curvature effects, a second-order polynomial model was subsequently fitted to the experimental data (Equation (2)):

(2) **E** = β_0 +_ β_1_ C_1_ + β_2_ C_2_ + β_12_ C_1_ C_2_ + β_11_ C_1_^2^ + β_22_ C_2_^2^

where E represents the predicted response; β_0_ is the intercept; β_1_ and β_2_ are the linear regression coefficients associated with C_1_ and C_2_, respectively; β_12_ is the interaction coefficient representing the combined effect of C_1_ and C_2_; and β_11_ and β_22_ are the quadratic coefficients associated with C_1_^2^ and C_2_^2^, respectively. The magnitude and sign of each estimated regression coefficient indicate the relative magnitude and direction of the corresponding effect on the response. Positive coefficients indicate a direct relationship between the corresponding factor and the response, whereas negative coefficients indicate an inverse relationship within the investigated experimental domain [48,49,50].

##### Determination of Entrapment Efficiency

The entrapment efficiency (%EE) of Curc and SMV within the SeNPs was determined by centrifuging the Curc–SeNP–SMV formulations at 9000 rpm for 30 min to separate the unentrapped drug from the nanoparticle-associated fraction. The supernatant was collected, and the concentrations of free Curc and SMV were determined using a UV-visible spectrophotometer (Shimadzu, Kyoto, Japan) at 425 and 242 nm, respectively. The entrapment efficiency was calculated according to (Equation (3)):

(3) $$\mathrm{EE}\left(\%\right)=\frac{T_{D}-F_{D}}{T_{D}}\times 100$$

where *T*D is the total amount of drug initially added to the formulation and *F*D is the amount of unentrapped drug detected in the supernatant [51].

##### In Vitro Drug-Release Study

The in vitro release of Curc and SMV from the selected and experimental Curc–SeNP–SMV formulations was evaluated using a dialysis membrane method. The formulations were placed in dialysis membranes and immersed in 30% (*v*/*v*) ethanol–phosphate-buffered saline (PBS; pH 7.4) maintained at 37 °C under continuous stirring. At predetermined time points, 2 h and 8 h, 1 mL aliquots of the release medium were withdrawn and immediately replaced with an equal volume of fresh medium to maintain sink conditions. The concentrations of Curc and SMV in the collected samples were determined spectrophotometrically at 425 and 242 nm, respectively. The cumulative percentage of drug released was calculated and plotted as a function of time to generate the corresponding release profiles [52].

##### Statistical Analysis and Model Evaluation

The fitted polynomial models were evaluated by analysis of variance (ANOVA) to determine the statistical significance of the regression models and individual model terms. The adequacy of the models was assessed based on the significance of the model *p*-value and other relevant statistical parameters. A probability value of *p* < 0.05 was considered indicative of statistical significance. The resulting regression equations were subsequently used to characterize the individual, interaction, and quadratic effects of SeNP:D and ethanol concentration on %EE, %Rel_2_ h, and %Rel_8_ h, and to identify the formulation conditions providing the desired combination of responses [53,54].

#### 2.3.5. FT-IR Spectroscopy

Fourier-transform infrared spectroscopy (FT-IR) analysis was carried out for the selected Curc–SeNP–SMV, pure SeNPs, Curc, SMV, and chitosan using a Shimadzu Fourier-transform infrared spectrophotometer (FT-IR-8400S, Shimadzu, Japan) to ensure that there were no intermolecular interactions. Samples were compressed using a hydraulic press at a pressure of 6 tons/cm^2^, and scanned between 4000 and 500 cm^−1^.

#### 2.3.6. Scanning Electron Microscopy

Scanning electron microscopy (SEM) was used to analyze the topographical features and surface details of both the synthesized SeNPs and the selected Curc–SeNP–SMV formulations. Visual inspection and morphological characterization of the samples were performed using a Zeiss scanning electron microscope (Carl Zeiss AG, Oberkochen, Germany).

#### 2.3.7. Particle Size Analyses of Curc–SeNP–SMV

Using photon correlation spectroscopy and the particle size analyzer Dynamic Light Scattering (DLS) (Malvern Instruments Ltd., Worcestershire, UK), the selected drug-loaded SeNPs was examined for their size and size distribution in terms of average volume diameters and polydispersity index (PDI). Samples were analyzed in triplicate at a fixed angle of 173° at 25 °C.

#### 2.3.8. Physical Appearance of Curc–SeNP–SMV Gel

The color, homogeneity, consistency, and existence of any aggregates or phase separation were all visually examined in the prepared Carbopol 934 hydrogel.

#### 2.3.9. pH Determination of Curc–SeNP–SMV Gel

A digital pH meter, (Jenway Ltd., Dunmow, UK) that had been calibrated was used to measure the hydrogel formulation’s pH. Before being measured, 1 g of the Carbopol 934 hydrogel was dissolved in 10 milliliters of distilled water and given 30 min to equilibrate [19].

#### 2.3.10. Viscosity Study of Curc–SeNP–SMV Gel

A Bohlin rotational viscometer (Bohlin Instruments Ltd., Cirencester, UK) was used to measure the selected Curc–SeNP–SMV gel’s viscosity at room temperature (25 °C) without dilution [55].

#### 2.3.11. In Vitro Drug Release of Curc–SeNP–SMV Gel

Dialysis bags containing 1 g of Curc–SeNP–SMV gel were suspended in 30% (*v*/*v*) ethanol–phosphate buffer release medium with a pH of 7.4 and kept at 37 ± 0.5 °C. At predetermined intervals, 1 mL aliquots were removed and replaced with the same volume of release media. Curc and SMV amounts in the aliquots were measured using UV spectroscopy at λmax 425 nm and 242 nm, respectively [53].

#### 2.3.12. In Vivo Assessment Study

##### Experimental Protocol

Male and female inbred Wistar rats with body weights ranging from 180 to 200 g were sourced from the central animal facility at the Faculty of Medicine, Sohag University, Sohag, Egypt. Before and throughout the duration of the experiments, the animals were housed in the departmental vivarium for one week under controlled environmental conditions, including a temperature of 26 ± 2 °C, relative humidity between 44% and 56%, and a light–dark photoperiod of 10 and 14 h, respectively. The rats were always given free access to water and a standard commercial pellet diet. All experimental procedures were conducted in strict adherence to the *Principles of Laboratory Animal Care* (NIH publication 82-23, revised 1985), and formal ethical approval was granted by the Institutional Animal Ethics Committee of the Faculty of Medicine, Sohag University, before initiating any experimental work [(12-1-02/2026-01)].

A preliminary dose-finding study was carried out to evaluate the wound-healing potential of varying concentrations of Curc and SMV, individually and in combination, when incorporated into a gel base. An excision wound model in rats served as the experimental platform. Gel preparations containing either 1% or 2% of each active agent were applied topically once per day for up to ten days, or until complete re-epithelialization was observed. All formulations were prepared according to established pharmaceutical methods to ensure homogeneity within a neutral gel carrier. The primary objective of this pilot phase was to identify the optimal therapeutic concentration for each compound by monitoring wound contraction rates and the time required for complete epithelialization. Analysis of the results revealed that the 2% formulations consistently produced better healing outcomes than their 1% counterparts, with faster wound closure and more evident tissue regeneration across all evaluated parameters. Based on these preliminary findings, the 2% concentration was adopted for subsequent experimental stages. In all follow-up experiments, 1 g of the respective gel was applied once daily to the wound surface using a sterile spatula, beginning four hours after wound creation and continuing for 14 consecutive days. The control animals received only the gel base without any active ingredient. Wound healing was evaluated by measuring wound area, time to complete epithelialization, biochemical markers in tissue samples, and histological outcomes.

##### Study Design and Animal Grouping

A total of 48 Wistar rats were randomly divided into eight equal groups (n = 6) per group to assess the combined topical wound-healing efficacy of the formulated hydrogels over a span of 14 consecutive days as outlined below.

Group I (intact control): Healthy subjects that received neither surgical procedures nor topical therapies, acting as the definitive baseline group.

Group II (sham-operated control): Subjects that experienced the complete surgical excision process to create wounds but did not receive any active drug or placebo intervention.

Group III (placebo control/blank gel): Injured animals received daily treatment with a topical application of the inert, drug-free hydrogel base matrix.

Group IV (pure SeNPs gel): Wounded animals were treated daily with biogenic SeNPs incorporated into the gel at a concentration of 2% *w*/*w*, without curcumin or simvastatin.

Group V (Curc–SeNP gel): Wounded animals received daily topical treatment with Carbopol 934 hydrogel containing Curc -loaded SeNPs, with Curc administered at the selected therapeutic concentration of 2% *w*/*w*.

Group VI (SMV–SeNP gel): Wounded animals received daily topical treatment with Carbopol 934 hydrogel containing SMV-loaded SeNPs, with SMV administered at the selected therapeutic concentration of 2% *w*/*w*.

Group VII (full-dose combination gel/Curc–SeNP–SMV): Wounded animals received daily topical treatment with Carbopol 934 hydrogel containing the dual-loaded Curc–SeNP–SMV nanoassembly, with Curc and SMV administered at the full therapeutic concentration of 2% *w*/*w* for each active ingredient.

Group VIII (half-dose combination gel/Curc–SeNP–SMV): Wounded animals received daily topical treatment with Carbopol 934 hydrogel containing the dual-loaded Curc–SeNP–SMV nanoassembly, with Curc and SMV administered at half of the full therapeutic concentration, corresponding to 1% *w*/*w* for each active ingredient.

All gel formulations were applied daily starting from day 1 through day 14 of the study.

Appropriate control groups were included in the in vivo wound-healing study to facilitate interpretation of the effects of the different formulation components. The blank Carbopol 934 hydrogel group was used to assess the effect of the hydrogel vehicle, while the unloaded SeNP hydrogel group was included to evaluate the contribution of the selenium nanoparticle component in the absence of curcumin and simvastatin. The Curc–SeNP and SMV–SeNP groups allowed evaluation of the effects of the individual drug-loaded SeNP formulations, whereas the half-dose and full-dose Curc–SMV co-loaded SeNP formulations were used to evaluate the combined formulation at two administered dose levels. These comparisons enabled assessment of the wound-healing responses associated with the different formulation components under the experimental conditions of the study.

##### Excisional Wound Model

All animals were anesthetized before the surgical procedure by intraperitoneal administration of ketamine (80 mg/kg) and xylazine (10 mg/kg). The dorsal hair was carefully shaved, and the surgical site was disinfected with 70% ethanol under aseptic conditions. A standardized full-thickness circular excisional wound measuring 1.5 cm in diameter was created on the dorsal skin of each rat using a sterile surgical blade/biopsy punch. The animals were then randomly allocated into the respective treatment groups and treated with the assigned gel formulation by topical application once daily. The wounds were left uncovered throughout the experimental period without the use of additional dressings. Wound healing was monitored at predetermined time points, and wound tissue samples were collected at the end of the study following euthanasia for subsequent histopathological examination [56,57].

##### Wound-Healing Assessment

The assigned gel formulations were applied topically to the wounds once daily for 14 consecutive days, ensuring uniform coverage of the entire wound surface. The progression of wound healing was evaluated by photographing the wounds on days 0, 3, 7, and 14 using a digital camera under standardized conditions. To ensure reliable comparison among animals and time points, photographs were captured at a fixed distance and under consistent lighting conditions.

The wound area was determined from the digital images using ImageJ software (version 1.53, National Institutes of Health, Bethesda, MD, USA). The percentage of wound contraction was calculated according to Equation (4):

(4) Wound Contraction (%) = (A0 − At)/A0 × 100

where A_0_ is the initial wound area measured immediately after wound creation (day 0) and A_t_ is the wound area measured at the corresponding time point. Following completion of the experimental period, the animals were euthanized according to the approved experimental protocol, and representative wound tissue together with adjacent normal skin was excised and fixed in 10% neutral buffered formalin for subsequent histopathological evaluation [58,59].

##### Histopathology

Skin biopsy specimens were placed in 10% buffered formalin for a 24 h fixation period, after which they were processed and embedded in paraffin wax using standard protocols. Tissue sections of approximately 5 µm thickness were cut using a Leica RM 2145 microtome (Leica Microsystems, Nussloch, Germany) and stained with hematoxylin and eosin (H&E). Sections were examined under 40× magnification to assess epidermal thickness. To define the boundaries of necrotic tissue and the total flap area, digital images were captured at 20× magnification using an Olympus BX51 light microscope equipped with an Olympus C-5050 digital camera (Olympus Corporation, Tokyo, Japan), and analysis was performed with Image Pro Express software (Version 4.5.1.3, Media Cybernetics Inc., USA, 2002) [58,60].

##### Biochemical Study

At the conclusion of the experiment, skin tissue samples were collected, rinsed with normal saline, and then trimmed and mechanically homogenized in ice-cold phosphate-buffered saline (PBS) at a volume four times that of the tissue. The homogenates were then centrifuged at 12,014× *g* for 5 min, and the resulting supernatants were aliquoted and stored at −80 °C until further analysis. Levels of IL-1β, MMP-9, IL-10, and COX-2 were quantified using commercially available ELISA colorimetric kits from BioVision, Inc. (Milpitas, CA, USA), following the manufacturer’s instructions precisely [61].

### 2.4. Statistical Analysis

All statistical analyses were carried out using GraphPad Prism software (version 7.0, GraphPad Software, CA, USA). Results are reported as means ± standard deviation (SD). Differences among groups were assessed using one-way analysis of variance (ANOVA), followed by the Tukey–Kramer post hoc test for pairwise multiple comparisons. A *p*-value less than 0.05 was considered indicative of statistical significance.

## 3. Results and Discussion

### 3.1. Preparation of Guava Leaf Extract

The hydroethanolic extraction of Psidium guajava foliage collected from Sohag, Egypt produced a transparent, dark greenish-brown concentrated solution, as shown in Figure S1A (see Figure S1 in the Supplementary Materials). Desiccating the leaves at room temperature successfully removed moisture without causing thermal damage to sensitive bioactive constituents. Heating the desiccated biomass (10 g) in 60% ethanol for a short period (2 min) enabled fast cell wall breakdown, promoting effective transfer of phytochemicals into the solvent. The ensuing 1:1 dilution with ultrapure water effectively stabilized the extract, inhibiting the precipitation of lipophilic constituents while preserving a uniform phase. The filtration process produced a pristine, particulate-free ethanolic extract, as shown in Figure S1B (see Figure S1 in the Supplementary Materials). Preservation at 4 °C maintained the structural integrity of the extract, exhibiting no visible microbial proliferation, sediment formation, or color changes over time, signifying substantial physical and chemical stability [62].

### 3.2. Synthesis of Selenium (SeNPs)

The biological reduction of sodium selenite to synthesize SeNPs was conducted using Psidium guajava leaf extract at 60 °C and 800 rpm to give a crimson solution. The phytoconstituents of the extract reduced Se^4+^ to elemental Se^0^ and acted as capping agents, enhancing stability and reducing aggregation for potential biological applications, as shown in Figure S1C (see Figure S1 in the Supplementary Materials) [63].

### 3.3. Co-Loading of Curcumin and Simvastatin onto Selenium Nanoparticles

The synthesized SeNPs were successfully applied for simultaneous loading of the hydrophobic pharmaceuticals Curc and SMV using a chitosan-facilitated surface adsorption method. The initial distribution of Curc and SMV in ethanol by ultrasonic treatment was a totally homogeneous clear solution without any visible drug sediment. The combination of SeNPs with cationic polymer chitosan in acetic acid medium yields an extraordinarily stable and homogeneous colloidal suspension. The gradual addition and stirring at room temperature during the 24 h period caused a clear physical change as the pharmaceuticals were incorporated into the polymeric shell of the nanoparticles. The purification process through sequential centrifugation at 9000 rpm for 5 min yielded a transparent, colorless supernatant, visually validating the elevated encapsulation and adsorption efficacy of both therapeutic agents onto the nanocarrier matrix. The concluding thermal drying procedure at 40 °C in a drying furnace produced a refined, thoroughly dehydrated, and readily redispersible powder of the selected Curc–SeNP–SMV formulation [64,65].

### 3.4. Preparation of Selected Curc–SeNP–SMV Carbopol 934 Gel

The integration of the refined Curc–SeNP–SMV nanoformulation into a 2% (*w*/*v*) Carbopol 934 polymeric matrix resulted in a uniform, smooth, and visually appealing medicated hydrogel. The preliminary breakdown of Carbopol 934 in water yielded a low-viscosity, acidic semi-solid suspension. Meticulous manipulation and regulated trituration successfully averted the inclusion of air bubbles, guaranteeing structural consistency. The incorporation of 2% (*w*/*v*) triethanolamine (TEA) resulted in an instantaneous and noticeable physical change: the acidic dispersion was neutralized, swiftly expanding into a dense, viscous, and transparent gel matrix. After one hour of uninterrupted stirring post the addition of Curc–SeNP–SMV nanoparticles, the formulation exhibited remarkable physical compatibility, yielding a uniformly distributed yellowish-orange nanocomposite gel (due to Curc’s natural hue) devoid of any phase separation, particle clumping, or grittiness, as shown in Figure S2 (see Figure S2 in the Supplementary Materials) [66,67].

### 3.5. Characterization Studies

#### 3.5.1. Solubility Study

**Figure 5:**
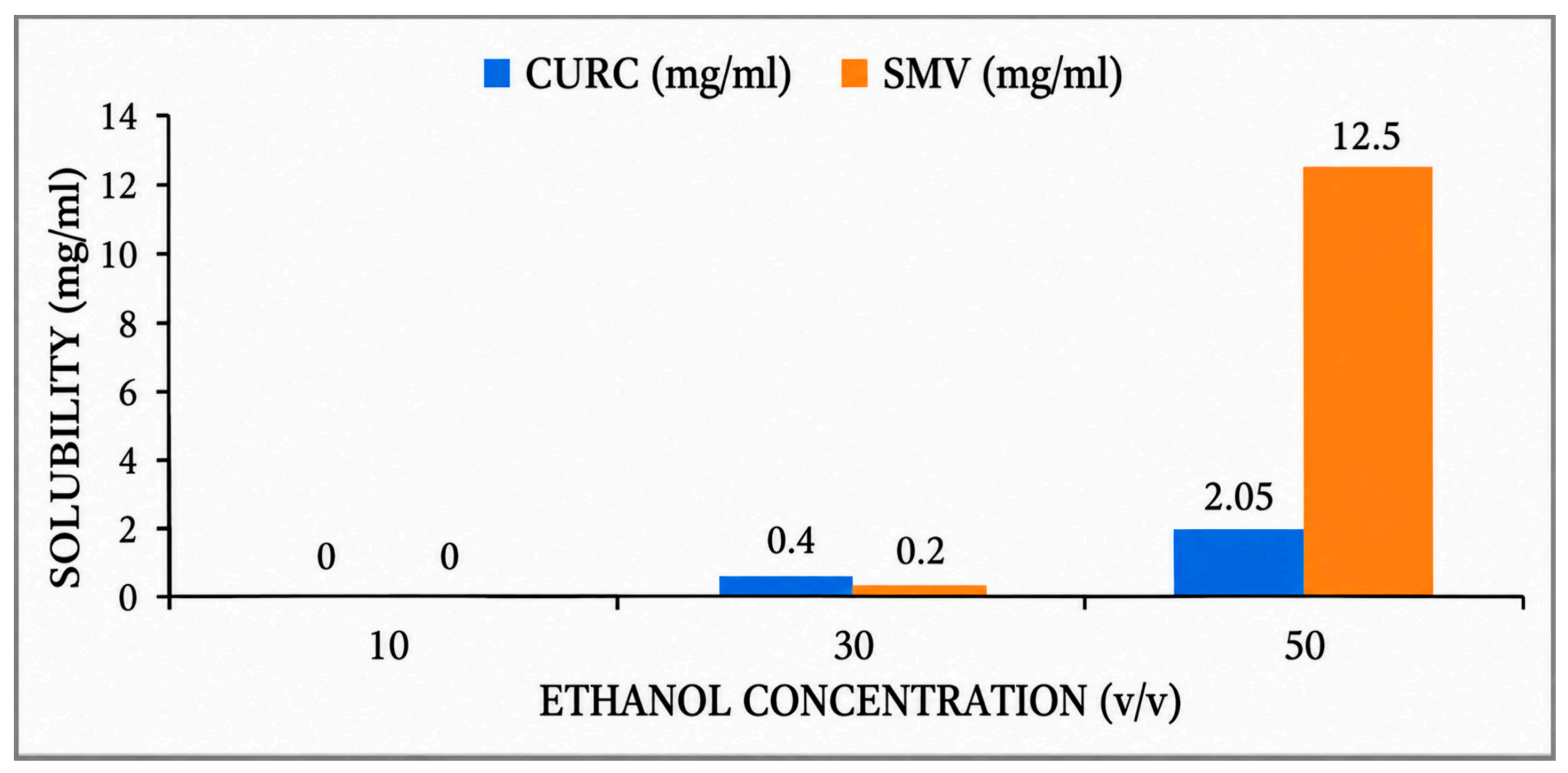
Effect of ethanol concentration (10, 30, and 50% *v*/*v*) on the solubility of Curc and SMV in ethanol solutions.

Employing UV-visible spectrophotometry, Cam Spec (UK) assessed the solubility of Curc and SMV in different concentrations of aqueous ethanolic solutions (10%, 30%, and 50% *v*/*v*) at λmax 425 nm and 242 nm. Both substances exhibited remarkably low solubility in 10% *v*/*v* ethanol and failed to completely dissolve due to their hydrophobic nature. Ethanol exhibits a co-solvent action that diminishes the polarity of the solvent system and enhances the solubilization of hydrophobic pharmaceuticals. The solubility of Curc and SMV was augmented with the elevation of ethanol concentration to 30% *v*/*v*. Although 50% *v*/*v* ethanol exhibited the greatest solubility, this concentration is unsuitable for SeNPs due to the possible impact of elevated organic solvent levels on nanoparticle stability. The solubility study demonstrated that both Curc and SMV exhibited limited apparent solubility in the lower ethanol concentration tested, whereas their apparent solubility increased with increasing ethanol concentration. Although 50% *v*/*v* ethanol provided the highest apparent solubilization, 30% *v*/*v* ethanol was selected as a practical compromise between improving the apparent solubilization of the poorly water-soluble drugs and maintaining suitable conditions for nanoparticle formulation. Accordingly, the formulation strategy employed an ethanolic cosolvent system together with SeNP-based drug association to facilitate drug dispersion within the formulation. Consequently, a 30% *v*/*v* ethanol solution was selected as the optimal solvent, as it preserved the necessary conditions for nanoparticle stability while ensuring sufficient drug solubility [44,45,68]. Based on this outcome, the volume of the release medium will be modified in our research to maintain sink conditions for both pharmaceuticals, as illustrated in Figure 5.

#### 3.5.2. Docking (In Silico Study)

##### Molecular Modeling Study

To achieve accurate validation of the molecular basis underlying the proposed biological activities, molecular docking analyses were conducted for Curc and SMV in the endeavor to expand the arsenal of targeted treatments for wound healing and inflammation. The evaluated docked complexes included Curc and SMV within the active site of COX-1 and COX-2 enzymes attaining the highest inhibition activity, which play distinct but crucial roles in wound healing compared to the reference inhibitor drug diclofenac [69].

Molecular modeling involves the generation, manipulation and representation of 3D structures of molecules and their physicochemical properties. Recently, molecular modeling has played an essential role in the drug design process. It depends on using computerized techniques to predict molecular and biological properties. Molecular modeling is easy to perform with currently available software, but getting the right model and proper interpretation are the main difficulties.

Currently, the major modeling strategies are used for the conception of new drugs.

In the direct approach, the three-dimensional features of the known receptor site are determined from X-ray crystallography to design a lead molecule. Consequently, the receptor site geometry is known; the problem is to find a molecule that satisfies some geometric constraints and is also a good chemical match. After finding good candidates according to these criteria, a docking step with energy minimization can be used to predict binding strength [70].

Docking studies were carried out to elucidate the binding mode Curc and SMV with the target enzymes COX-1 (PDB entry: 3N8Y) and COX-2 (PDB ID: 1CX2) [71]. The docking calculations were run and the obtained poses studied using Molecular Operating Environment (MOE) software, Chemical Computing Group (CCG) Inc., Montreal, Canada, provided the Molecular Operating Environment (MOE^®^) version 2014.01, Chemical Computing Group Inc., Montreal, QC, Canada, 2014. Every calculation, including the interaction energies, was done automatically. X-ray crystal structures were acquired below 2.00 Å resolution from the Protein Data Bank (www.rscb.org) using the protein as a macromolecule and medications as ligands [72].

The structures were prepared for docking studies by cofactors from the protein that are not relevant to the enzyme binding, adding hydrogen atoms to the system, and fixing the potential of the enzyme atoms.

The docking findings provide a computational perspective that may help contextualize the observed anti-inflammatory and wound-healing responses. Curc and SMV showed favorable predicted interactions with the active sites of COX-1 and COX-2 under the applied docking conditions. These findings provide a possible structural rationale for considering Curc and SMV as contributors to modulation of inflammatory pathways that may influence the wound-healing environment. However, docking results represent predictive molecular-level evidence and do not establish enzymatic inhibition or a direct causal relationship with the in vivo wound-healing effects observed in the present study. Experimental biochemical and tissue-localization studies would be required to confirm these proposed mechanisms.

**Figure 6:**
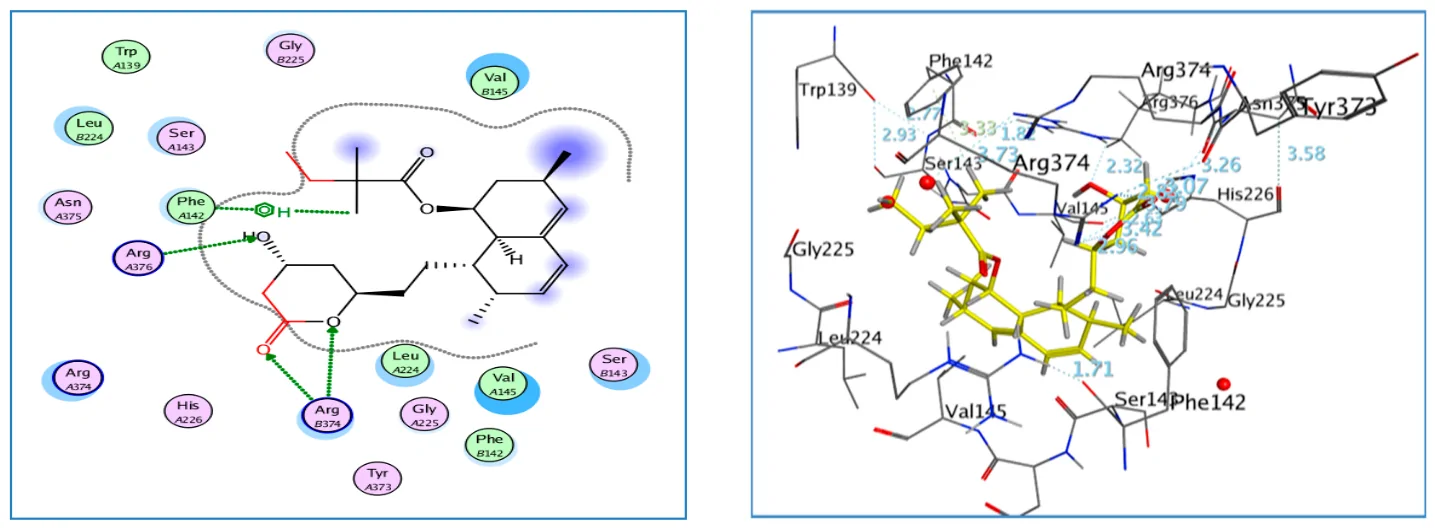
Molecular docking of SMV into the COX-1 active site: 2D diagram and 3D diagram representations showing its binding interactions with the amino acid residues.

**Figure 7:**
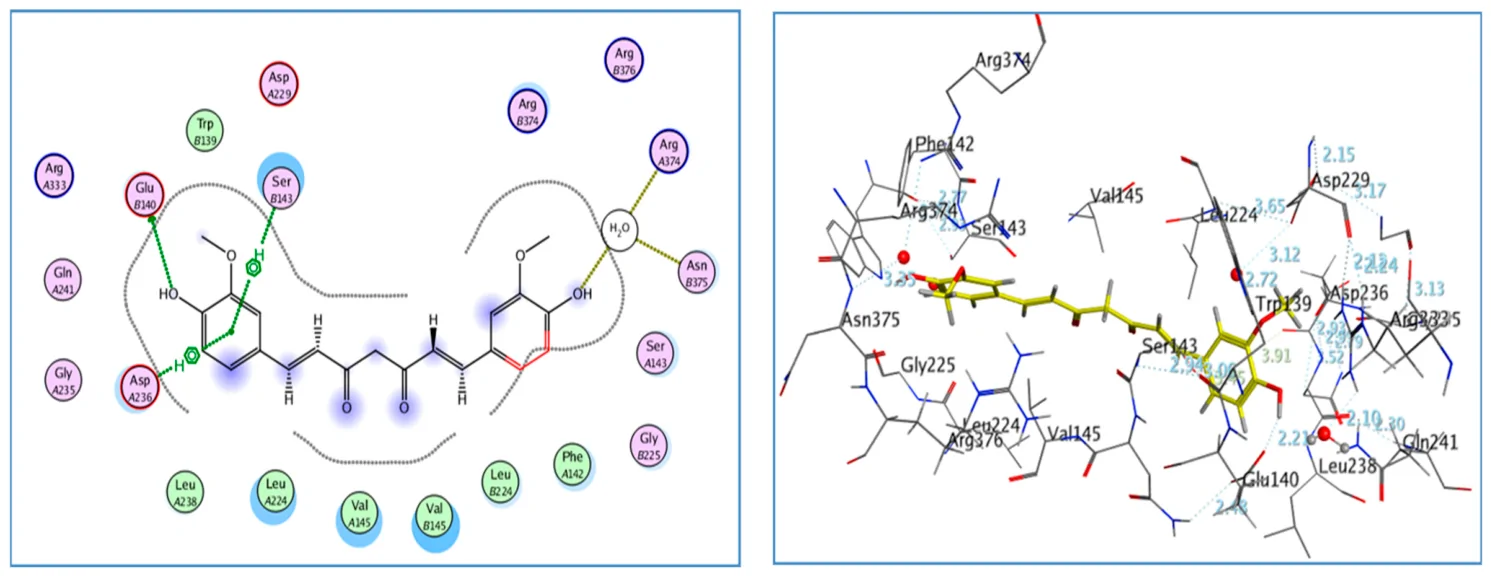
Molecular docking of Curc into the COX-1 active site: 2D diagram and 3D diagram representations showing its binding interactions with the amino acid residues.

**Figure 8:**
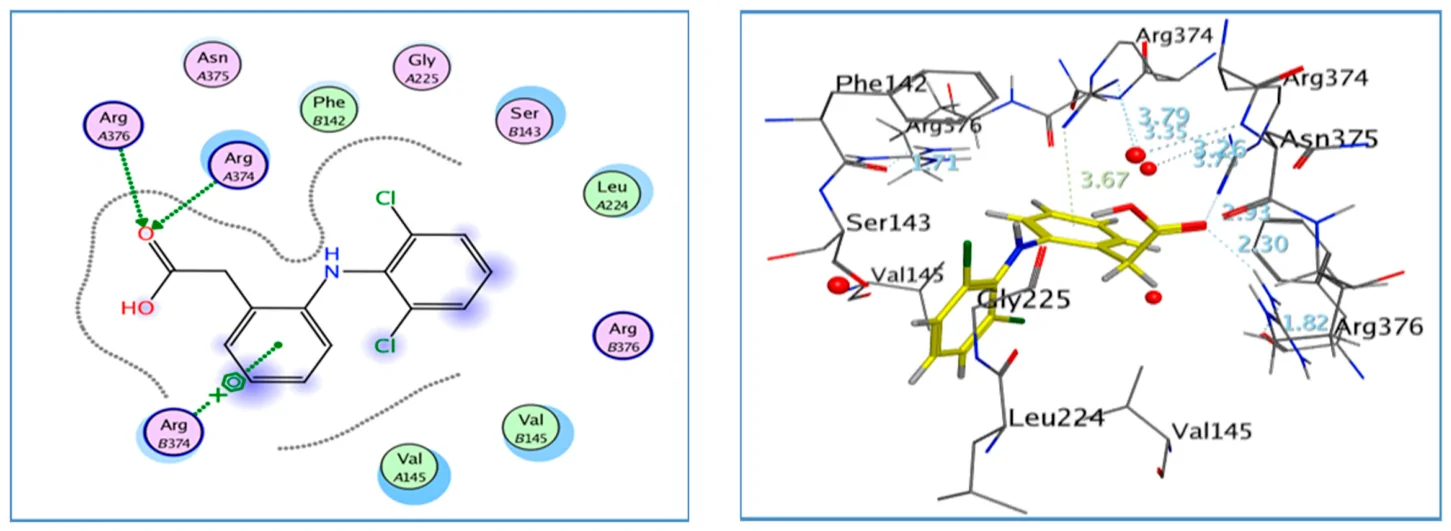
Molecular docking of diclofenac into the COX-1 active site: 2D diagram and 3D diagram representations showing its binding interactions with the amino acid residues.

**Figure 9:**
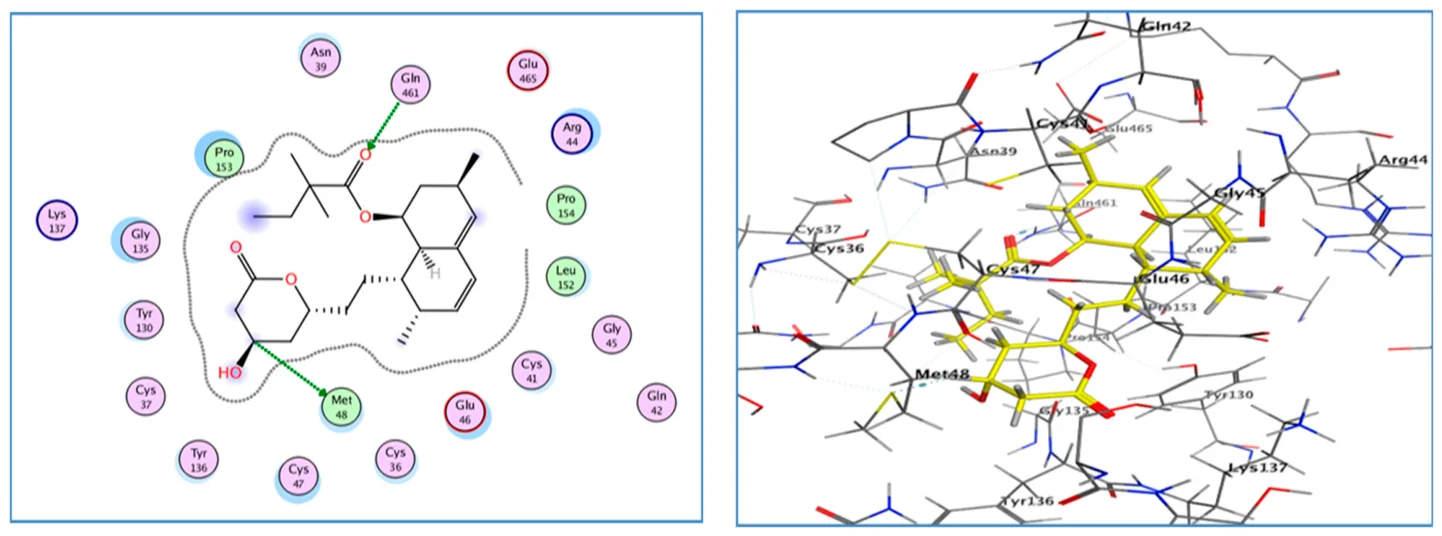
Molecular docking of SMV into the COX-2 active site: 2D diagram and 3D diagram representations showing its binding interactions with the amino acid residues.

**Figure 10:**
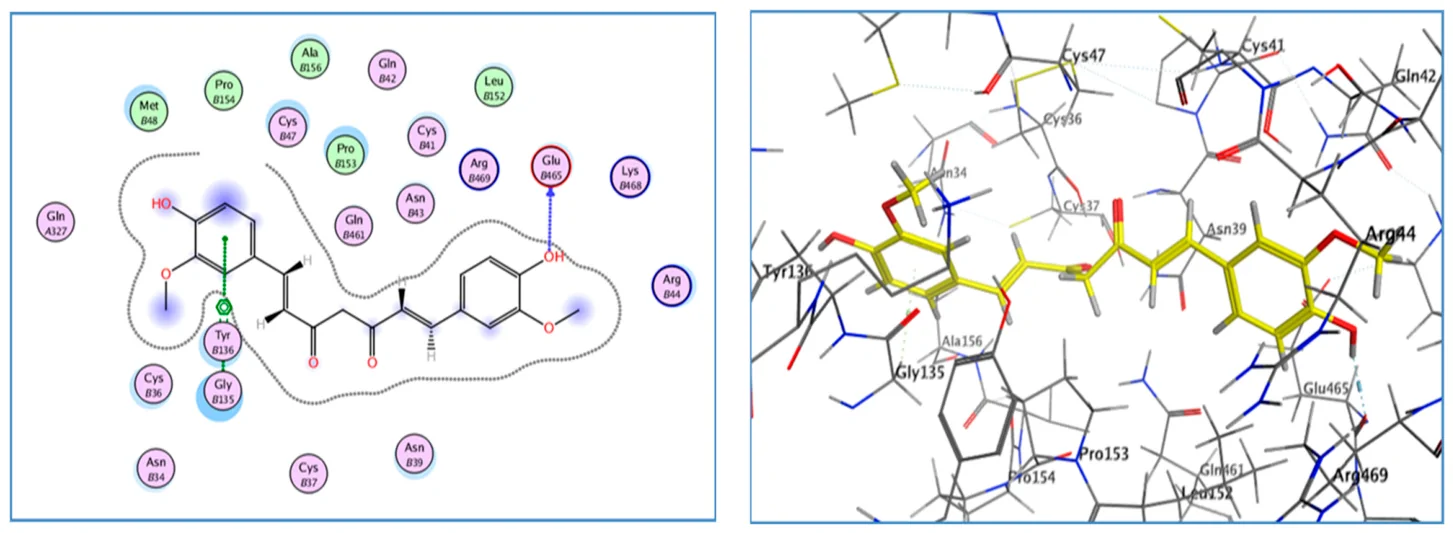
Molecular docking of Curc into the COX-2 active site: 2D diagram and 3D diagram representations showing its binding interactions with the amino acid residues.

**Figure 11:**
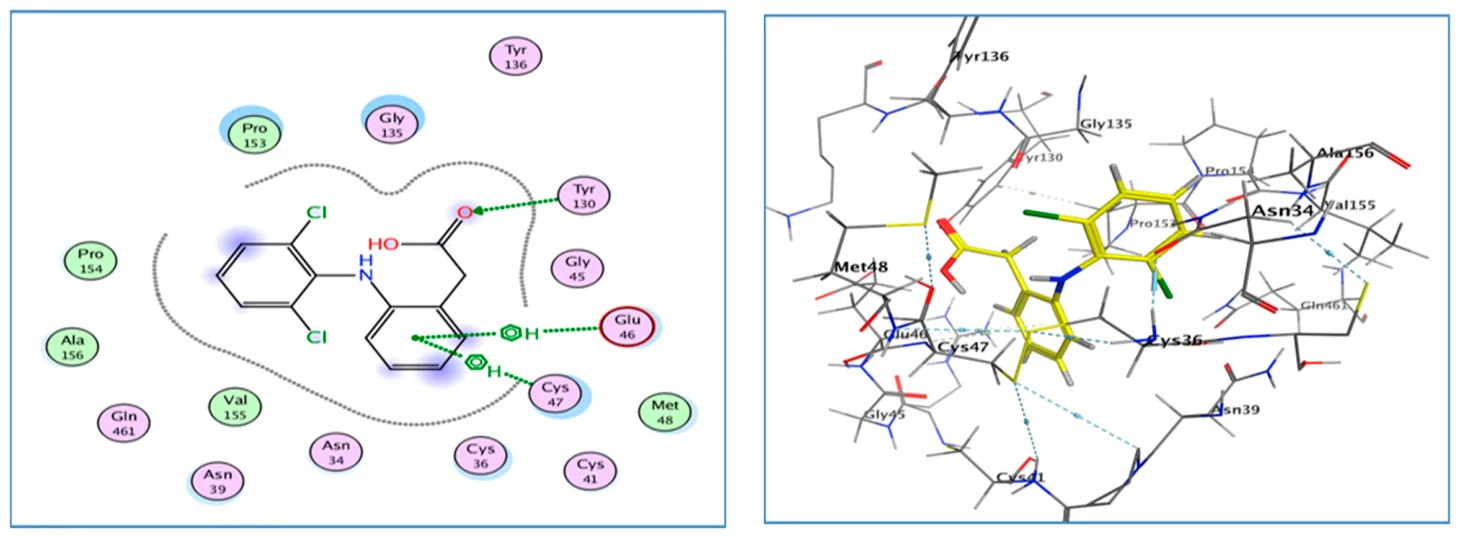
Molecular docking of diclofenac into the COX-2 active site: 2D diagram and 3D diagram representations showing its binding interactions with the amino acid residues.

**Figure 12:**
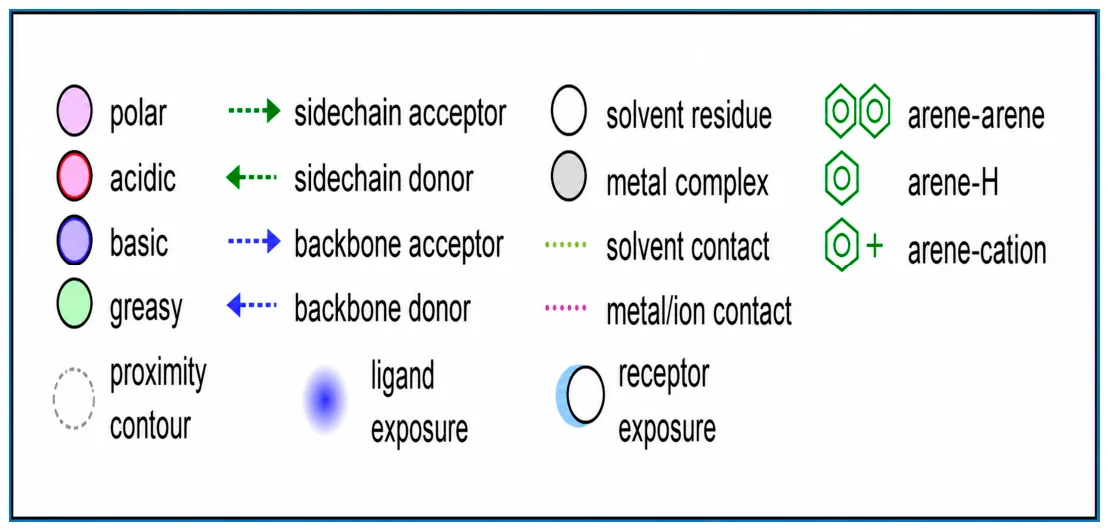
Color scheme for the 2D representations of the interactions between the compounds and the enzyme.

The binding sites of the co-crystalized ligands were selected for the docking experiments, and the coordinates of these binding sites are listed. The 2D and 3D ligand interactions for Curc and SMV and the diclofenac reference drug were saved as picture files. The color indications of these pictures are shown in Figure 6, Figure 7, Figure 8, Figure 9, Figure 10, Figure 11 and Figure 12 and Tables S1 and S2 (see Tables S1 and S2 in the Supplementary Materials).

##### Docking Analysis of Curc, SMV, and Diclofenac Inside COX-1 Active Site

Molecular docking studies were performed against COX-1 enzyme (PDB entry: 3N8Y) to explore the potential binding interactions of the combined compounds. Prior to the analysis, the docking methodology was validated by reproducing the binding pose of the co-crystallized ligand, achieving an RMSD value below 2.0 Å, which established the robustness of the protocol, as shown in Table S1 (see Table S1 in the Supplemental Material) [73,74,75].

SMV and Curc exhibited strong binding affinities with docking scores of −8.048 and −6.971 kcal/mol, respectively, comparable to the native ligand diclofenac (−5.676 kcal/mol). These compounds formed key interactions, including hydrogen bonds (via water bridges) and hydrophobic contacts with essential amino acid residues, such as Arg A374, Arg A376, Arg B374, Glu B140, Asp A236, Ser B143, Arg A333, Asn B144, Val A145, and Phe A142 (Figure 6, Figure 7 and Figure 8). The docking results provide a structural rationale for targeted treatments for wound healing and inflammation.

##### Docking Analysis of Curc and SMV and Diclofenac Inside COX-2 Active Site

Molecular docking studies were performed against COX-2 enzyme (PDB entry: 1CX2), to explore the potential binding interactions of the combined compounds. Prior to the analysis, the docking methodology was validated by reproducing the binding pose of the co-crystallized ligand, achieving an RMSD value below 2.0 Å, which established the robustness of the protocol, as shown in Table S2 (see Table S2 in the Supplementary Materials) [74,76,77].

Curc and SMV exhibited strong binding affinities with docking scores of −6.103 and −7.728 kcal/mol, respectively, comparable to the native ligand diclofenac (−6.239 kcal/mol). These compounds formed key interactions including hydrogen bonds and hydrophobic contacts with essential amino acid residues such as Met B48, Gln B461, Glu B465, Glu B46, Gly B135, Tyr B130, and Cys B47 (Figure 9, Figure 10, Figure 11 and Figure 12). The docking results provide a structural rationale for targeted treatments for wound healing and inflammation.

#### 3.5.3. X-Ray Diffraction

**Figure 13:**
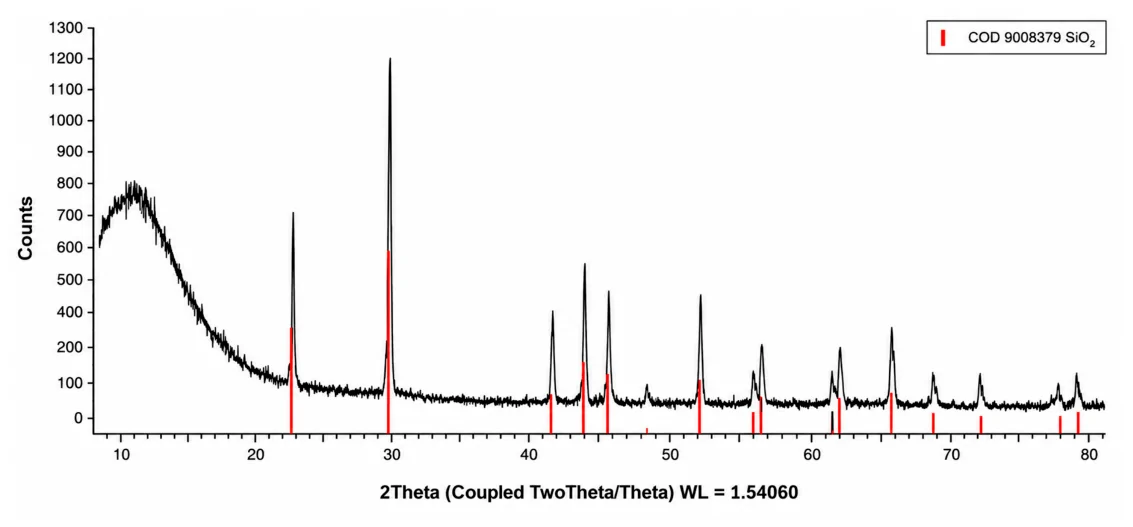
XRD pattern of the synthesized SeNPs.

The crystalline characteristics, phase integrity, and structural alignment of the biosynthesized SeNPs were meticulously assessed using X-ray diffraction (XRD) analysis, as illustrated in Figure 13. The acquired diffractogram displays a sequence of distinct, pronounced, and powerful Bragg reflection peaks situated at 2 θ values of roughly 23.56°, 29.75°, 41.43°, 43.71°, 45.46°, 51.83°, 56.1°, 61.6°, 65.3°, and 78.4°. These distinctive peaks align exactly with the (100), (101), (110), (102), (111), (201), (112), (103), (210), and (212) crystallographic planes, which are in flawless structural concordance with the reference parameters of the standard hexagonal phase of trigonal selenium (t-Se, Crystallography Open Database-COD entry: 9008579). The appearance of these well-defined, narrow Bragg reflections verifies that the produced nanostructures exhibit a clearly organized crystalline lattice configuration. The prominent broad hump or indistinct background seen at lower 2θ angles (10° to 20°) is ascribed to the amorphous characteristics of the phytogenic capping matrix (polyphenols, proteins, and flavonoids derived from the Psidium guajava leaf extract), which sterically surrounds the SeNP cores. The observed diffraction pattern was consistent with crystalline elemental selenium (Se^0^), with the major reflections corresponding to the reported trigonal selenium phase. No additional crystalline peaks attributable to detectable secondary crystalline phases were observed within the detection limits of the XRD analysis [78,79,80,81].

The XRD pattern exhibited characteristic diffraction reflections consistent with the crystalline structure of elemental selenium (Se^0^), supporting the formation of crystalline selenium nanoparticles. No additional prominent diffraction peaks attributable to other crystalline phases were detected within the detection limits of the XRD analysis, suggesting the absence of detectable secondary crystalline phases.

#### 3.5.4. Box–Behnken Designs

A three-level, two-factor Box–Behnken statistical design (BBD) was methodically employed to model, assess, and enhance the essential process variables influencing the co-encapsulation of Curc and SMV within biogenic Curc–SeNP–SMV. The optimization protocol assessed the quantitative impacts of two separate crucial process parameters: the ratio of selenium nanoparticles to total drug (SeNP:D) (C_1_) and the concentration of ethanol (Eth.conc.) (C_2_), as outlined in Table 1. The two independent variables were examined at three separate levels, classified as low (−1), medium (0), and high (+1). The ratios for (SeNP:D) (C_1_) were designated as (0.5:1), (0.75:1), and (1:1), while the operational objectives for ethanol concentration (C_2_) were determined to be 30%, 40%, and 50% *v*/*v*, correspondingly. To evaluate the formulation’s efficacy, three dependent pharmacological responses were meticulously monitored for both integrated therapeutic payloads: encapsulation efficiency (%EE), (E_1_), and the cumulative in vitro percentage drug release after 2 h (E_2_) and 8 h (E_3_) of dissolution.

A detailed compilation of the experimental trials and their associated observed multi-cargo reactions is encapsulated are show in Table 2. The documented response values (E_1_) (%EE) exhibited considerable variability, reaching a zenith of (94.71%) for SMV and (93.92%) for Curc in formulation F9, while the nadir loading values of (63.12%) (SMV) and (67.30%) (Curc) were noted in formulation F8. At the initial dissolution checkpoint (E2) (Rel_2_ h), the values varied from a peak of (33.52%) (SMV) and (37.62%) (Curc) in formulation F9 to a low of (25.42%) (SMV) and (28.33%) (Curc) in formulation F5. This persistent kinetic pattern was uniformly reflected throughout the comprehensive 8 h extended dissolution period (E3) (Rel_8_ h), with formulation F9 achieving the highest cumulative payload delivery rates (61.63% SMV; 67.92% Curc), whereas formulation F5 demonstrated the lowest cumulative release capability (49.92% SMV; 58.42% Curc), thereby validating a remarkably predictable and mathematically replicable responsive framework [82,83].

##### Entrapment Efficiency of Curc–SeNP–SMV

The entrapment efficiency (%EE) of SMV and Curc in the formulated Curc–SeNP–SMV varied significantly based on the formulation parameters examined.

Table 3 illustrates that F9 demonstrated the greatest %EE for both SMV and Curc, recording values of 94.71% and 93.92%, respectively. Conversely, F8 exhibited the minimal %EE, recording 63.12% for SMV and 67.30% for Curc. Consequently, the formulations were prioritized in decreasing order based on their %EE as follows: F9 > F1 > F6 > F3 > F2 > F7 > F4 > F5 > F8.

##### Efficacy of Simvastatin Entrapment

The effects of the investigated independent formulation variables on the entrapment efficiency of SMV (%EE; E_1_) were evaluated using the polynomial equation generated by the statistical model (Equation (5)):

(5) E_1_ = 80.13 + 11.01C_1_ − 6.36C_2_

where **C_1_** represents the SeNP:D ratio and **C_2_** represents the ethanol concentration (Eth Conc.). The positive coefficient of C_1_ (+11.01) indicates a direct relationship between the SeNP:D ratio and SMV entrapment efficiency, suggesting that increasing the amount of SeNPs relative to the drug enhanced SMV incorporation into the nanoparticulate system. In contrast, the negative coefficient of C_2_ (−6.36) indicates an inverse relationship between ethanol concentration and SMV %EE, whereby increasing the ethanol concentration was associated with a reduction in drug entrapment. Notably, the magnitude of the coefficient for C_1_ was greater than that of C_2_, suggesting that the SeNP:D ratio exerted a more pronounced effect on SMV entrapment efficiency within the investigated experimental domain.

The statistical significance and adequacy of the fitted model were further evaluated by ANOVA, with the corresponding results presented in Table S3 (see Table S3 in the Supplemental Material).

**Figure 14:**
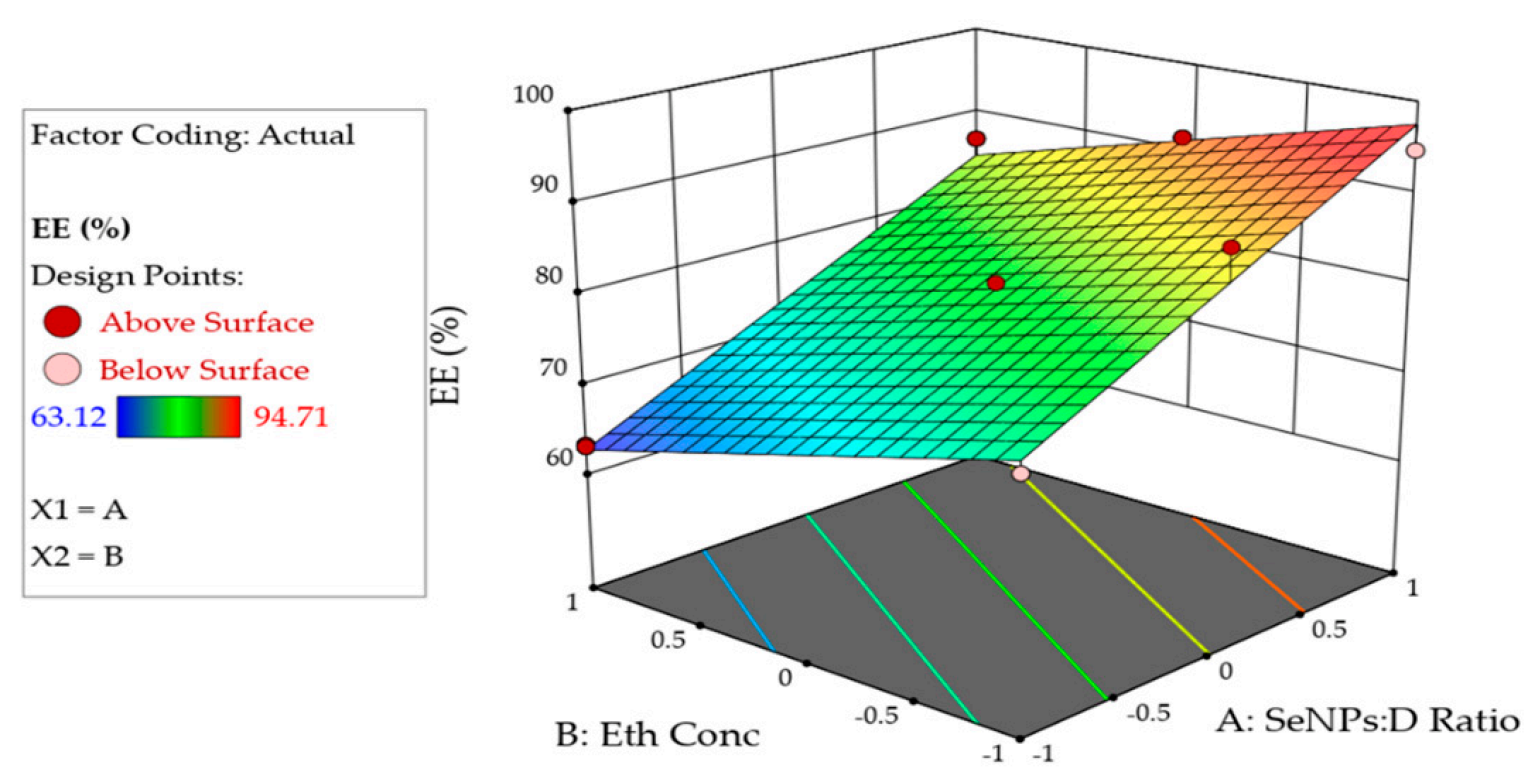
Three-dimensional contour plot for the effect of selenium nanoparticle-to-simvastatin ratio (C_1_) and ethanol concentration (C_2_) on the percentage entrapment efficiency (E_1_).

**Figure 15:**
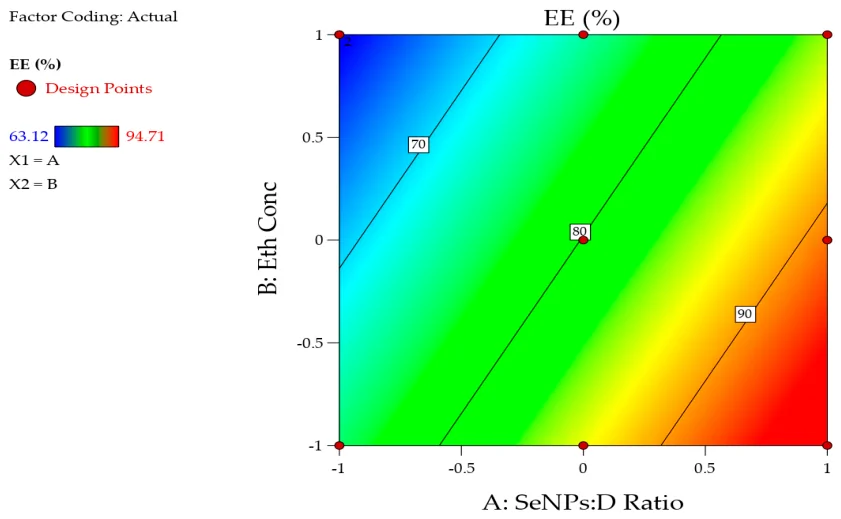
Two-dimensional contour plot for the effect of selenium nanoparticle-to-simvastatin ratio (C_1_) and ethanol concentration (C_2_) on the percentage entrapment efficiency (E_1_).

Furthermore, the effects of C_1_ and C_2_, both individually and in combination, on response E_1_ are graphically illustrated by the three-dimensional response surface plot and two-dimensional contour plots presented in Figure 14 and Figure 15, respectively. These graphical representations provide a visual assessment of the formulation-factor effects and confirm the trends predicted by the polynomial model.

Table 2 illustrates that elevating the ethanol content from 30% to 50% *v*/*v* led to a decrease in SMV %EE across all examined SeNP:D ratios. At the elevated SeNP:D ratio (1:1), E_1_ diminished from 94.71% at 30% *v*/*v* ethanol to 86.84% at 50% *v*/*v*. At the medium ratio (0.75:1), E_1_ diminished from 92.32% to 70.34%, while at the low ratio (0.5:1), it fell from 74.16% to 63.12%. These results validate the inverse relationship between ethanol content and SMV entrapment efficiency. Conversely, augmenting the SeNP:D ratio improved the entrapment effectiveness of SMV at a constant ethanol concentration. For instance, at a 30% *v*/*v* ethanol concentration, augmenting C_1_ from 0.5:1 to 1:1 elevated E1 from 74.16% to 94.71%. Similarly, at a concentration of 50% *v*/*v* ethanol, augmenting C_1_ from 0.5:1 to 1:1 elevated E1 from roughly 63% to 86.84%. Consequently, C_1_ had a favorable association with SMV %EE.

The maximum SMV entrapment efficiency of 94.71% was achieved with F9, which had a high SeNP:D ratio (1:1) and a low ethanol concentration (30% *v*/*v*). These results suggest that optimizing the SeNP:D ratio and reducing the ethanol concentration within the studied experimental parameters enhances SMV entrapment.

##### Efficacy of Curcumin Entrapment

The influence of the investigated formulation variables on the entrapment efficiency of Curc (%EE; E_1_) was further evaluated using the following polynomial equation (Equation (6)):

(6) **E_1_** = 82.56 + 9.88C_1_ − 5.50C_2_

The model coefficients indicate that C_1_ (SeNP:D ratio) exerted a positive effect on Curc entrapment efficiency, as evidenced by its positive coefficient (+9.88), demonstrating a direct relationship between the SeNP:D ratio and E_1_. In contrast, C_2_ (ethanol concentration) exhibited a negative coefficient (−5.50), indicating an inverse relationship with Curc entrapment efficiency. Thus, increasing the SeNP:D ratio was associated with enhanced Curc entrapment, whereas increasing the ethanol concentration resulted in a reduction in %EE. The ANOVA results for the fitted model are presented in Table S4 (refer to Table S4 in the Supplemental Material).

**Figure 16:**
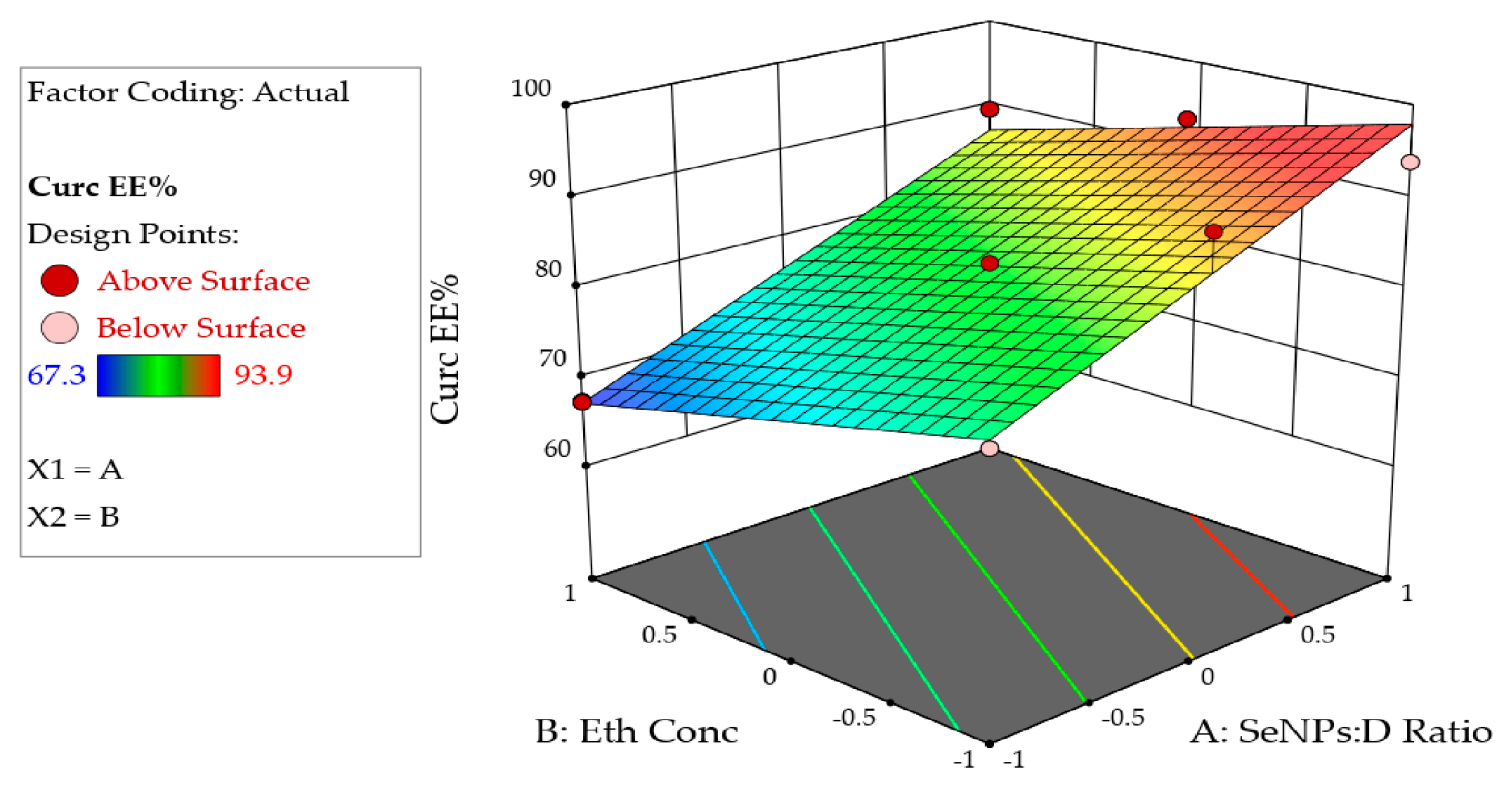
Three-dimensional contour plot for the effect of selenium nanoparticle-to-curcumin ratio (C_1_) and ethanol concentration (C_2_) on the percentage entrapment efficiency (E_1_).

**Figure 17:**
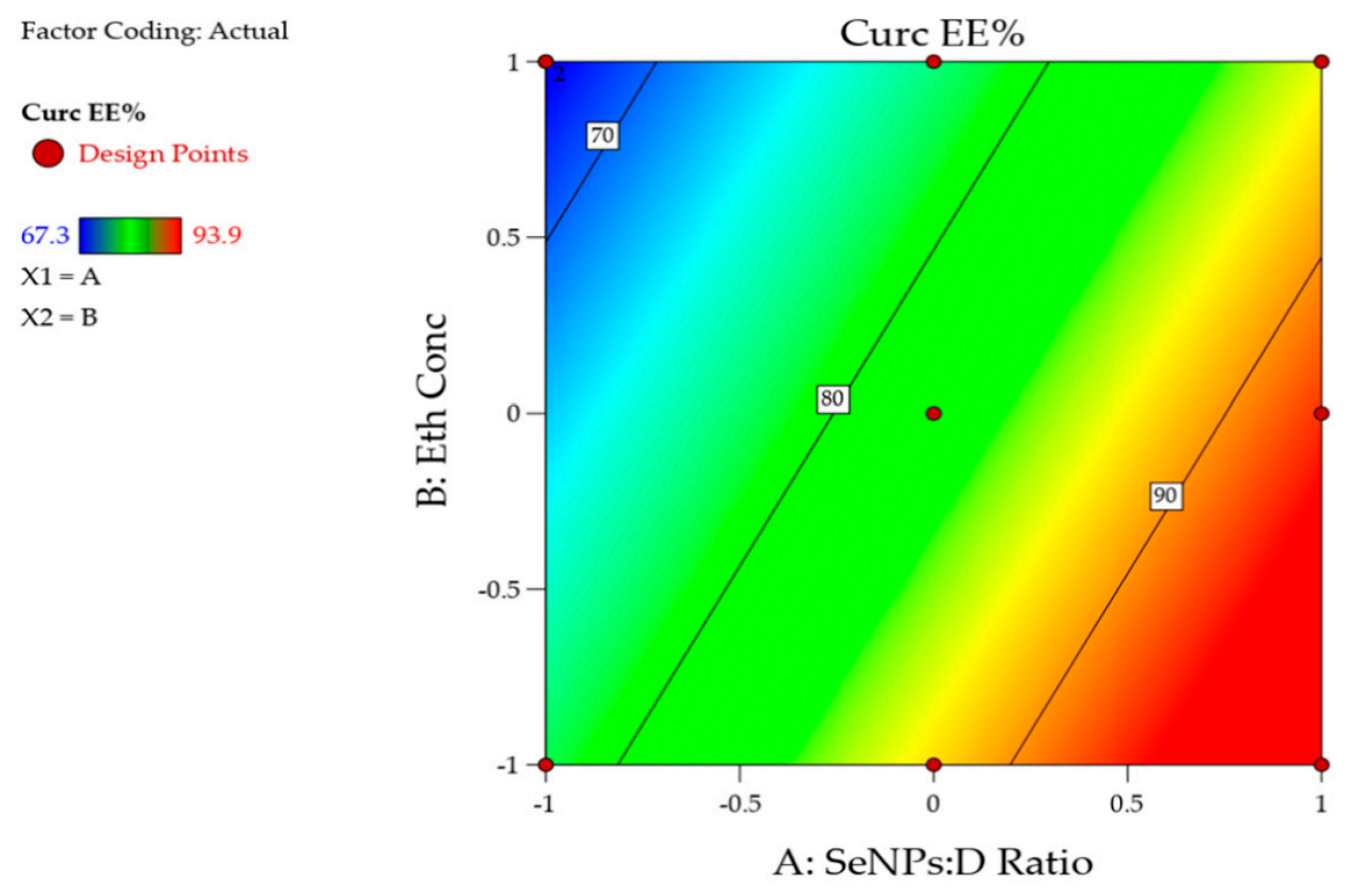
Two-dimensional contour plot for the effect of selenium nanoparticle-to-curcumin ratio (C_1_) and ethanol concentration (C_2_) on the percentage entrapment efficiency (E_1_).

Furthermore, the individual and combined effects of C_1_ and C_2_ on E_1_ are illustrated by the corresponding three-dimensional response surface plot and two-dimensional contour plots shown in Figure 16 and Figure 17, respectively.

Table 2 illustrates that elevating the ethanol concentration from 30% to 50% *v*/*v* led to a significant reduction in Curc %EE across various SeNP:D ratios. At the elevated C_1_ tier (1:1), E_1_ diminished from 93.92% to 89.50%, but at the moderate C_1_ tier (0.75:1), it fell from 92.32% to 73.51%. At the low C_1_ tier (0.5:1), the percentage of Curc encapsulation efficiency diminished from 77.31% to 67.30%. Consequently, the concentration of ethanol demonstrated an inverse relationship with the effectiveness of Curc entrapment.

On the other hand, augmenting the SeNP:D ratio led to a rise in Curc %EE at a constant ethanol concentration. At a 30% *v*/*v* ethanol concentration, augmenting C_1_ from 0.5:1 to 1:1 elevated E1 from 77.31% to 93.92%. In a comparable manner, at 50% *v*/*v* ethanol, augmenting the SeNP:D ratio led to a rise in E_1_ from 67.30% to 89.50%. These results validate the favorable relationship between the SeNP:D ratio and the effectiveness of Curc entrapment.

The highest Curc entrapment efficiency (93.92%) was recorded for F9, which was formulated with a high SeNP:D ratio (1:1) and a low ethanol concentration (30% *v*/*v*). Consequently, the same formulation parameters that selected SMV entrapment also resulted in the highest Curc entrapment, indicating that F9 was the most effective formulation for the entrapment efficiency of both pharmaceuticals.

##### In Vitro Drug-Release Study—2 h

The in vitro release characteristics of SMV and Curc from the Curc–SeNP–SMV formulation were examined at pH 7.4 after 2 h and 8 h to simulate the pH of wounds. The cumulative drug release at 2 and 8 h was selected as two representative response points for the response surface methodology (RSM) analysis. The 2 h response (E_2_) was used to characterize the early release behavior of the formulations, whereas the 8 h response (E_3_) was selected to represent the later release behavior within the experimental release period. Thus, these two time points were used to capture changes in the release profile at different stages and to evaluate the effects of the SeNPs ratio (C_1_) and ethanol concentration (C_2_) on drug release during the studied period.

The release data obtained at these time points were used as formulation responses for the Box–Behnken design, while the complete release profile was considered separately to describe the overall release behavior of the selected formulation. Therefore, the 2 and 8 h responses were not intended to represent the entire duration of drug release, but rather to provide representative early and later release measurements for formulation optimization.

The examined formulations comprising Curc–SeNP–SMV (F1–F9) can be organized in a decreasing sequence based on the in vitro release of (SMV) and (Curc) following a 2 h dissolution time (E_2_), as illustrated in Table 4, as follows:

For SMV and Curc:- F9, F1, F6, F7, F2, F3, F4, F8, F5.

The examined formulations comprising Curc–SeNP–SMV (F1–F9) can be organized in descending order based on the in vitro release of SMV and Curc after a complete dissolving duration of 8 h (E3), as illustrated in Table 5, as follows:

For SMV: F9, F6, F1, F2, F3, F7, F4, F8, F5.

For Curc: F9, F1, F6, F2, F7, F3, F4, F8, F5.

Analysis of Table 6 indicates that F9, with a SeNP:D ratio of (1:1) and an Eth.conc. of 30 (%*v*/*v*), is the superior formulation, achieving the highest rank among the nine tested.

For SMV, the correlation between the independent variables and the cumulative drug release after 2 h (E2) was articulated by the subsequent polynomial equation (Equation (7)):

(7) **E_2_** = 29.73 + 1.36C_1_ − 2.57C_2_

The formula delineates the numerical impact of process variables (C_1_ and C_2_) and their interrelations on the outcome (E_2_). The coefficients C_1_ and C_2_ are associated with the influence of these variables on the answer (E_2_). A positive symbol indicates a collaborative influence, whereas a negative symbol suggests a conflicting effect.

**Figure 18:**
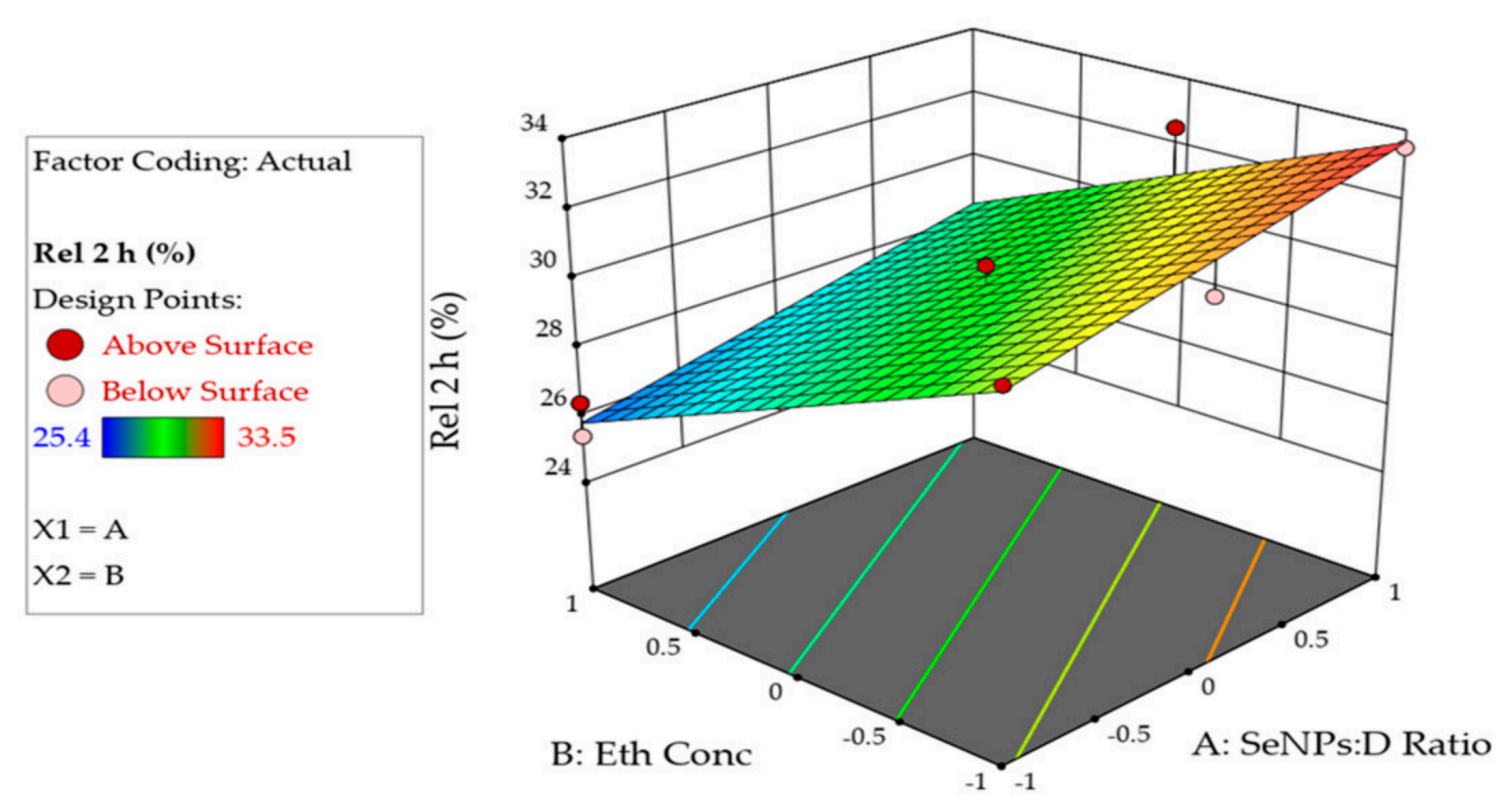
Three-dimensional contour plot for the effect of selenium nanoparticle-to-simvastatin ratio (C_1_) and ethanol concentration (C_2_) on the cumulative percentage release after 2 h (E_2_).

**Figure 19:**
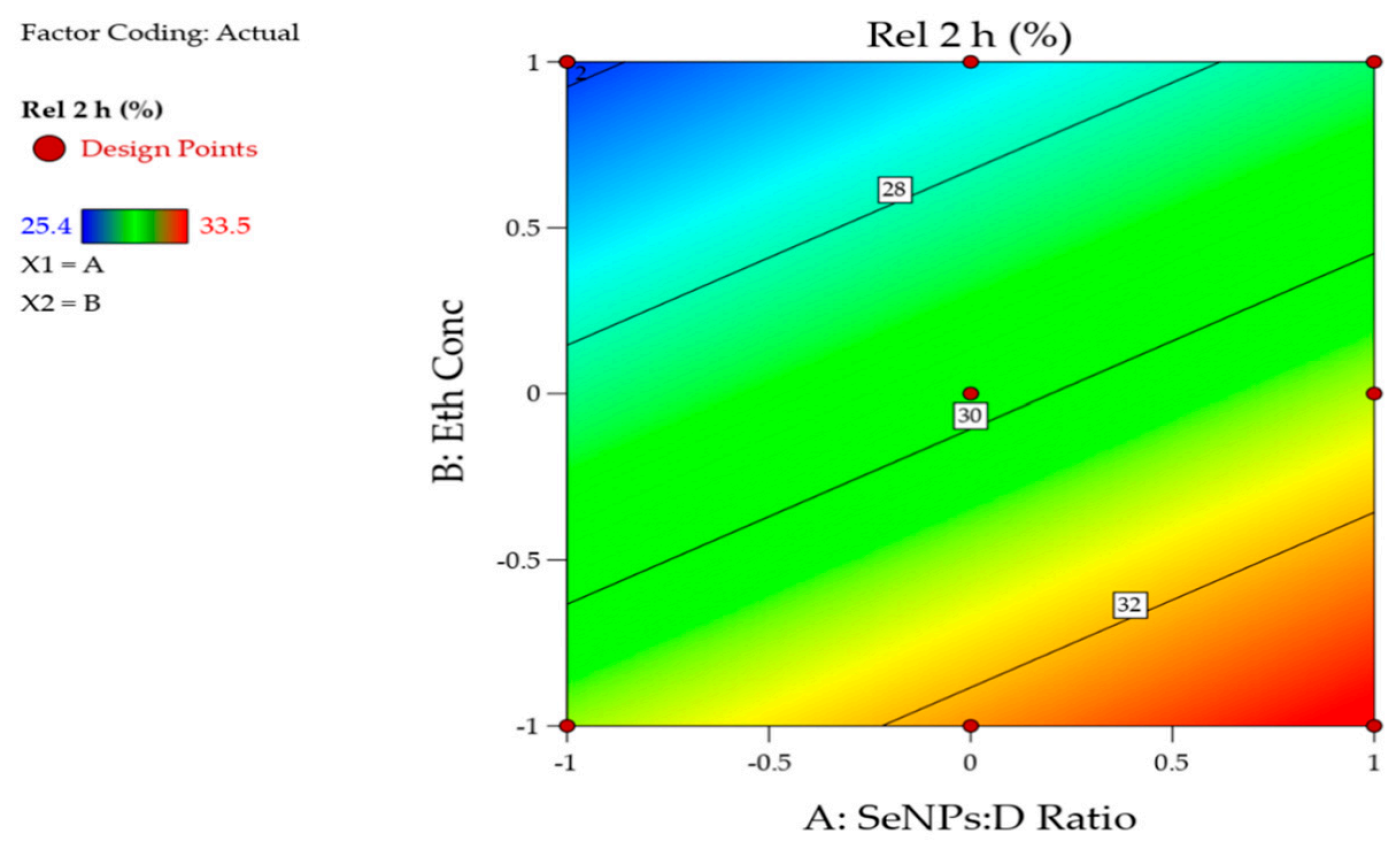
Two-dimensional contour plot for the effect of selenium nanoparticle-to-simvastatin ratio (C_1_) and ethanol concentration (C_2_) on the cumulative percentage release after 2 h (E_2_).

Table S5 (see Table S5 in the Supplemental Material) presents the ANOVA results for the quadratic model of cumulative percentage release following a 2 h period. The association between the dependent and independent variables was additionally illustrated by contour plots. Figure 18 illustrates the influence of factors C_1_ and C_2_ on the response E_2_ using three-dimensional contour plots, whereas Figure 19 depicts the impact of factors C_1_ and C_2_ on the response E2 via two-dimensional contour plots.

In Table 2, it is evident that for SMV, the cumulative drug release after 2 h (%Rel_2_ h; E_2_) was inversely affected by ethanol concentration (C_2_), whereas increasing the SeNP:D ratio (C_1_) generally enhanced the initial drug release. At a constant C_1_, increasing C_2_ from 30% to 50% (*v*/*v*) decreased E_2_ from 31.11% to 26.43% at C_1_ = 0.5:1, from 31.31% to 26.92% at C_1_ = 0.75:1, and from 33.52% to 27.61% at C_1_ = 1:1. Conversely, at a constant C_2_, increasing C_1_ generally increased E_2_, with the highest release (33.52%) observed at C_1_ = 1:1 and C_2_ = 30% (*v*/*v*) compared with 31.11% at C_1_ = 0.5:1 under the same ethanol concentration. Overall, these findings indicate a negative effect of ethanol concentration and a positive effect of the SeNP:D ratio on E_2_, suggesting that lower ethanol concentrations combined with higher SeNP:D ratios favor greater initial drug release. The effects of C_1_ and C_2_ and their potential interaction were further quantified using the fitted polynomial model.

For Curc, the correlation between the independent variables and the cumulative drug release after 2 h (E2) was articulated by the subsequent polynomial equation (Equation (8)):

(8) **E_2_** = 34.97 + 1.25C_1_ − 3.30C_2_ − 0.1435C_1_C_2_ + 0.6185C_1_^2^ − 2.65C_2_^2^

**Figure 20:**
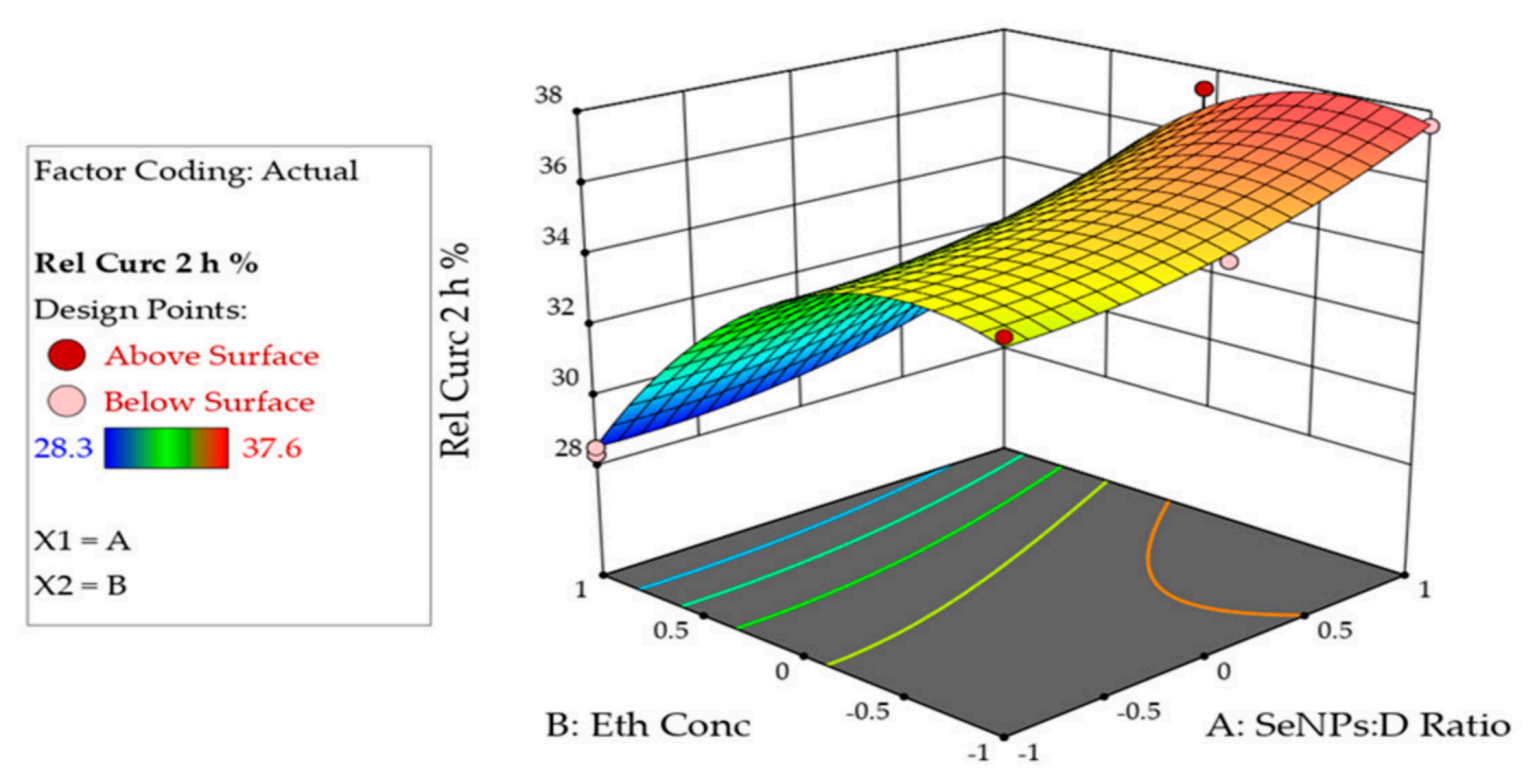
Three-dimensional contour plot for the effect of selenium nanoparticle-to-Curc ratio (C_1_) and ethanol concentration (C_2_) on the cumulative percentage release after 2 h (E_2_).

**Figure 21:**
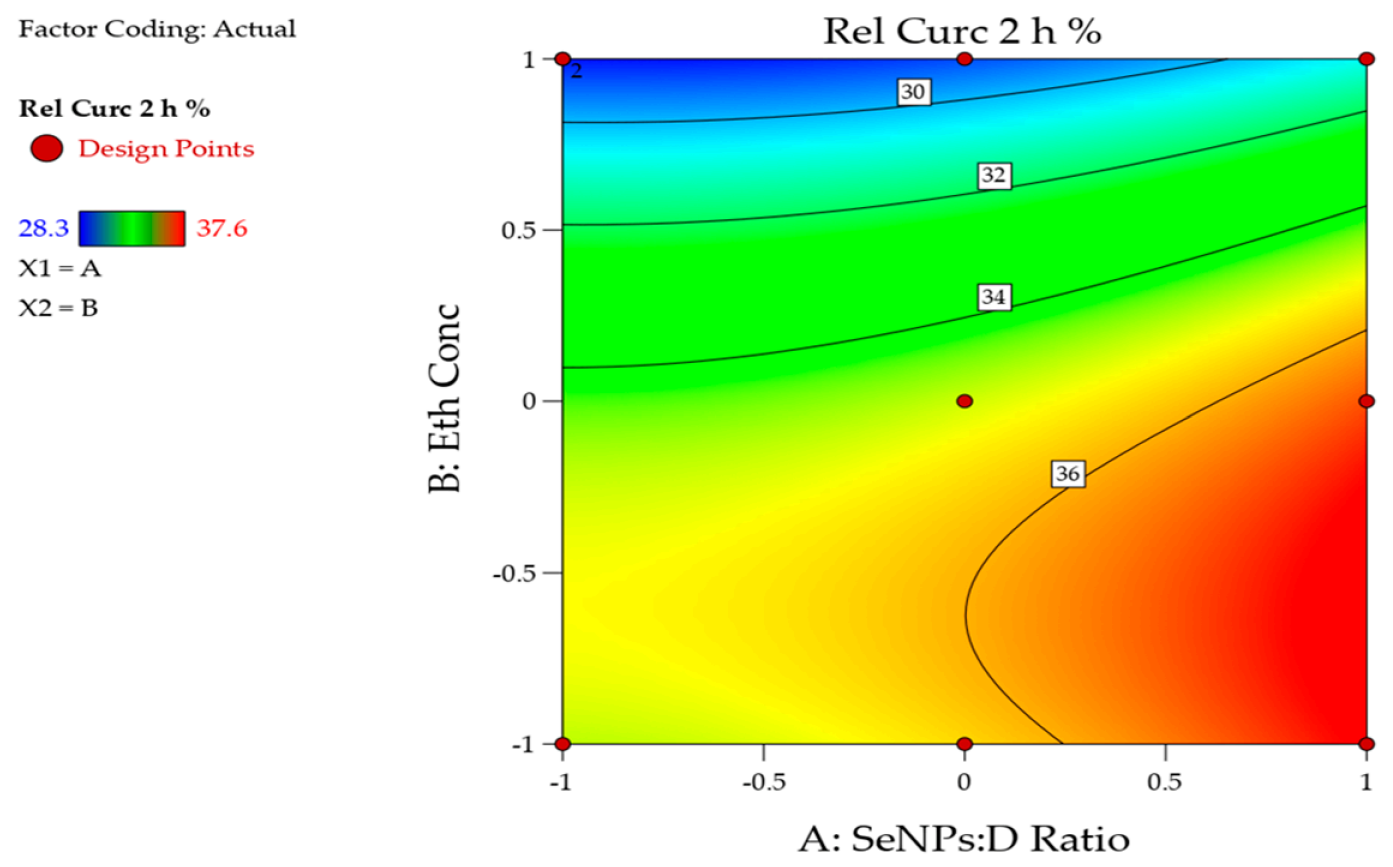
Two-dimensional contour plot for the effect of selenium nanoparticle-to-Curc ratio (C_1_) and ethanol concentration (C_2_) on the cumulative percentage release after 2 h (E_2_).

The formula delineates the numerical impact of process variables (C_1_ and C_2_) and their interrelations on the outcome (E_2_). The coefficients C_1_ and C_2_ are associated with the influence of these variables on the answer (E_2_). Coefficients that include multiple factor terms and those with elevated order terms signify interaction terms and quadratic associations, as illustrated in Figure 20 and Figure 21. A positive symbol indicates a collaborative influence, whereas a negative symbol suggests a conflicting effect. Table S6 (see Table S6 in the Supplemental Material) presents the ANOVA results for the quadratic model of cumulative percentage release following a 2 h period.

##### In Vitro Drug-Release Study 8 h

The total in vitro release of SMV and Curc from the formulated Curc–SeNP–SMV was assessed after 8 h at pH 7.4. The impact of the examined formulation parameters, specifically the SeNP:D ratio (C_1_) and ethanol concentration (C_2_), on the total drug release at 8 h (E_3_) was evaluated by a statistical model.

For SMV, the correlation between the independent variables and the cumulative drug release after 8 h (E_3_) was articulated by Equation (9):

(9) **E_3_** = 56.32 + 2.88C_1_ − 2.88C_2_ − 0.0978C_1_C_2_ − 0.9147C_1_^2^ + 0.2750C_2_^2^

C_1_ denotes the SeNP:D ratio, whereas C_2_ signifies the concentration of ethanol. The affirmative linear coefficient of C_1_ signifies a beneficial impact of the SeNP:D ratio on SMV release, whereas the negative coefficient of C_2_ denotes a contrary influence of ethanol concentration. The C_1_C_2_ interaction term and the quadratic terms signify the existence of interaction and nonlinear influences of the examined factors on the release response.

**Figure 22:**
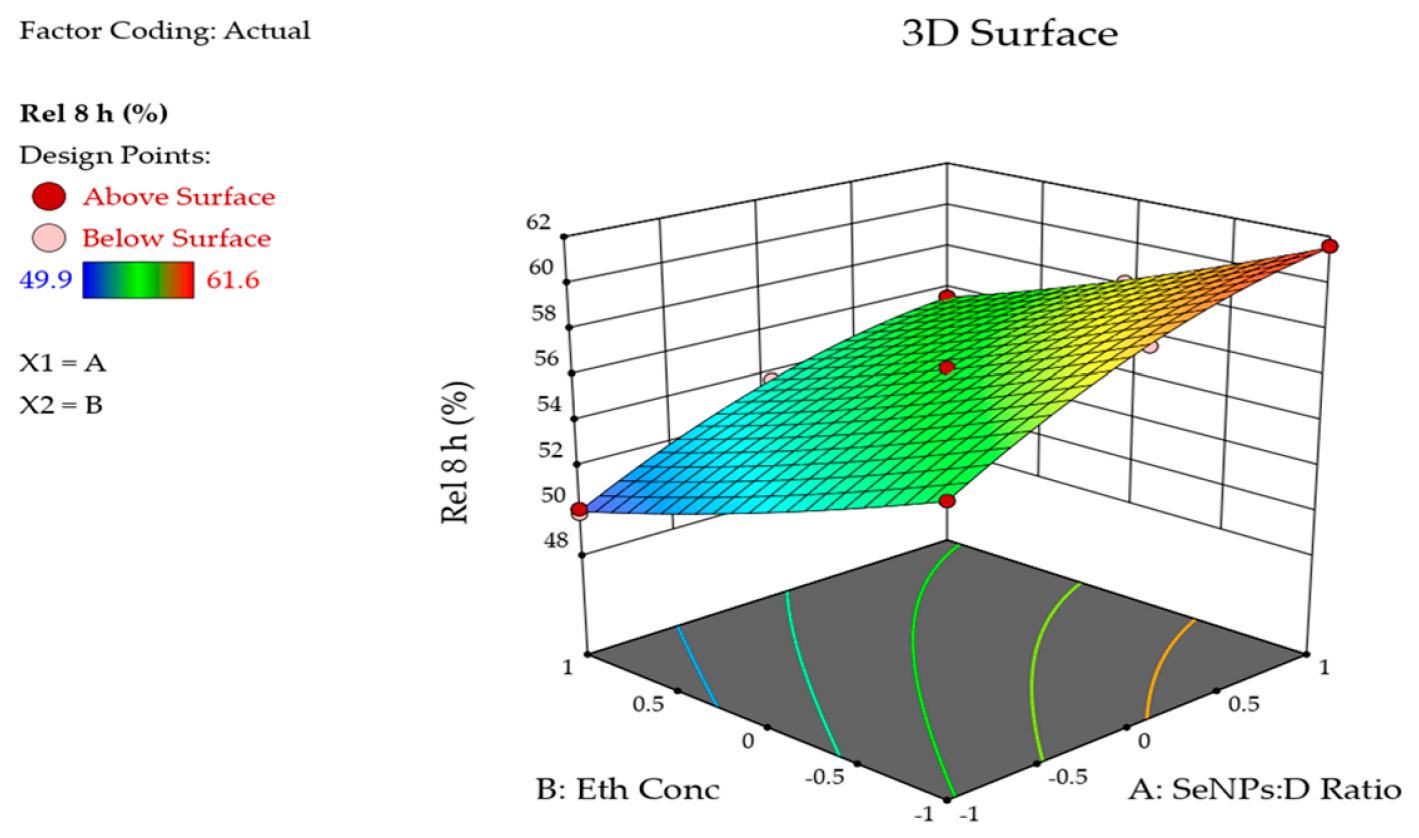
Three-dimensional contour plot for the effect of selenium nanoparticle-to-simvastatin ratio (C_1_) and ethanol concentration (C_2_) on the cumulative percentage release after 8 h (E_3_).

**Figure 23:**
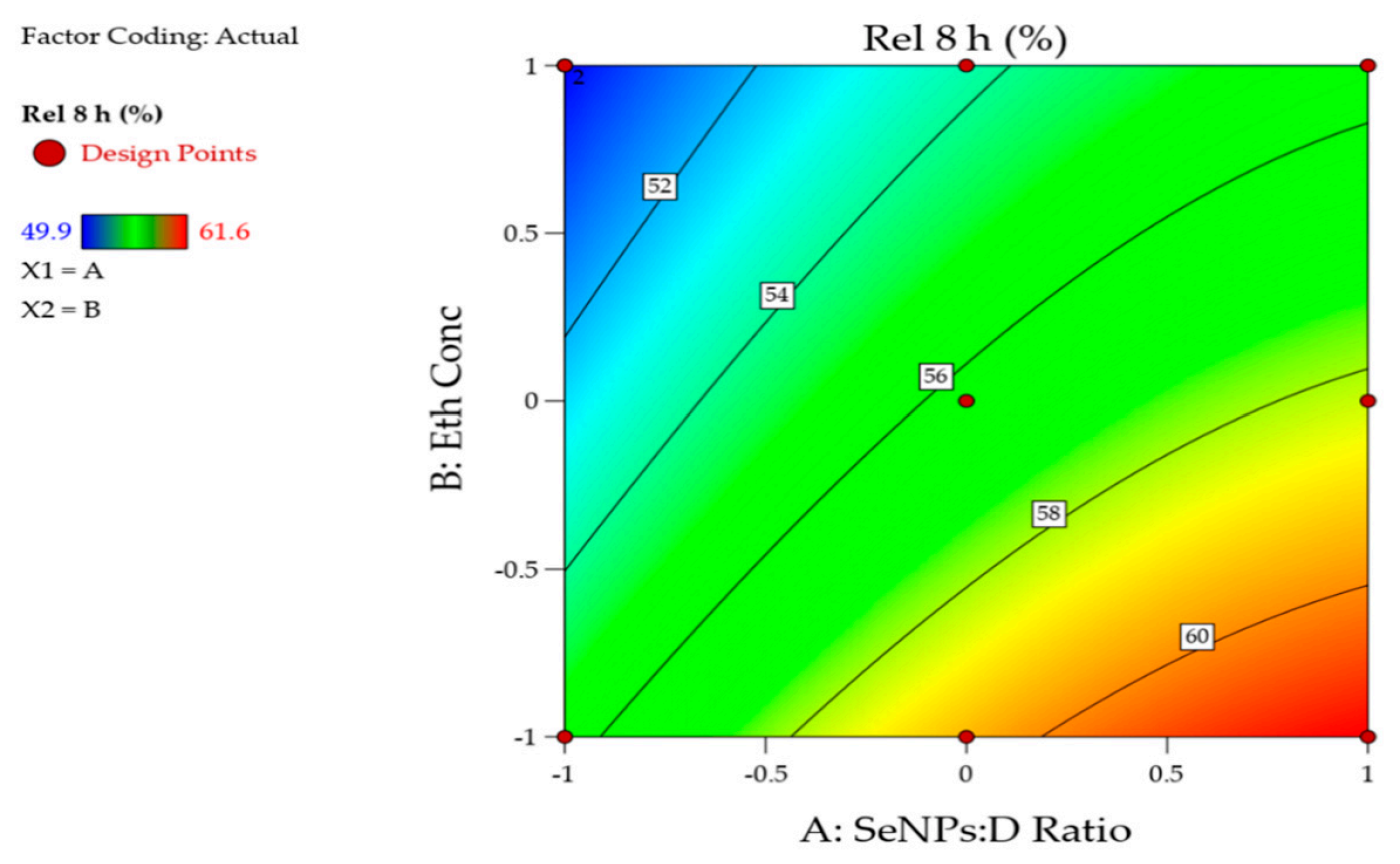
Two-dimensional contour plot for the effect of selenium nanoparticle-to-simvastatin ratio (C_1_) and ethanol concentration (C_2_) on the cumulative percentage release after 8 h (E_3_).

The outcomes of the ANOVA for the quadratic model are displayed in Table S7 (refer to Table S7 in the Supplemental Material). The impact of C_1_ and C_2_ on the total SMV release after 8 h is further depicted in the three-dimensional and two-dimensional contour plots shown in Figure 22 and Figure 23, respectively.

Table 2 illustrates that elevating the ethanol concentration led to a reduction in the total SMV release at constant SeNP:D ratios. At the elevated SeNP:D ratio (1:1), E_3_ diminished from 58.24% to 55.67% as the ethanol concentration rose from 40% to 50% *v*/*v*. In a comparable manner, E_3_ diminished from 56.41% to 53.70% at the intermediate ratio (0.75:1). At the minimal ratio (0.5:1), the total release diminished from around 55.62% to 50.13%. Consequently, elevated ethanol concentration had a detrimental impact on the 8 h release of SMV.

Conversely, augmenting the SeNP:D ratio typically improved the total SMV release at a constant ethanol concentration. At a 30% *v*/*v* ethanol concentration, E_3_ rose from 55.62% at the lower SeNP:D ratio (0.5:1) to 61.63% at the elevated level (1:1). In a comparable manner, at a 50% *v*/*v* ethanol concentration, augmenting the SeNP:D ratio elevated the total release from roughly 50% to 55.67%. These results indicate a generally favorable correlation between the SeNP:D ratio and total SMV emission.

The greatest total SMV release after 8 h was achieved with F9 (61.63%), which had the highest SeNP:D ratio (1:1) and the lowest ethanol content (30% *v*/*v*). This formulation consequently yielded the optimal amalgamation of the examined factors for enhancing SMV release during the 8 h dissolving interval.

For Curc, the correlation between the independent variables and the cumulative drug release after 8 h (E_3_) was articulated by the Equation (10):

(10) E_3_ = 65.42 + 1.38C_1_ − 3.27C_2_ − 0.1457C_1_C_2_ + 0.2832C^2^_1_ − 2.68C^2^_2_

C_1_ and C_2_ denote the ratio of SeNPs to D and the concentration of ethanol, respectively. The affirmative linear coefficient of C_1_ signifies a beneficial impact of the SeNP:D ratio on Curc release, whereas the negative coefficient of C_2_ denotes a contrary influence of ethanol concentration. The interaction term (C_1_C_2_) and the quadratic terms (C_1_^2^ and C_2_^2^) suggest that the release response was affected by both interaction and nonlinear dynamics among the formulation factors.

**Figure 24:**
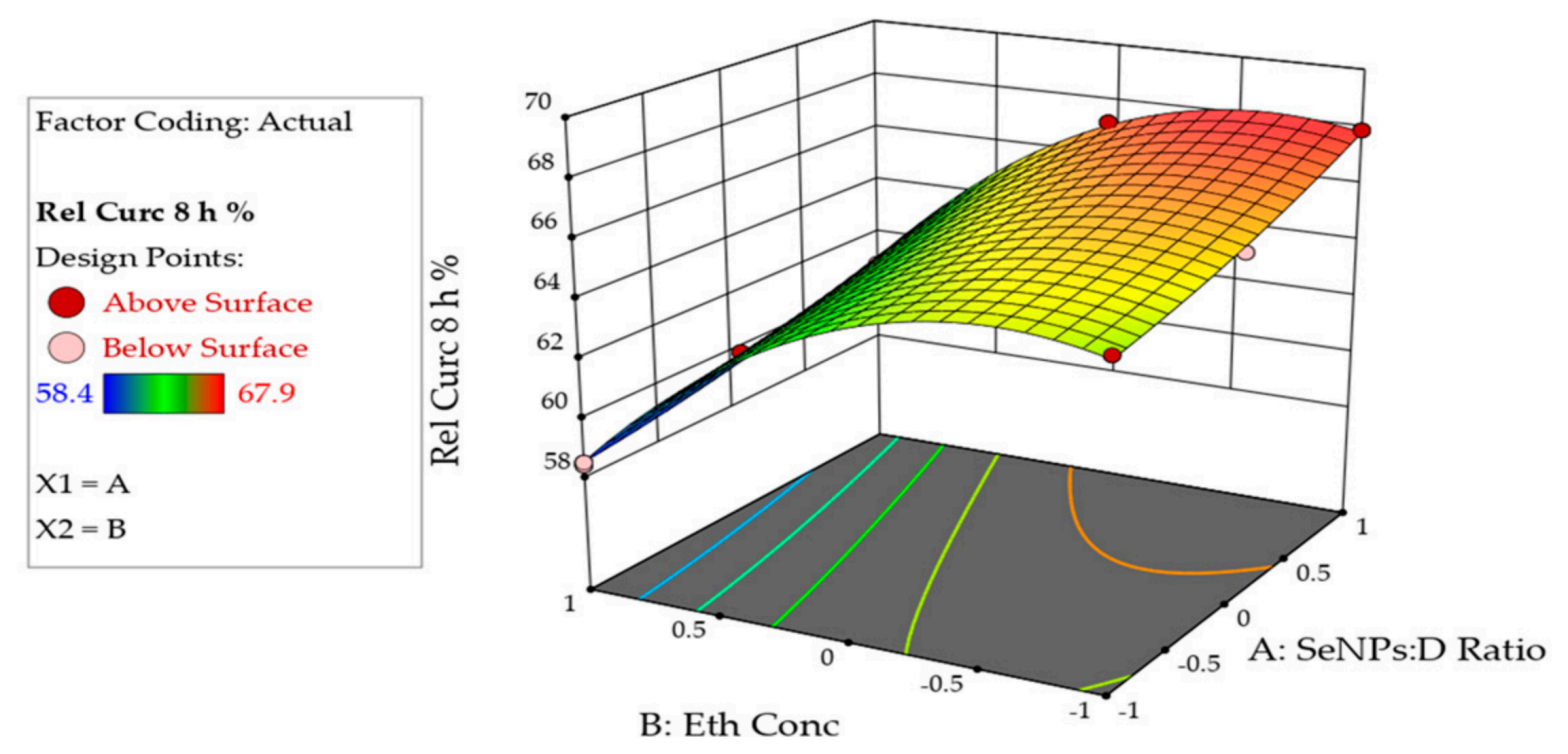
Three-dimensional contour plot for the effect of selenium nanoparticle-to-Curc ratio (C_1_) and ethanol concentration (C_2_) on the cumulative percentage release after 8 h (E_3_).

**Figure 25:**
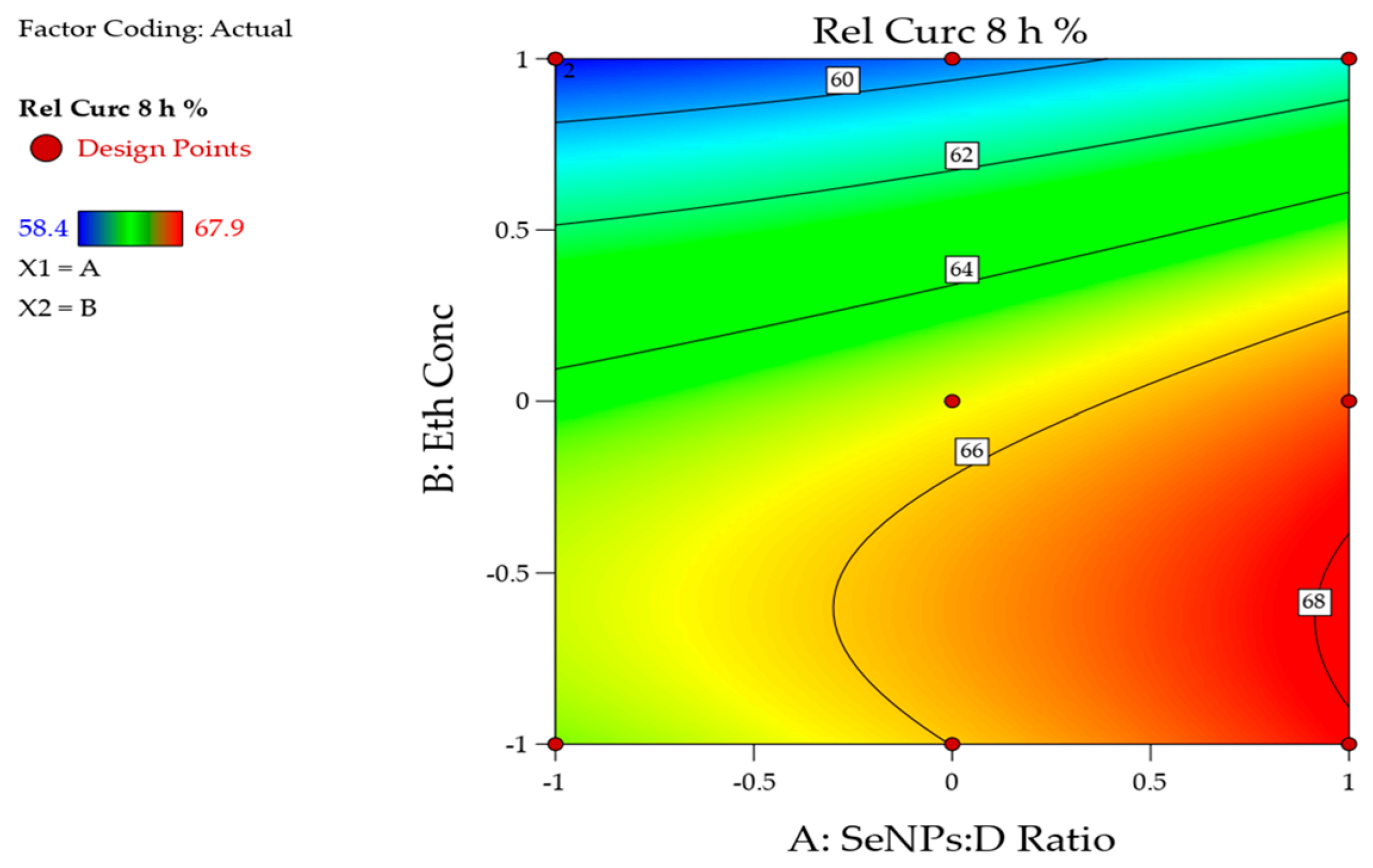
Two-dimensional contour plot for the effect of selenium nanoparticle-to-Curc ratio (C_1_) and ethanol concentration (C_2_) on the cumulative percentage release after 8 h (E_3_).

The outcomes of the ANOVA for the quadratic model are displayed in Table S8 (refer to Table S8 in the Supplemental Material). The relevant three-dimensional and two-dimensional contour graphs depicting the influence of C_1_ and C_2_ on E_3_ are displayed in Figure 24 and Figure 25, respectively.

Table 2 illustrates that elevating the ethanol concentration led to a decrease in the total Curc release after 8 h. At the elevated SeNP:D ratio (1:1), E_3_ diminished from 67.21% to 60.81% when the ethanol concentration escalated from 40% to 50% *v*/*v*. At the intermediate ratio (0.75:1), E3 diminished from 65.32% to 59.80%, and at the lower ratio (0.5:1), the total release fell from roughly 64.91% to 58.53%. These results suggest a general inverse correlation between ethanol concentration and total Curc release.

On the other hand, augmenting the SeNP:D ratio improved the total Curc release at constant ethanol concentrations. At a 30% *v*/*v* ethanol concentration, E3 rose from 64.91% at the lower SeNP:D ratio (0.5:1) to 67.92% at the elevated level (1:1). At a 50% *v*/*v* ethanol concentration, augmenting the SeNP:D ratio correspondingly elevated the total release from roughly 58.5% to 60.81%. Consequently, the SeNP:D ratio demonstrated a generally beneficial impact on the total Curc release.

Peak cumulative Curc release after 8 h was noted with F9 (67.92%), which was formulated utilizing the highest SeNP:D ratio (1:1) and the minimal ethanol concentration (30% *v*/*v*).

Significantly, F9 had the greatest 2 h release for both SMV and Curc, along with the highest entrapment efficiency, suggesting that this formulation composition yielded the most advantageous overall release performance among the examined formulations. The 8 h release results indicated that the SeNP:D ratio and ethanol concentration had contrasting influences on the release of both SMV and Curc. Elevating the SeNP:D ratio enhanced drug release, but a rise in ethanol concentration typically diminished cumulative drug release. The exceptional release efficacy of F9 at both 2 and 8 h further substantiates its designation as the preferred formulation for further investigations.

##### Selection of the Best Optimized and Suggested Formula

**Figure 26:**
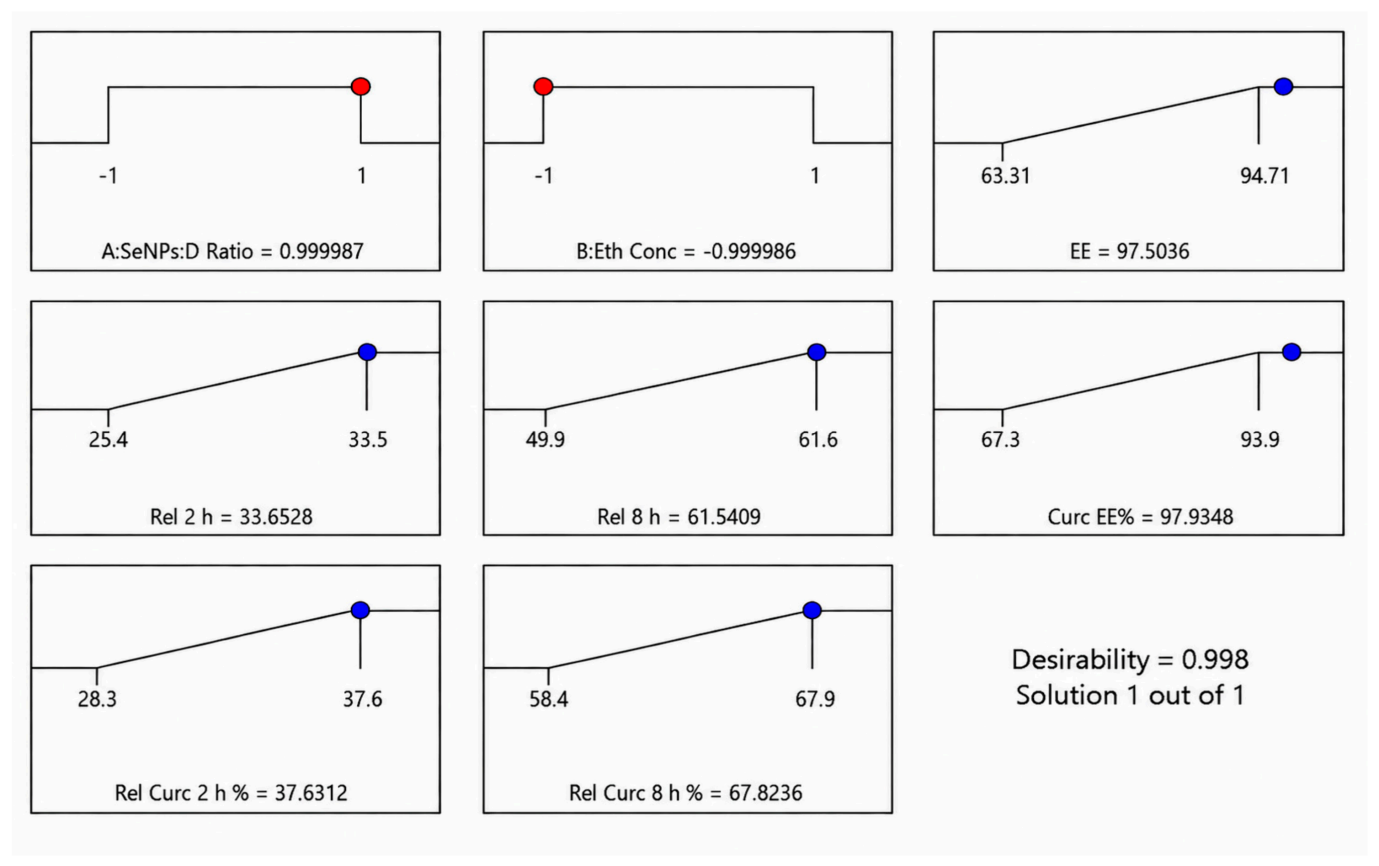
Suggested optimized formulae produced by software.

The optimized formulation was selected using numerical optimization based on the three dependent responses for both drugs Curc and SMV. The optimization results, including the predicted responses and desirability function, are presented in Figure 26 and Tables S9 and S10 (see Tables S9 and S10 in the Supplemental Material). Among the formulations generated by the optimization procedure, formulation F9 was identified as the optimized formulation, achieving a desirability value of 0.998. The experimental responses obtained for F9 were subsequently compared with the corresponding model-predicted responses to assess the agreement between the experimental and predicted values.

#### 3.5.5. FT-IR Spectroscopy (FT-IR)

**Figure 27:**
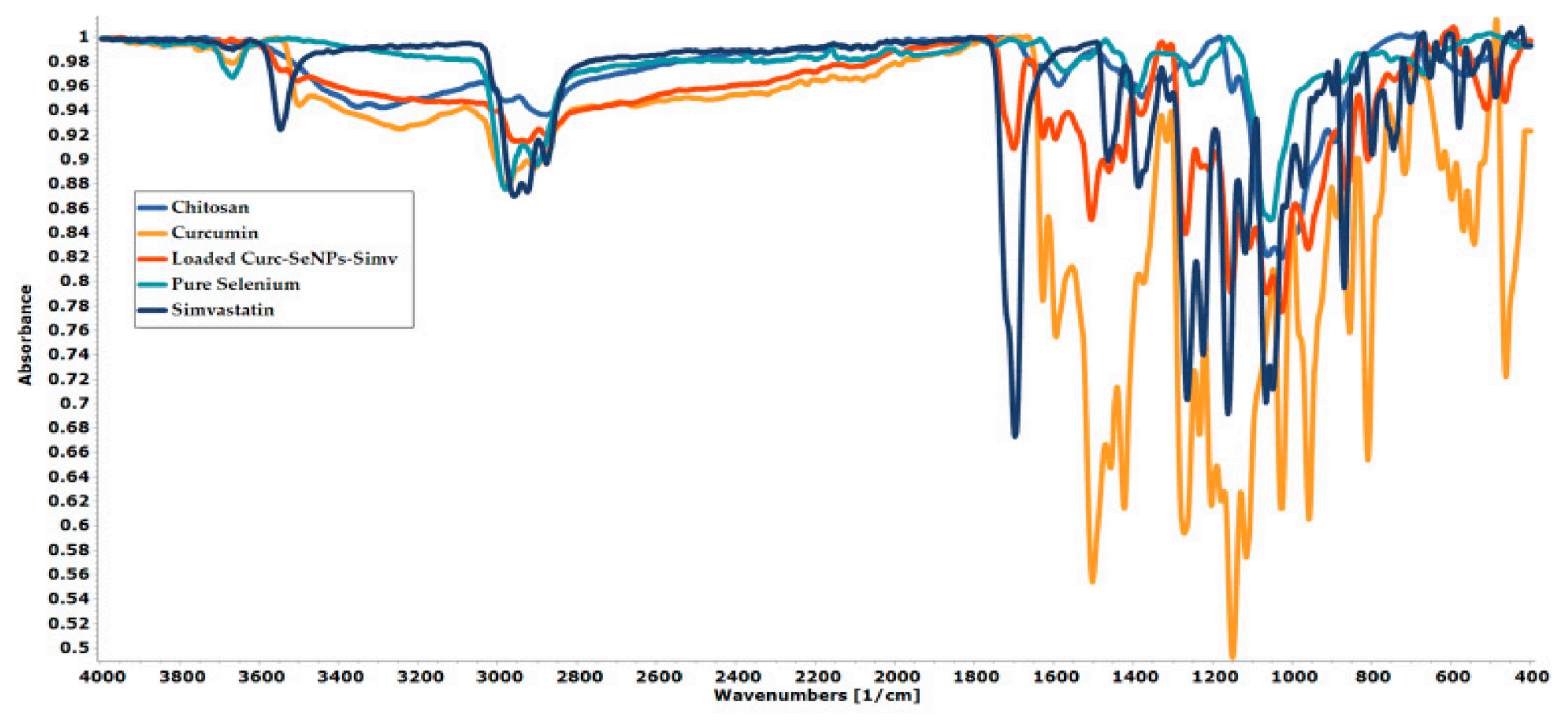
FTIR of pure SeNPs, Curc, SMV, CTS, and Curc–SeNP–SMV.

Fourier-transform infrared spectroscopy was utilized to delineate the primary surface functional groups, discern the distinctive vibrational bands of the analyzed components, and evaluate the chemical compatibility and molecular interactions related to the integration of Curc and SMV into the biogenic, chitosan-capped selenium nanoparticle system Curc–SeNP–SMV (Figure 27).

The FTIR spectrum of unadulterated chitosan (CTS; blue curve) displayed the distinctive absorption peaks of its polysaccharide framework. The extensive band detected in the 3200–3500 cm^−1^ range was mostly ascribed to the superimposed O–H and N–H stretching vibrations, indicating the plentiful hydroxyl and amino groups present in chitosan [84]. The bands seen around 2920–2870 cm^−1^ were linked to aliphatic C–H stretching vibrations. Additionally, the peaks at 1650 and 1590 cm^−1^ can be attributed to amide I (C=O stretching) and amide II/N–H bending vibrations, respectively, hence validating the distinctive chemical composition of CTS [85].

The absorption characteristics of the biogenic SeNPs (turquoise curve) indicated the presence of functional groups derived from phytochemicals found in the Psidium guajava leaf extract. These characteristics align with the involvement of phytogenic chemicals, especially polyphenols and flavonoids, in the reduction, stabilization, and surface capping of SeNPs. The reduction and/or alteration of these bands in relation to the associated plant-derived functional groups may indicate their interaction with the nanoparticle surface during SeNP synthesis [86].

The FTIR spectrum of pure Curc (orange curve) exhibited its distinctive phenolic and conjugated molecular characteristics. The extensive absorption band in the 3200–3500 cm^−1^ range was linked to phenolic O–H stretching, while the bands around 1600–1500 cm^−1^ were primarily ascribed to aromatic C=C and conjugated carbonyl vibrational modes. Supplementary notable bands in the fingerprint region were linked to C–O and C–O–C stretching vibrations, aligning with the ether and phenolic characteristics of Curc [87].

In a similar manner, pure SMV (dark-navy curve) displayed its distinctive functional-group vibrations, featuring a significant ester/lactone C=O stretching band in the vicinity of 1700–1730 cm^−1^. The spectral features detected in the lower-wavenumber range were linked to C–H deformation and C-O/C-C vibrational modes stemming from the aliphatic and lactone-rich composition of SMV [88].

Significantly, the FTIR spectrum of the Curc–SeNP–SMV nanocomposite (red curve) preserved numerous distinctive vibrational characteristics linked to both Curc and SMV, albeit with moderate variations in their intensities and/or precise locations compared to the pure medicines. Such diminutions and slight alterations may be ascribed to non-covalent interactions, encompassing hydrogen bonding and various intermolecular forces among the drug molecules, chitosan functional groups, phytochemical capping agents, and the SeNP surface. These spectrum alterations facilitate the effective integration of Curc and SMV into the nanostructured carrier, indicating that their primary chemical functions have been preserved [89].

In summary, the lack of novel, clearly delineated absorption bands signifying covalent link creation or chemical deterioration reinforces the chemical compatibility of Curc and SMV with the SeNP/CTS matrix. Nevertheless, FTIR results should solely be regarded as indicators of chemical compatibility and molecular interactions, not as conclusive evidence of effective encapsulation. The FTIR results, in conjunction with the drug-loading/entrapment outcomes and further physicochemical characterization data, substantiate the effective creation of the Curc–SeNP–SMV co-loaded nanocarrier, exhibiting no significant chemical alteration of the embedded medicines. FTIR analysis supported the absence of major detectable chemical interactions or substantial chemical alterations of the incorporated components [90].

FTIR analysis was conducted to characterize the major functional groups and assess changes in the corresponding infrared spectral profiles associated with the incorporation of curcumin and simvastatin into the formulation. The observed bands were interpreted as evidence of the functional groups contributed by the plant-derived constituents and the incorporated drugs. These changes support the presence of molecular interactions among the formulation components; however, FTIR data alone do not establish the exact localization of the drugs within the nanoparticles or independently prove encapsulation.

#### 3.5.6. Scanning Electron Microscopy

**Figure 28:**
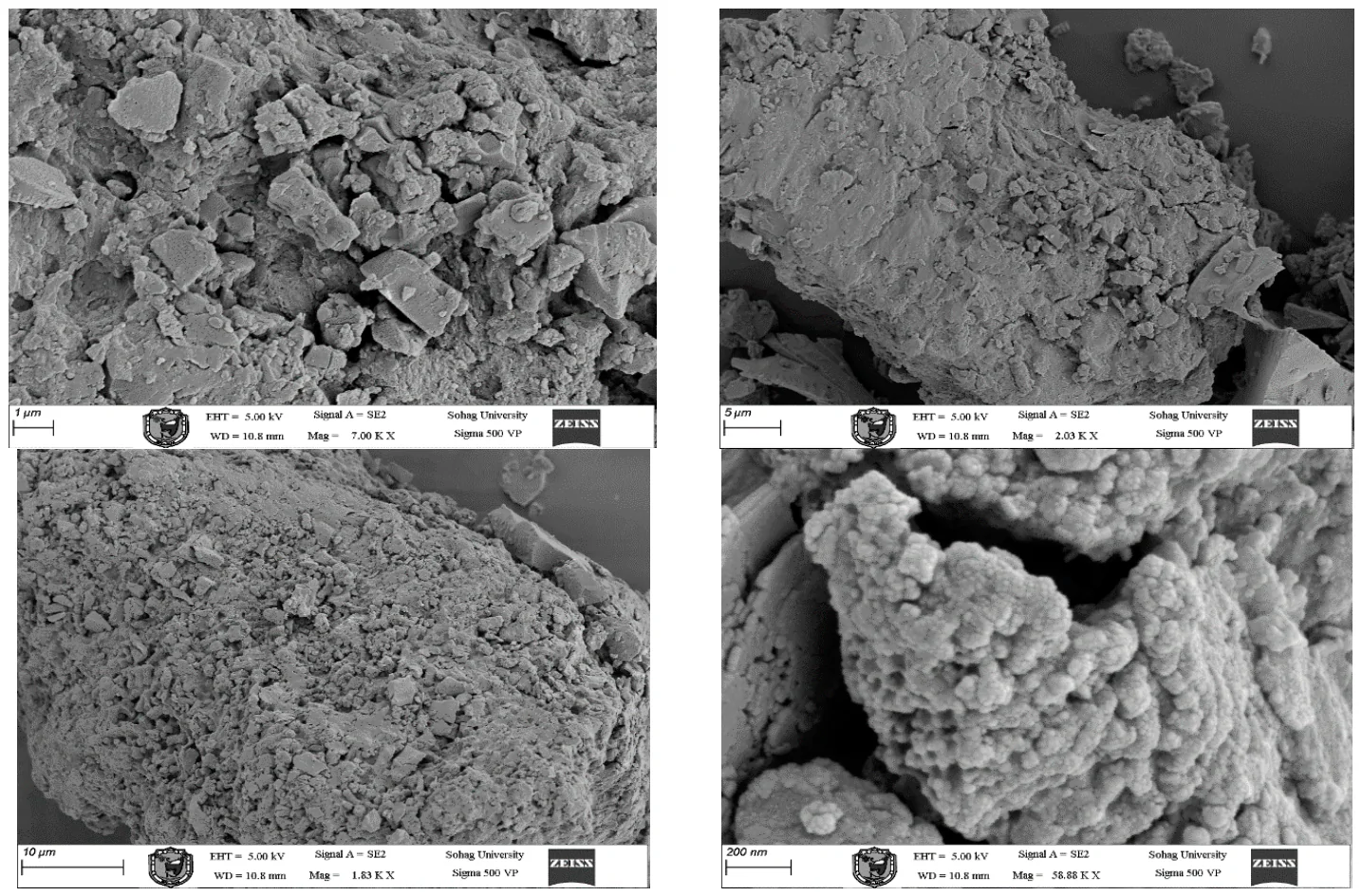
SEM of pure synthesized SeNPs from guava leaves.

**Figure 29:**
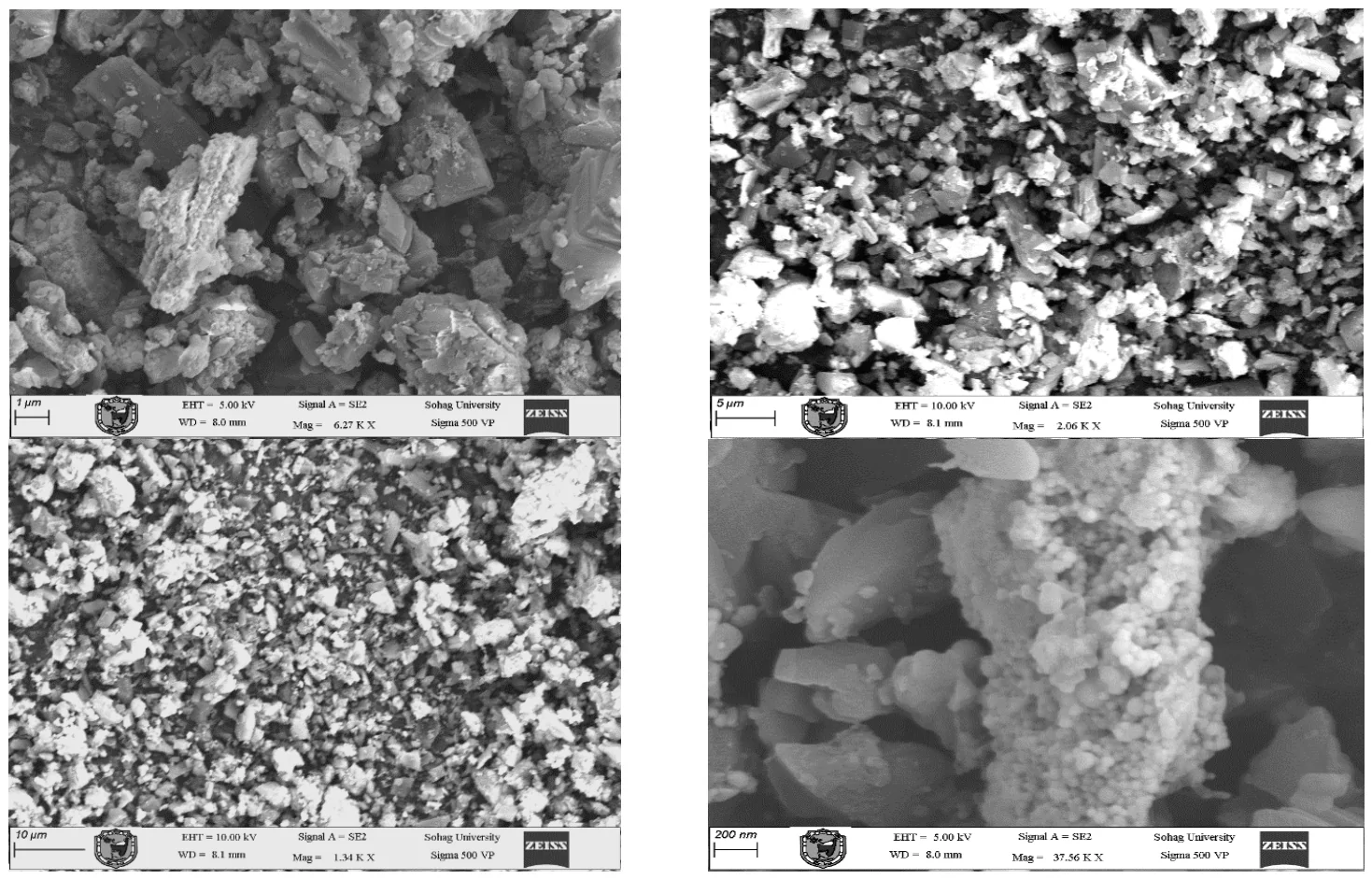
SEM of the selected Curc–SeNP–SMV.

SEM was employed to characterize the surface morphology, topographical characteristics, and microstructural features of the biogenic pure SeNPs and the selected dual-drug-loaded Curc–SeNP–SMV nanoassembly. The SEM micrographs of the pure biosynthesized SeNPs (Figure 28) revealed relatively uniform, distinct, and consolidated cluster-like microstructures, which may be associated with the natural stabilizing and capping layer of phytogenic constituents, including polyphenols and proteins, derived from the *Psidium guajava* leaf extract matrix. Comparative examination of the unloaded SeNPs (Figure 28) and the selected Curc–SeNP–SMV nanoassembly (Figure 29) revealed noticeable changes in the nanoparticle surface morphology following the incorporation of Curc and SMV. These morphological changes may be attributed to the incorporation of the two drugs and/or alterations in the surface-associated phytogenic matrix. However, these observations should be considered supportive evidence of morphological changes following drug loading rather than definitive evidence of the spatial localization of Curc and SMV within individual nanoparticles.

The selected Curc–SeNP–SMV nanoassembly has extensively texturized, well-decorated, and complex particulate networks in its micrographs. Clear deposition of organic drug molecular layers over the inorganic SeNP core changed surface roughness and created a denser crystalline packing framework without premature chemical precipitation or crystal breaking. This evident microstructural variance and dense morphological matrix confirm the spatial trapping and loading of Curc and SMV onto functionalized biogenic selenium nanovectors, making them suitable for regulated topical drug delivery systems.

The SEM comparison revealed noticeable differences in surface morphology between the unloaded SeNPs and the drug-loaded Curc–SeNP–SMV nanoparticles, with the latter exhibiting more pronounced surface irregularities and particle clustering following drug incorporation, as shown in Figure S3 (see Figure S3 in the Supplementary Materials).

SEM was used to examine the surface morphology of the selenium nanoparticles before and after drug loading. The SEM images allowed comparison of the particle morphology and surface appearance of the unloaded SeNPs and the Curc–SeNP–SMV nanoassembly. The differences observed after drug loading may be associated with the presence of the incorporated drug molecules and/or changes in the surface-associated organic matrix. Nevertheless, SEM images alone cannot definitively establish the spatial distribution of the drugs within individual nanoparticles.

#### 3.5.7. Particle Size Analysis

**Figure 30:**
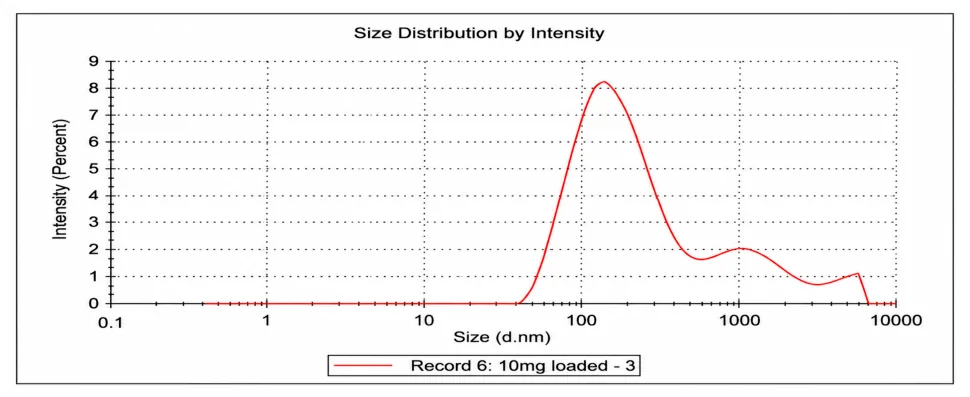
Intensity-based particle size distribution of the selected Curc–SeNP-SMV determined using dynamic light scattering.

DLS analysis of the selected Curc–SeNP–SMV nanoassembly showed a Z-average hydrodynamic diameter of 177.7 nm and a PDI of 0.41 as shown in Figure 30. The intensity-weighted size distribution showed a major population within the 100–200 nm range. Minor higher-diameter populations, observed around 1000 and 5000 nm, may reflect loose or transient particle clusters present during measurement rather than irreversible precipitation. These results describe the hydrodynamic dispersion characteristics of the optimized nanoassembly under the tested conditions and do not by themselves establish skin penetration, intracellular accumulation, or enhanced cellular uptake.

The macromolecular, multi-layered phytogenic capping corona (composed of heavy polyphenols and proteins derived from Psidium guajava leaf extract) or transient, fully reversible loose nanoconstruct clusters in suspension during analysis rather than irreversible chemical precipitation can explain the minor, lower-intensity secondary peaks at higher diameter ranges (around 1000 and 5000 nm. The Box–Behnken selected green synthesis protocol’s architectural efficiency in ensuring structural stability for complicated co-delivered topical payloads is shown by this uniform nanodistribution [91].

#### 3.5.8. Physical Appearance

Macroscopic and organoleptic examination of the selected dual-drug-loaded nanogel (Curc–SeNP–SMV embedded in Carbopol 934 matrix) showed favorable physical properties for topical and transdermal compliance. The hydrogel formulation had a smooth, elegant, and homogeneous yellowish-orange coloring, which showed the uniform molecular dispersion of the encapsulated Curc in the hydrophobic domains of the biogenic selenium nanoconstructs, as shown in Figure S2 (see Figure S2 in the Supplementary Materials). Importantly, systematic visual inspection and light microscopy scanning showed no macro-particulate matter, localized polymer aggregation, syneresis, or premature phase separation, validating the structural polymeric network’s optimal cross-linking density and thermodynamic stability. The nanogel’s structural consistency, optimal stiffness, and high microstructural uniformity confirmed that the Box–Behnken selected biogenic nanoparticles were evenly intercalated and suspended throughout the three-dimensional Carbopol 934 hydrogel configuration without compromising its rheological and aesthetic integrity.

#### 3.5.9. pH Determination

The prepared Curc–SeNP–SMV nanocomposite hydrogel exhibited a pH of 6.2 ± 0.1. This slightly acidic-to-near-neutral pH is considered appropriate for topical application and may help maintain compatibility with the skin surface. The observed pH also indicates that incorporation of the dual-loaded SeNPs into the Carbopol 934 hydrogel did not result in excessive alteration of the formulation acidity under the tested conditions [92].

#### 3.5.10. Determination of Viscosity

Carbopol 934 gel viscosity was tested at room temperature through rotational viscometer (Bohlin Instruments Ltd., Cirencester, UK). It showed a viscosity value of 51.72 ± 1.23 mP, which is appropriate for topical application. While maintaining enough residence time at the application site, the formulation demonstrated sufficient spread ability [93].

#### 3.5.11. In Vitro Drug Release from the Selected Curc–SeNP–SMV Gel

**Figure 31:**
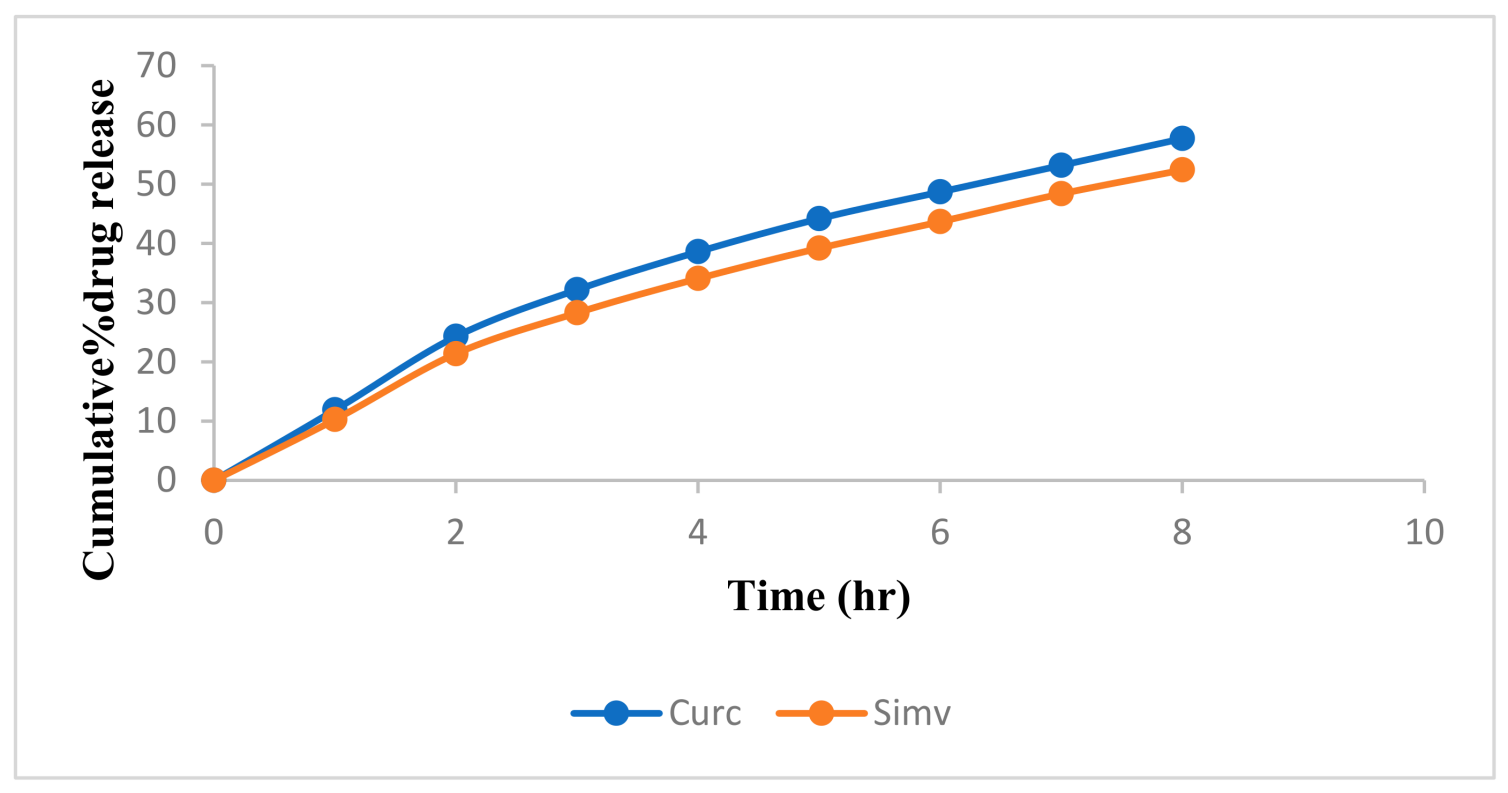
The release profile of selected Curc–SeNP–SMV gel in 30% (*v*/*v*) ethanol–PBS.

The selected Curc–SeNP–SMV gel showed a biphasic in vitro drug-release profile (Figure 31), with a mild release phase followed by a sustained release phase. SMV release was 21.4% and 52.4% after 2 and 8 h, respectively. After 2 h and 8 h, Curc released 24.3% and 57.7%, respectively.

The initial phase may be associated with the release of drug fractions that are more readily accessible at or near the nanoparticle surface, whereas the subsequent slower phase may reflect stronger interactions between the drugs and the formulation matrix and/or their diffusion and retention within the nanoparticle–polymer system. These interpretations represent plausible explanations of the observed release behavior and were not directly confirmed by mechanistic or mathematical kinetic modeling in the present study.

The nanoparticles in the Carbopol 934 gel matrix released both medicines more slowly than the selected co-loaded Curc–SeNP–SMV formulation (F9). Increased gel matrix viscosity may enhance drug transport diffusional barriers. The polymeric network generated by Carbopol 934 may also slow nanoparticle and medicinal molecule migration into the release media. The selected Curc–SeNP–SMV in the Carbopol 934 hydrogel offers a longer release pattern, which may help sustain medication availability at the application site [94].

#### 3.5.12. In Vivo Excision Wound-Healing Model

##### Topical Gel Wound-Healing Study

**Figure 32:**
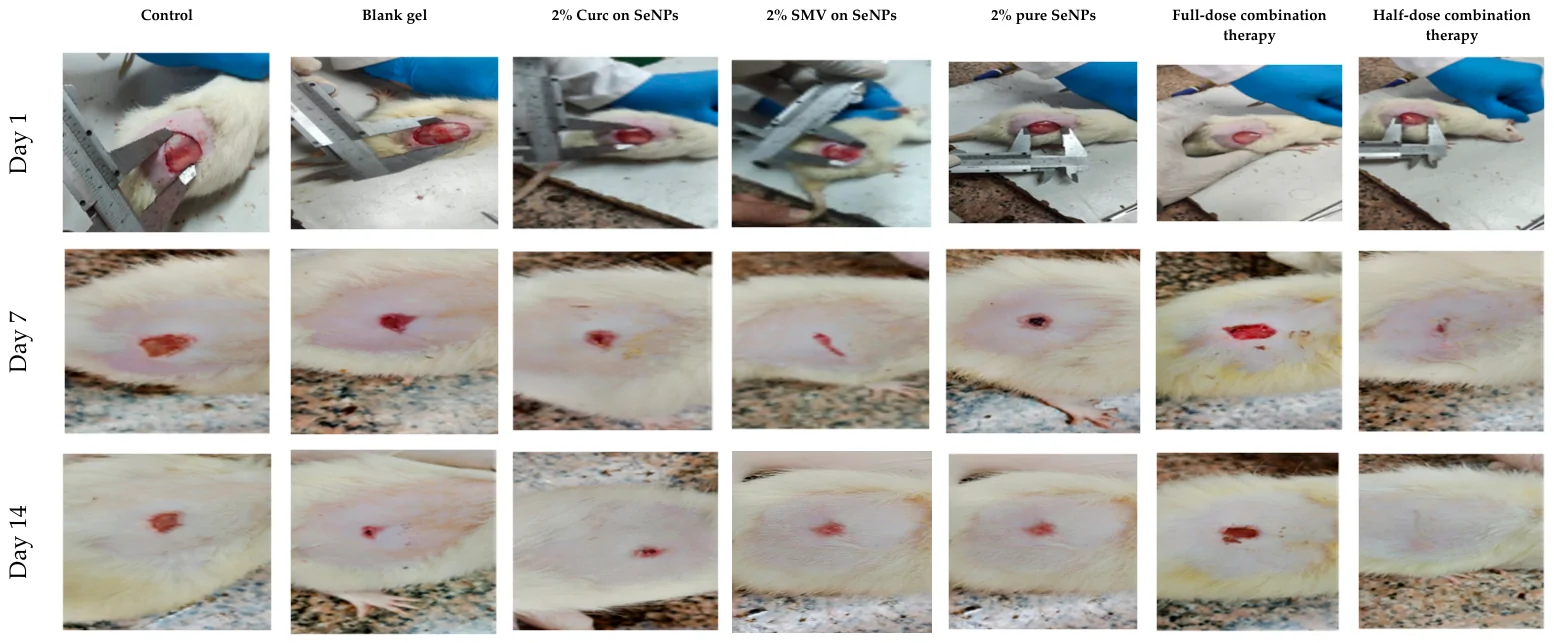
Effect of various gel formulations on epithelialization of excision wound.

**Figure 33:**
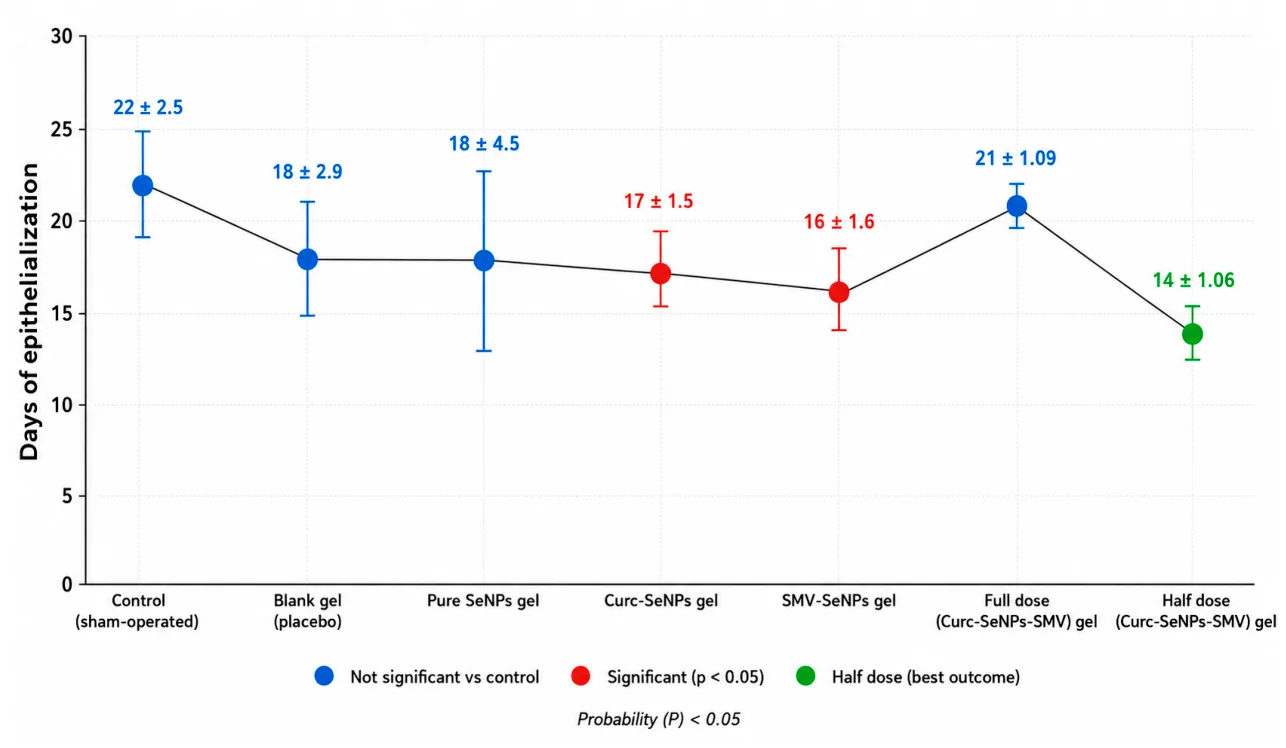
Number of days to achieve epithelialization in the excision wound model.

The impact of several topical gel formulations on wound healing was assessed by the excision wound model, focusing specifically on the duration necessary for full epithelialization [95]. Table 7 and Figure 32 and Figure 33 illustrate that variations in epithelialization duration were noted among the treatment cohorts in contrast to the sham-operated control group. The sham-operated control cohort necessitated 22 ± 2.5 days for complete epithelial regeneration. Application of the blank gel and unloaded SeNP gel diminished the epithelialization duration to 18 ± 2.9 and 18 ± 4.5 days, respectively, yet these variations were not statistically significant in comparison to the sham-operated control group. Conversely, the application of Curc–SeNP gel and SMV–SeNP gel markedly shortened the epithelialization duration to 17 ± 1.5 and 16 ± 1.6 days, respectively (*p* < 0.05), signifying an enhanced wound-healing reaction relative to the control group.

The Curc–SeNP–SMV combination formulations exhibited a nonlinear reaction in relation to dosage. The complete-dose Curc–SeNP–SMV formulation achieved total epithelialization within 21 ± 1.09 days, showing no significant difference compared to the sham-operated control group. Conversely, the half-dose Curc–SeNP–SMV formulation markedly reduced the epithelialization duration to 14 ± 1.06 days (*p* < 0.05), indicating the briefest epithelialization period across all assessed groups and a reduction of around 36% relative to the sham-operated control group. Consequently, the lower-dose combination yielded a more advantageous wound-healing result compared to the same full-dose formulation in the context of this study [96].

The enhanced wound-healing response observed with the half-dose Curc–SeNP–SMV formulation may be associated with the combined formulation of Curc and SMV within the SeNP-based delivery system. However, the present experimental design was not specifically developed to quantify pharmacological interactions between the two drugs. Therefore, the observed improvement should be interpreted as an enhanced response to the combined formulation under the tested conditions rather than as definitive evidence of a combined effect. The results further indicate that increasing the nominal dose of the two active agents did not produce a corresponding improvement in epithelialization, suggesting that the relationship between dose and wound-healing response was not simply linear.

The distinction between the half-dose and full-dose formulations may also be evaluated concerning the histological observations. The half-dose Curc–SeNP–SMV gel demonstrated enhanced epithelium regeneration and collagen arrangement without significant inflammation, while the full-dose version exhibited more notable inflammatory alterations. These results could suggest a more advantageous equilibrium between treatment exposure and local tissue reaction with the reduced dosage formulation. Nonetheless, the factors contributing to the seemingly diminished effectiveness of the full-dose formulation cannot be determined exclusively from the current data and necessitate additional research, encompassing the evaluation of local drug levels, tissue exposure, inflammatory agents, and dose–response dynamics.

In summary, the half-dose Curc–SeNP–SMV combination yielded the most advantageous wound-healing results among the tested formulations, as demonstrated by the notably reduced duration for complete epithelialization and the relatively positive histological observations. These findings endorse the promise of the integrated nanoformulation for external wound-healing uses, yet the noted advantage of the half-dose formulation ought to be regarded as a dose-specific observation rather than proof of a linear dose-dependent relationship [97].

##### Histopathological Findings

**Figure 34:**
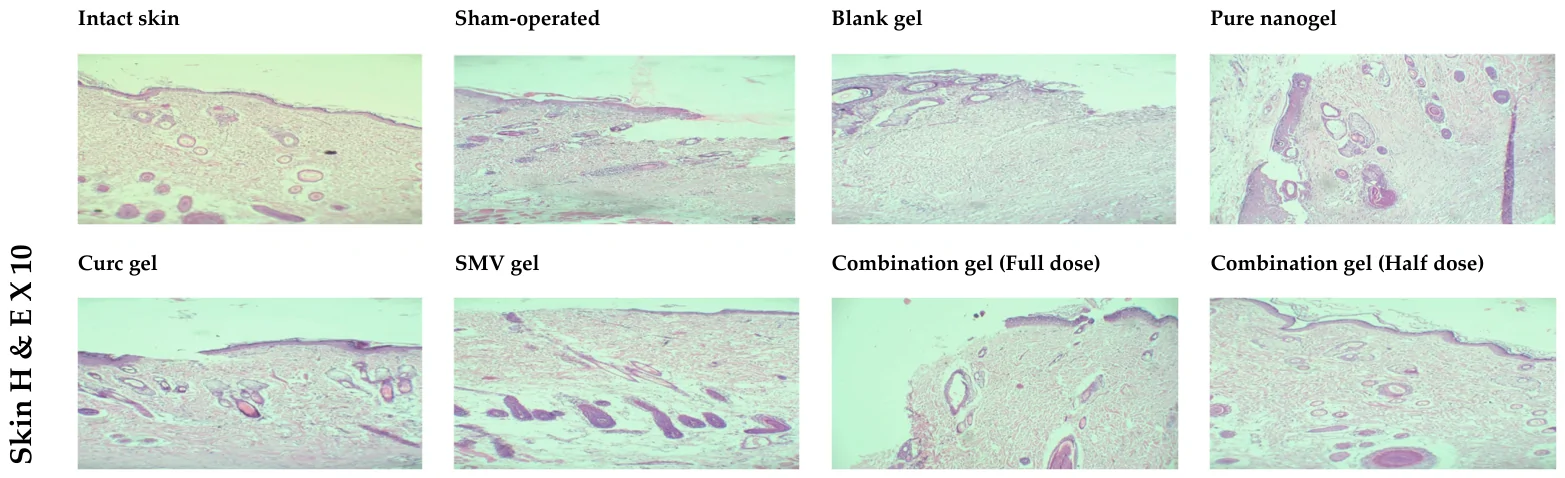
Light microscopy images of dorsal skin flaps stained with H&E at ×10 magnification. Curc: curcumin; SMV: simvastatin; SeNPs: selenium nanoparticles; Curc–SeNP–SMV: selenium nanoparticles loaded with Curc and SMV.

The histological characteristics of the examined skin tissues are illustrated in Figure 34. The unaltered control skin displayed a typical histological structure, with the five distinct layers of the epidermis easily identifiable from the basal layer to the stratum corneum. The fundamental dermis and hypodermis exhibited a typical structural arrangement, with the hypodermis revealing plentiful adipose tissue and maintaining a connection to the deeper layers.

In the sham-operated cohort, the tissue at the incision margins displayed significant histological changes, characterized by pronounced epidermal thinning and follicular atrophy. The dermis exhibited significant alterations in collagen architecture, with collagen bundles looking more plentiful, dispersed loosely, and organized in an irregular multidirectional configuration relative to the intact control skin. This modified collagen structure aligned with the tissue reorganization and scar development linked to the healing process.

The blank-gel cohort displayed histological characteristics mostly similar to those of the sham-operated cohort, suggesting minimal tissue recovery after treatment with the vehicle alone. Conversely, administering the Curc–SeNP–SMV combination gel at a reduced dosage led to significant histological enhancement. The epidermis exhibited significantly increased thickness and a more structured architecture compared to the sham-operated and blank-gel groups, resembling the look of the undamaged control skin. Furthermore, the collagen fibers within the dermis had a more structured arrangement, and the general tissue composition and scar attributes resembled those of typical, undamaged skin. These results indicate improved re-epithelialization and extracellular matrix restructuring after administering the half-dose combination formulation.

Application of the individual Curc–SeNP gel and SMV–SeNP gel resulted in significant histological enhancement relative to the sham-operated and blank-gel cohorts. Nevertheless, the degree of tissue regeneration seemed to be less significant compared to that shown with the Curc–SeNP–SMV combination gel at a reduced dosage. Despite the combination therapy exhibiting a more advantageous histology profile, the disparity between the combination and singular treatments did not attain statistical significance.

Notably, the complete dosage of Curc–SeNP–SMV combination gel elicited a significant inflammatory reaction, as demonstrated by the heightened inflammatory alterations in the analyzed tissue samples. This discovery indicates that elevating the dosage of the combined formulation over the ideal threshold may negatively impact the local tissue reaction, notwithstanding the advantageous effects noted at the reduced dosage.

The application of the unloaded nanogel led to a modest enhancement in the histological characteristics of the wound when compared to the sham-operated and blank-gel cohorts. Nevertheless, this enhancement lacked statistical significance and was less marked than that seen with the drug-loaded nanoparticle formulations.

The enhanced histological repair observed with the drug-loaded nanoparticle formulations indicates an improved tissue response compared with the control and unloaded SeNPs formulations. However, because a formal pharmacological interaction analysis was not performed, these findings cannot be used to establish whether the effects of Curc and SMV were additive or synergistic. Accordingly, the observed histological improvement is interpreted as an enhanced response to the combined formulation under the experimental conditions of the present study.

The histopathological results endorse the advantageous wound-healing capabilities of the Curc–SeNP–SMV combination gel, especially at the half-dose concentration. This mixture yielded a tissue structure that most closely resembled intact skin, demonstrating enhanced epidermal recovery and collagen arrangement while circumventing the significant inflammatory reaction seen with the full-dose combination.

A closer look at the histological sections confirms that the Curc–SeNP–SMV half-dose combination group exhibited the most advanced stage of wound healing, characterized by near-complete epidermal regeneration, well-organized dermal collagen fibers, and adnexal structures resembling those of normal, uninjured skin. In contrast, the sham-operated group, blank-gel group, pure-SeNP-gel group, single-treatment groups (Curc–SeNP gel or SMV–SeNP gel alone), and combination (full dose) group all demonstrated ongoing epidermal disruption (black arrow), visible inflammatory cell infiltration, and irregularly arranged collagen, suggesting that the healing process in these animals remained incomplete or lagged behind. These findings support the conclusion that the topical combination of Curc–SeNP–SMV (half dose) on skin flaps results in superior wound repair compared to either compound used independently or to control treatment.

##### Effect of Gel Formulations on Inflammatory and Wound-Healing Biomarkers

**Figure 35:**
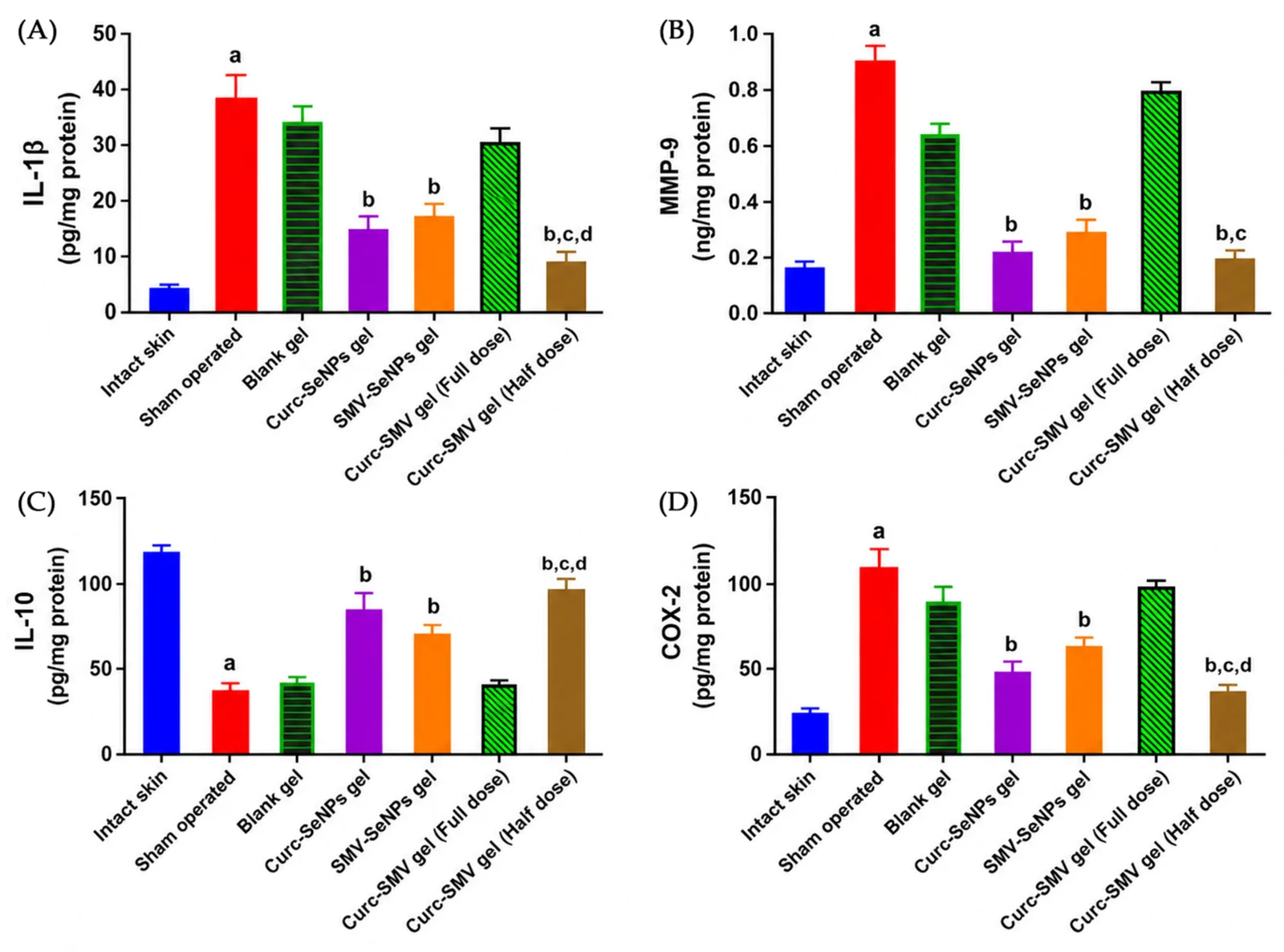
Effect of various gel formulations (Curc–SeNPs, SMV–SeNPs, and Curc–SeNP–SMV, full and half doses) on (A) IL-1β, (**B**) MMP-9, (**C**) IL-10, and (**D**) COX-2 tissue concentrations following skin excision. Data presented as means ± SD (n = 6). a: *p* < 0.05 vs. intact skin; b: *p* < 0.05 vs. sham-operated; c: *p* < 0.05 vs. Curc–SeNP–SMV (full dose) gel; d: *p* < 0.05 vs. SMV–SeNP gel.

As illustrated in Figure 35, excisional wounding significantly modified the inflammatory and tissue-remodeling characteristics of the damaged skin. In contrast to intact skin, the sham-operated group demonstrated substantially heightened tissue concentrations of the proinflammatory mediators IL-1β and COX-2, alongside increased levels of MMP-9, whereas the concentration of the anti-inflammatory cytokine IL-10 was greatly diminished. These alterations signify the marked inflammatory reaction and extracellular matrix reorganization linked to tissue damage and hindered advancement to the proliferative and remodeling stages of wound healing [98].

The various gel formulations typically diminished the inflammatory response, as indicated by decreased levels of IL-1β, MMP-9, and COX-2 alongside an increase in IL-10 compared to the sham-operated subjects. Nevertheless, the biomarker concentrations did not uniformly revert to those found in unaltered skin, suggesting that tissue regeneration remained insufficient at the examined time interval [99].

IL-1β concentrations were markedly diminished after administration of Curc–SeNPs, SMV–SeNPs, and the Curc–SeNP–SMV combination at a half dosage in comparison to the sham-operated cohort. Remarkably, the half-dose combination yielded a considerably larger decrease in IL-1β compared to each monotherapy, suggesting an enhanced anti-inflammatory effect of the combined treatment. The half-dose Curc–SeNP–SMV formulation produced a greater reduction in IL-1β than either monotherapy under the tested conditions, indicating an enhanced anti-inflammatory response to the combined formulation. However, this observation alone does not establish a combined effect between Curc and SMV, as no formal pharmacological synergy analysis was performed.

Conversely, IL-1β concentrations in animals administered the full-dose combination persisted at elevated levels, akin to those seen in the sham-operated cohort, indicating that augmenting the dosage did not yield further anti-inflammatory advantages and may have constrained the therapeutic efficacy.

The same trend was noted for MMP-9. Therapeutic intervention utilizing all active formulations markedly diminished MMP-9 concentrations in comparison to both the sham-operated and blank-gel cohorts. The half-dose Curc–SeNP–SMV combination resulted in a markedly higher decrease than the SMV–SeNPs monotherapy, although the difference when compared to Curc–SeNPs alone was not statistically significant. Notably, MMP-9 concentrations in the full-dose combination cohort persisted at elevated levels, akin to those observed in the sham-operated group. Given MMP-9’s involvement in extracellular matrix destruction and tissue remodeling, the significant decrease noted with the half-dose combination could suggest a more advantageous shift toward tissue healing and matrix stabilization [100].

Curc–SeNPs, SMV–SeNPs, and the half-dose combination of Curc–SeNP–SMV markedly diminished COX-2 levels in comparison to the sham-operated group. The half-dose combination resulted in a markedly superior inhibition of COX-2 compared to each monotherapy, hence reinforcing the augmented anti-inflammatory efficacy of the combined formulation. Conversely, COX-2 levels remained significantly heightened after treatment with the full-dose combination, resembling those of the sham-operated cohort. The sustained elevation of COX-2 in this cohort may correlate with the heightened inflammatory response noted histopathologically and could partially elucidate the suboptimal wound-healing response of the full-dose formulation.

Simultaneously with the decrease in proinflammatory mediators, IL-10 concentrations were markedly elevated after treatment with all active formulations in comparison to the sham-operated and blank-gel cohorts [101]. The half-dose Curc–SeNP–SMV combination resulted in a markedly higher increase in IL-10 compared to the separate treatment groups, suggesting a more advantageous transition toward an anti-inflammatory tissue milieu. Conversely, while the full-dose combination elicited some alteration in the inflammatory response, IL-10 levels were still markedly lower than those found in healthy skin, suggesting an inadequate recovery of the anti-inflammatory profile.

Collectively, the biomarker results indicate that the half-dose Curc–SeNP–SMV combination produced the most advantageous immunomodulatory response among the examined formulations, marked by concurrent inhibition of IL-1β, MMP-9, and COX-2 alongside an increase in IL-10. This synchronized regulation of pro- and anti-inflammatory agents may enhance the resolution of inflammation, regulate extracellular matrix remodeling, and facilitate subsequent tissue repair. Significantly, these results align with the histological findings and the expedited epithelialization noted with the half-dose combination [102].

The relatively inadequate reaction noted with the complete-dose combination is especially significant. Notwithstanding the increased concentration of active components, this formulation did not yield an equivalent advantageous biomarker profile and was linked to sustained elevations of IL-1β, MMP-9, and COX-2, alongside an inadequate recovery of IL-10. This trend, along with the heightened inflammatory alterations noted histologically and the extended epithelialization duration, indicates that the half-dose formulation would offer a more advantageous therapeutic exposure compared to the full-dose formulation. Consequently, the enhanced effectiveness of the half-dose combination may stem from an ideal equilibrium between pharmacological potency and local tissue compatibility, rather than a mere dose-dependent reaction.

Although the observed changes in IL-1β, MMP-9, COX-2, and IL-10 provide evidence of modulation of inflammatory and tissue-remodeling responses, the present study did not include tissue-localization analyses or specific markers of macrophage polarization. Therefore, the contribution of distinct macrophage phenotypes to the observed response could not be directly established. Further studies incorporating appropriate immunohistochemical or immunofluorescence markers for M1/M2 macrophage phenotypes, together with tissue-localized assessment of relevant inflammatory and wound-healing markers, are warranted to further elucidate the cellular mechanisms underlying the therapeutic response.

The present study employed a normal full-thickness excisional wound model in Wistar rats to provide an initial in vivo evaluation of the wound-healing response to a combined Curc–SeNP–SMV formulation. While this model allowed assessment of wound contraction, epithelialization, histopathological changes, and selected inflammatory biomarkers, it does not fully reproduce the pathological characteristics of chronic, diabetic, ischemic, or infected wounds. Therefore, the findings should be interpreted within the context of an acute wound-healing model. Further investigations using appropriate pathological wound models will be important to evaluate the therapeutic performance of the formulation under impaired wound-healing conditions.

## 4. Conclusions

In this study, a selenium nanoparticle (SeNP) platform that is both environmentally benign and biosustainable was successfully synthesized by employing Psidium guajava leaf extract as a natural green reducing and capping agent. This was accomplished without the use of any toxic chemical reagents. For the purpose of simultaneously co-loading hydrophobic Curc and SMV, the biogenic SeNPs were utilized as an enhanced nanocarrier technology. A Box–Behnken experimental design (BBD) was used to optimize the system, and the results showed that formulation F9 (SeNP–drug ratio of 1:1 and 30% *v*/*v* ethanol concentration) was the best system. It demonstrated exceptional entrapment efficiencies (>94.7% for SMV and >93.9% for Curc), as well as sustained in vitro drug release over a period of 8 h. Strong binding affinities of both Curc and SMV toward inflammatory enzymes COX-1 and COX-2 were demonstrated by in silico molecular docking simulations. These simulations demonstrated interaction patterns that were comparable to or superior to those of the standard reference medication diclofenac. Incorporating the selected dual-loaded nanoparticles into a 2% (*w*/*v*) Carbopol 934 hydrogel matrix resulted in the production of a nanocomposite gel that was homogeneous, physically stable, and skin-compatible. This gel is appropriate for topical application. The combined formulations were evaluated in vivo using a rat excision wound model over a period of 14 days. The in vivo findings demonstrated enhanced wound-healing responses with the Curc–SeNP–SMV combined formulation under the tested conditions, particularly at the half-dose level, as reflected by epithelialization, histopathological findings, and modulation of selected inflammatory and tissue-remodeling biomarkers. The half-dose combined formulation showed a favorable wound-healing response, whereas increasing the formulation to the full-dose level did not produce a corresponding improvement. However, the present study was not designed to formally determine pharmacological interactions between Curc and SMV. Therefore, the observed findings are interpreted as an enhanced response to the combined formulation rather than definitive evidence of additive or combined effects.

This modulation was characterized by a significant downregulation of proinflammatory factors (IL-1β, MMP-9, and COX-2) and a marked overexpression of the anti-inflammatory cytokine IL-10. Overall, the Curc–SeNP–SMV nanocomposite hydrogel demonstrated promising topical wound-healing activity under the tested experimental conditions. However, the present findings were obtained from a short-term full-thickness excisional wound model in Wistar rats and should not be directly extrapolated to chronic or ischemic wound conditions. Further studies are warranted to evaluate long-term tissue responses, formulation stability, selenium biodistribution and toxicity, additional biological mechanisms, and performance in appropriate pathological wound models before clinical translation can be considered.

## Figures and Tables

**Table 1:** Box–Behnken design of Curc–SeNP–SMV.

Formula	(SeNP:D) Ratio (C_1_)		(Eth.conc.) (C_2_)	
	Symbol	Ratio	Symbol	Conc*.(%v*/*v)*
F1	+1	1:1	0	40
F2	0	0.75:1	0	40
F3	+1	1:1	+1	50
F4	0	0.75:1	+1	50
F5	−1	0.5:1	+1	50
F6	0	0.75:1	−1	30
F7	−1	0.5:1	−1	30
F8	−1	0.5:1	+1	50
F9	+1	1:1	−1	30

**Table 2:** Observed values of responses for BBD of Curc–SeNP–SMV.

Formula	Variable Levels in Coded Form		Dependent Variables of SMV			Dependent Variables of Curc		
	C_1_	C_2_	(%EE)	Rel_2h_ (%)	Rel_8h_ (%)	(%EE)	Rel_2 h_ (%)	Rel_8 h_ (%)
F1	+1	0	91.51 ± 0.1	32.53 ± 0.2	58.24 ± 0.2	93.42 ± 0.2	37.41 ± 0.1	67.21 ± 0.1
F2	0	0	80.83 ± 0.3	30.33 ± 0.1	56.41 ± 0.4	82.93 ± 0.1	34.42 ± 0.3	65.32 ± 0.3
F3	+1	+1	86.84 ± 0.2	27.61 ± 0.3	55.67 ± 0.1	89.50 ± 0.3	30.21 ± 0.1	60.81 ± 0.1
F4	0	+1	70.34 ± 0.1	26.92 ± 0.3	53.70 ± 0.3	73.51 ± 0.2	29.80 ± 0.2	59.80 ± 0.2
F5	−1	+1	63.31 ± 0.3	25.42 ± 0.1	49.92 ± 0.1	67.42 ± 0.1	28.33 ± 0.1	58.42 ± 0.1
F6	0	−1	90.10 ± 0.4	31.31 ± 0.4	59.41 ± 0.3	92.32 ± 0.2	35.41 ± 0.3	65.83 ± 0.3
F7	−1	−1	74.16 ± 0.2	31.11 ± 0.2	55.62 ± 0.1	77.31 ± 0.4	35.10 ± 0.2	64.91 ± 0.2
F8	−1	+1	63.12 ± 0.2	26.43 ± 0.1	50.13 ± 0.2	67.30 ± 0.1	28.50 ± 0.1	58.53 ± 0.1
F9	+1	−1	94.71 ± 0.1	33.52 ± 0.2	61.63 ± 0.2	93.92 ± 0.3	37.62 ± 0.1	67.92 ± 0.2

**Table 3:** Rank order of the % entrapment efficiency of loaded Curc–SeNP–SMV.

Formula	Theoretical SMV Content (%)	Actual SMV Content (%)	Entrapment Efficiency (%EE)	Theoretical (Curc) Content (%)	Actual (Curc) Content (%)	Entrapment Efficiency (%EE)	Rank Order (RO)
F1	25	22.87	91.51 ± 0.1	25	23.35	93.42 ± 0.2	2
F2	28.57	23.09	80.83 ± 0.3	28.57	23.69	82.93 ± 0.1	5
F3	25	21.71	86.84 ± 0.2	25	22.37	89.50 ± 0.3	4
F4	28.57	20.09	70.34 ± 0.1	28.57	21.00	73.51 ± 0.2	7
F5	33.33	21.10	63.31 ± 0.3	33.33	22.47	67.42 ± 0.1	8
F6	28.57	25.74	90.10 ± 0.4	28.57	26.37	92.32 ± 0.2	3
F7	33.33	24.71	74.16 ± 0.2	33.33	25.76	77.31 ± 0.4	6
F8	33.33	21.03	63.12 ± 0.2	33.33	22.43	67.30 ± 0.1	9
F9	25	23.67	94.71 ± 0.1	25	23.48	93.92 ± 0.3	1

**Table 4:** Rank order of the release after 2 h of SMV and Curc from the loaded Curc–SeNP–SMV.

Formula	Rel_2h_ (%) SMV	Rel_2h_ (%) Curc	R.O.
F1	32.53 ± 0.2	37.41 ± 0.1	2
F2	30.33 ± 0.1	34.42 ± 0.3	5
F3	27.61 ± 0.3	30.21 ± 0.1	6
F4	26.92 ± 0.3	29.80 ± 0.2	7
F5	25.42 ± 0.1	28.33 ± 0.1	9
F6	31.31 ± 0.4	35.41 ± 0.3	3
F7	31.11 ± 0.2	35.10 ± 0.2	4
F8	26.43 ± 0.1	28.50 ± 0.1	8
F9	33.52 ± 0.2	37.62 ± 0.1	1

**Table 5:** Rank order of the release of SMV after 8 h from (Curc–SeNP–SMV).

Formula	Rel_8h_ (%) SMV	R.O.	Rel_8h_ (%) Curc	R.O.
F1	58.24 ± 0.2	3	67.21 ± 0.1	2
F2	56.41 ± 0.4	4	65.32 ± 0.3	4
F3	55.67 ± 0.1	5	60.81 ± 0.1	6
F4	53.70 ± 0.3	7	59.80 ± 0.2	7
F5	49.92 ± 0.1	9	58.42 ± 0.1	9
F6	59.41 ± 0.3	2	65.83 ± 0.3	3
F7	55.62 ± 0.1	6	64.91 ± 0.2	5
F8	50.13 ± 0.2	8	58.53 ± 0.1	8
F9	61.63 ± 0.2	1	67.92 ± 0.2	1

**Table 6:** Rank order of loaded Curc–SeNP–SMV for percentage entrapment efficiency and in vitro release after 2 h and 8 h.

Formula	(%EE) SMV and Curc	Rel_2h_ (%) SMV and Curc	Rel_8h_ (%) SMV	Rel_8h_ (%) Curc	Cumulative Ranking Order C.R.O.
F1	2	2	3	2	9
F2	5	5	4	4	18
F3	4	6	5	6	21
F4	7	7	7	7	28
F5	8	9	9	9	35
F6	3	3	2	3	11
F7	6	4	6	5	21
F8	9	8	8	8	33
F9	1	1	1	1	4

**Table 7:** Effect of (Curc–SeNPs), (SMV–SeNPs), and their combination loaded on SeNPs applied topically on wound contraction and epithelialization duration in the excision wound model.

Treatment Group	Days to Epithelialization (Mean ± SD)
Control (sham-operated)	22 ± 2.5
Blank gel (placebo)	18 ± 2.9
Pure SeNP gel	18 ± 4.5
Curc–SeNP gel	17 ± 1.5 *
SMV–SeNP gel	16 ± 1.6 *
Combination (Curc–SeNP–SMV), full dose	21 ± 1.09
Combination (Curc–SeNP–SMV), half dose	14 ± 1.06 *
Probability (P)	<0.05

Curc; curcumin, SMV; simvastatin; SeNPs; selenium nanoparticles. Data are means ± SD (n = 6). * Significantly different from sham-operated group.

## Data Availability

The original contributions presented in this study are included in the article/Supplementary Material. Further inquiries can be directed to the corresponding author.

## Supplementary Materials

The following supporting information can be downloaded at https://www.mdpi.com/article/10.3390/pharmaceutics18101257/s1. Table S1: Docking scores and interaction profiles for SMV, Curc and diclofenac against COX-1. Table S2. Docking scores and interaction profiles for SMV, Curc and diclofenac against COX-2. Table S3. ANOVA test for quadratic model for % EE of SMV. Table S4. ANOVA test for quadratic model for %EE of Curc. Table S5. ANOVA test for quadratic model for Rel_2h_% of SMV. Table S6. ANOVA test for quadratic model for Rel_2h_% of Curc. Table S7. ANOVA test for quadratic model for Rel_8h_% of SMV. Table S8. ANOVA test for quadratic model for Rel_8h_% of Curc. Table S9. Numerical optimization goals and constraints used for selecting the optimized SeNPs formulation. Table S10. Predicted and experimental response of the optimized formulation. Figure S1. Representative photographs showing (A) the prepared Psidium guajava leaf extract, (B) sodium selenite solution, and (C) the formation of selenium nanoparticles (SeNPs) following the green synthesis process. Figure S2. Representative photographs of the hydrogel before (A) and after (B) incorporation of the drug-loaded selenium nanoparticles (Curc–SeNPs–SMV), showing the macroscopic appearance and transparency of the hydrogel. Figure S3. Comparative SEM morphology of unloaded SeNPs and drug-loaded Curc–SeNPs–SMV nanoparticles at different magnifications: (A,C) unloaded SeNPs and (B,D) Curc–SeNPs–SMV nanoparticles.

## Abbreviations

Abbreviation	Definition
BBD	Box–Behnken design
RSM	response surface methodology
SeNPs	selenium nanoparticles
Curc	curcumin
SMV	simvastatin
Curc–SeNP–SMV	curcumin- and simvastatin-loaded selenium nanoparticles
SeNP:D	selenium nanoparticle-to-drug ratio
C_1_	SeNP:D ratio
Eth.conc.	ethanol concentration
E_1_	entrapment efficiency (%EE)
E_2_	cumulative drug release after 2 h (%Rel_2_ h)
E_3_	cumulative drug release after 8 h (%Rel_8_ h)
EE (%)	entrapment efficiency
%Rel_2_ h	cumulative percentage drug release after 2 h
%Rel_8_ h	cumulative percentage drug release after 8 h
FTIR	Fourier-transform infrared spectroscopy
SEM	scanning electron microscopy
DLS	dynamic light scattering
PDI	polydispersity index
XRD	X-ray diffraction
UV-vis	ultraviolet–visible spectrophotometry
PBS	phosphate-buffered saline
ANOVA	analysis of variance
p	probability value
