# Supplementary material for: Biogenic Selenium Nanoparticle-Based Nanocomposite Hydrogel for Dual Delivery of Curcumin and Simvastatin for Topical Wound Healing Applications
Source: Pharmaceutics. 2026 Oct 5;18(10):1257. doi: 10.3390/pharmaceutics18101257 (PMC13708416; doi:10.3390/pharmaceutics18101257)
Supplement: Supplementary file 1 [file pharmaceutics-18-01257-s001.zip › pharmaceutics-4576602-supplementary.pdf]

Biogenic Selenium Nanoparticle-Based Nanocomposite Hydrogel for Dual Delivery of Curcumin and Simvastatin for Topical Wound Healing Applications

Nahla A. Kasem [1,2], Hatem A. Sarhan [3], Mahmoud Mostafa [3], Heba A. Hofny [4,5], Deiaa E. Elsayed Abouzed [6], Mahmoud M. A. Elsayed [1,2] and Mahmoud Elkot Mostafa [7,8]

Table S1: Docking scores and interaction profiles for SMV, Curc and diclofenac against COX-1.

Comp.	Binding Score Kcal/ mole	Ligand atom	Residue	Interaction	Bond length (A[0])
**Simvastatin**	-8.048	O 36	ARG 374 (B)	H-acceptor	2.96
		O 38	ARG 374 (B)	H-acceptor	3.83
		O 38	ARG 374 (B)	H-acceptor	3.42
		O 44	ARG 376 (A)	H-acceptor	3.09
		C 54	PHE 142 (A)	pi-H	4.40
**Curcumin**	-6.971	O 46	GLU 140 (B)	H-donor	2.95
		O 30	HOH 16 (B)	H-acceptor	1.73
		6-ring	ASP 236 (A)	pi-H	3.91
		6-ring	SER 143 (B)	pi-H	3.45
**diclofenac**	-5.676	O 5	ARG 374 (A)	H-acceptor	2.93
		O 5	ARG 376 (A)	H-acceptor	3.47
		O 5	ARG 376 (A)	H-acceptor	3.25
		6-ring	ARG 374 (B)	pi-cation	3.67

Table S2. Docking scores and interaction profiles for SMV, Curc and diclofenac against COX-2.

Comp.	Binding Score Kcal/ mole	Ligand atom	Residue	Interaction	Bond length (A^0)
**Simvastatin**	-6.103	C 42	MET 48 (B)	H-donor	3.41
		O 1	GLN 461 (B)	H-acceptor	2.56
**Curcumin**	**-7.728**	O 30	GLU 465 (B)	H-donor	3.00
		6-ring	GLY 135 (B)	pi-H	3.93
**Diclofenac**	-6.239	O 18	TYR 130 (B)	H-acceptor	2.93
		6-ring	GLU 46 (B)	pi-H	4.68
		6-ring	CYS 47 (B)	pi-H	3.89

Table S3. ANOVA test for quadratic model for % EE of SMV.

Source	Sum of Squares	DF	Mean Square	F-value	p-value	
**Model**	1146.06	2	573.03	87.23	< 0.0001***	Highly significant
**A-SeNPs:D Ratio**	709.71	1	709.71	108.04	< 0.0001***	Highly significant
**B-Eth Conc.**	271.95	1	271.95	41.40	0.0007***	Highly Significant
**Residual**	39.41	6	6.57			
**Lack of Fit**	39.40	5	7.88	436.52	0.0363***	Highly significant
**Pure Error**	0.0181	1	0.0181			
**Total correlation**	1185.48	8				

A (SeNPs:Simv ratio), B (Eth conc.)

Highly significant (p-value <0.05) ***

Table S4. ANOVA test for quadratic model for %EE of Curc.

Source	Sum of Squares	DF	Mean Square	F-value	p-value	
**Model**	902.88	2	451.44	48.89	0.0002***	Highly significant
**A-SeNPs:D Ratio**	571.98	1	571.98	61.95	0.0002***	Highly significant
**B-Eth Conc**	203.04	1	203.04	21.99	0.0034***	Highly significant
**Residual**	55.40	6	9.23			
**Lack of Fit**	55.40	5	11.08	2215.82	0.0161***	Highly significant
**Pure Error**	0.0050	1	0.0050			
**Total correlation**	958.28	8				

A (SeNPs:Curc ratio), B (Eth conc.)

Highly significant (p-value < 0.05): ***

Table S5. ANOVA test for quadratic model for Rel_{2h}% of SMV

Source	Sum of Squares	DF	Mean Square	F-value	p-value	
**Model**	63.40	2	31.70	39.64	0.0003***	Highly significant
**A-SeNPs:D Ratio**	10.76	1	10.76	13.45	0.0105***	Highly significant
**B-Eth Conc**	44.32	1	44.32	55.43	0.0003***	Highly significant
**Residual**	4.80	6	0.7997			
**Lack of Fit**	4.30	5	0.8596	1.72	0.5199	Non significant
**Pure Error**	0.5000	1	0.5000			
**Total correlation**	68.20	8				

A (SeNPs:Simv ratio), B (Eth conc.)

Nonsignificant (p-value > 0.1) *

Highly significant (p-value < 0.05) ***

Table S6. ANOVA test for quadratic model for Rel_{2h}% of Curc.

Source	Sum of Squares	DF	Mean Square	F-value	p-value	
**Model**	111.35	5	22.27	38.96	0.0063***	Highly significant
**A-SeNPs:D Ratio**	7.93	1	7.93	13.87	0.0337***	Highly significant
**B-Eth Conc**	71.75	1	71.75	125.54	0.0015***	Highly significant
**AB**	0.0947	1	0.0947	0.1657	0.7113*	Non significant
**A^2**	0.7219	1	0.7219	1.26	0.3429*	Non significant
**B^2**	9.36	1	9.36	16.38	0.0272***	Highly significant
**Residual**	1.71	3	0.5715			
**Lack of Fit**	1.69	2	0.8473	42.37	0.1080*	Non significant
**Pure Error**	0.0200	1	0.0200			
**Total corrolation**	113.06	8				

A (SeNPs:Curc ratio), B (Eth conc.), AB (SeNPs:Curc . Eth conc.), AA (SeNPs:Curc. SeNPs:Curc), BB (Eth conc. Eth conc.).

Nonsignificant (p-value > 0.1): *

Highly significant (p-value < 0.05): ***

Table S7. ANOVA test for quadratic model for Rel_{8h}% of SMV.

Source	Sum of Squares	DF	Mean Square	F-value	p-value	
**Model**	124.14	5	24.83	1680.98	< 0.0001***	Highly Significant
**A-SeNPs:D Ratio**	42.24	1	42.24	2859.58	< 0.0001***	Highly Significant
**B-Eth Conc**	54.69	1	54.69	3702.46	< 0.0001***	Highly Significant
**AB**	0.0440	1	0.0440	2.98	0.1827**	Not Significant
**A^2**	1.58	1	1.58	106.89	0.0019***	Highly Significant
**B^2**	0.1008	1	0.1008	6.83	0.0795**	Significant
**Residual**	0.0443	3	0.0148			
**Lack of Fit**	0.0243	2	0.0122	0.6078	0.6718*	Non Significant
**Pure Error**	0.0200	1	0.0200			
**Total correlation**	124.19	8				

A (SeNPs:Simv ratio), B (Eth conc.), AB (SeNPs:Simv . Eth conc.), AA (SeNPs:Simv. SeNPs:Simv), BB (Eth conc. . Eth conc.).

Nonsignificant (p-value > 0.1): *

Significant (p-value < 0.1): **

Highly significant (p-value < 0.05): ***

Table S8. ANOVA test for quadratic model for Rel_{8h}% of Curc.

Source	Sum of Squares	DF	Mean Square	F-value	p-value	
**Model**	114.34	5	22.87	269.31	0.0004***	Highly significant
**A-SeNPs:D Ratio**	9.74	1	9.74	114.68	0.0017***	Highly significant
**B-Eth Conc**	70.25	1	70.25	827.28	< 0.0001***	Highly significant
**AB**	0.0976	1	0.0976	1.15	0.3623*	Non-significant
**A^2**	0.1513	1	0.1513	1.78	0.2742*	Non-significant
**B^2**	9.54	1	9.54	112.36	0.0018***	Highly significant
**Residual**	0.2547	3	0.0849			
**Lack of Fit**	0.2497	2	0.1249	24.97	0.1401*	Non-significant
**Pure Error**	0.0050	1	0.0050			
**Total corrolation**	114.60	8				

A (SeNPs:Curc ratio), B (Eth conc.), AB (SeNPs:Curc . Eth conc.), AA (SeNPs:Curc. SeNPs:Curc), BB (Eth conc. Eth conc.).

Nonsignificant (p-value > 0.1): *

Highly significant (p-value < 0.05): ***

Table S9. Numerical optimization goals and constraints used for selecting the optimized SeNPs formulation.

Name	Goal	Lower Limit	Upper Limit	Lower Weight	Upper Weight	Importance
**A:SeNPs:D Ratio**	is in range	-1	1	1	1	3
**B:Eth Conc**	is in range	-1	1	1	1	3
**(SMV) EE%**	maximize	63.31	94.71	1	1	3
**Rel (SMV)2 hr%**	maximize	25.42	33.52	1	1	3
**Rel (SMV) 8 hr%**	maximize	49.92	61.63	1	1	3
**(Curc) EE%**	maximize	67.42	93.92	1	1	3
**Rel (Curc) 2 hr %**	maximize	28.33	37.62	1	1	3
**Rel (Curc) 8 hr %**	maximize	58.42	67.92	1	1	3

Table S10. Predicted and experimental response of the optimized formulation

NO.	SeNPs: D Ratio	Eth Conc	%EE (SMV)	Rel2hr% (SMV)	Rel8hr % (Simv)	%EE (Curc)	Rel2hr% (Curc)	Rel 8hr % (Curc)	Desirability	
1	+1.000	-1.000	97.504	33.653	61.541	97.935	37.631	67.824	0.998	Selected
F9	+1.000	-1.000	94.71	33.52	61.63	93.92	37.62	67.92		

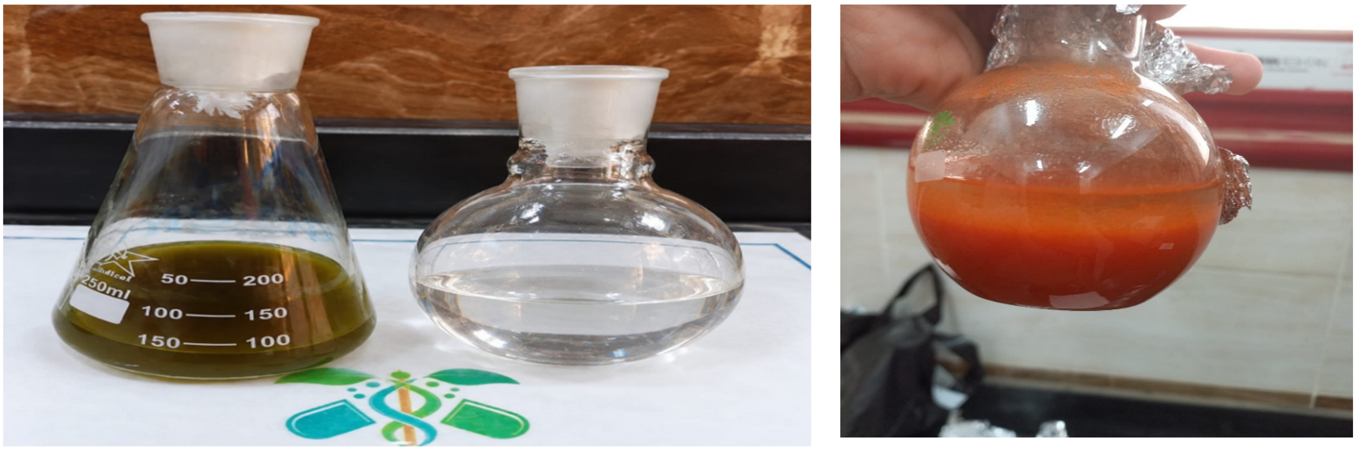

A B C

Figure S1. Representative photographs showing (A) the prepared Psidium guajava leaf extract, (B) sodium selenite solution, and (C) the formation of selenium nanoparticles (SeNPs) following the green synthesis process..

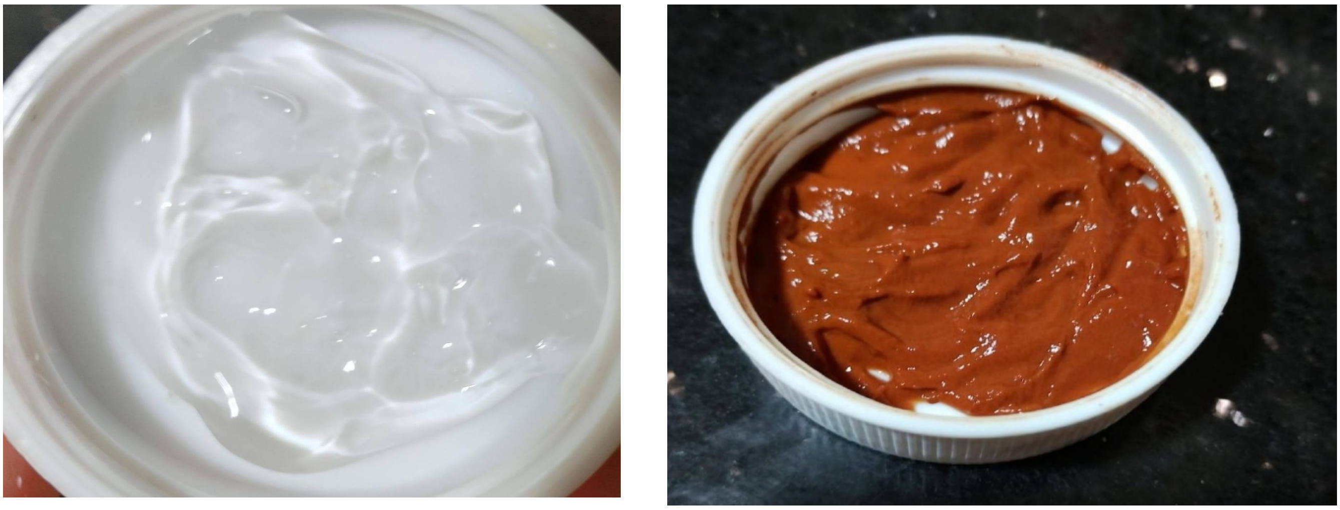

A B

Figure S2. Representative photographs of the hydrogel before (A) and after (B) incorporation of the drug-loaded selenium nanoparticles (Curc–SeNPs–SMV), showing the macroscopic appearance and transparency of the hydrogel.

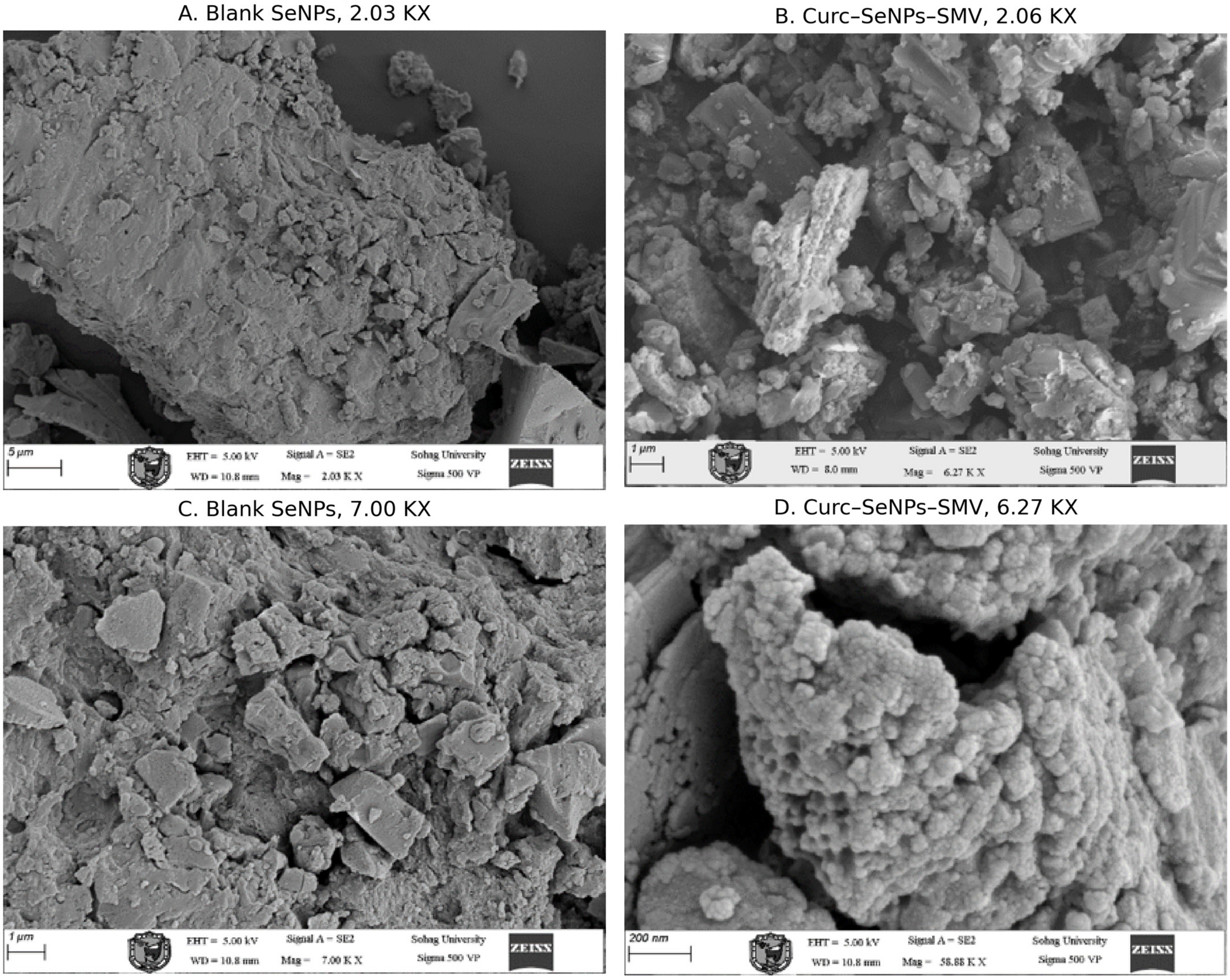

Figure S3. Comparative SEM morphology of unloaded SeNPs and drug-loaded Curc–SeNPs–SMV nanoparticles at different magnifications: (A, C) unloaded SeNPs and (B, D) Curc–SeNPs–SMV nanoparticles.
